# The Diaphragm and Lubricant Gel for Prevention of Cervical Sexually Transmitted Infections: Results of a Randomized Controlled Trial

**DOI:** 10.1371/journal.pone.0003488

**Published:** 2008-10-22

**Authors:** Gita Ramjee, Ariane van der Straten, Tsungai Chipato, Guy de Bruyn, Kelly Blanchard, Stephen Shiboski, Helen Cheng, Elizabeth Montgomery, Nancy Padian, for the MIRA team

**Affiliations:** 1 Medical Research Council, Durban, South Africa; 2 Women's Global Health Imperative, RTI International, San Francisco, California, United States of America; 3 University of Zimbabwe–UCSF Collaborative Research Programme in Women's Health, Harare, Zimbabwe; 4 Perinatal HIV Research Unit, University of the Witwatersrand, Johannesburg, South Africa; 5 Ibis Reproductive Health, Cambridge, Massachusetts, United States of America; 6 Department of Epidemiology and Biostatistics, University of California San Francisco, San Francisco, California, United States of America; 7 Center for AIDS Prevention Studies, Department of Medicine, University of California San Francisco, San Francisco, California, United States of America; University of Cape Town, South Africa

## Abstract

**Background:**

We evaluated the effectiveness of the Ortho All-Flex Diaphragm, lubricant gel (Replens®) and condoms compared to condoms alone on the incidence of chlamydial and gonococcal infections in an open-label randomized controlled trial among women at risk of HIV/STI infections.

**Methods:**

We randomized 5045 sexually-active women at three sites in Southern Africa. Participants who tested positive for curable STIs were treated prior to enrollment as per local guidelines. Women were followed quarterly and tested for *Chlamydia trachomatis* (CT) or *Neisseria gonorrhoeae* (GC) infection by nucleic-acid amplification testing (Roche Amplicor®) using first-catch urine specimens. STIs detected at follow-up visits were treated. We compared the incidence of first infection after randomization between study arms in both intent-to-treat (ITT) and per-protocol populations.

**Findings:**

Baseline demographic, behavioral and clinical characteristics were balanced across study arms. Nearly 80% of participants were under 35 years of age. Median follow-up time was 21 months and the retention rate was over 93%. There were 471 first chlamydia infections, 247 in the intervention arm and 224 in the control arm with an overall incidence of 6.2/100 woman-years (wy) (relative hazard (RH) 1.11, 95% Confidence Interval (CI): 0.93–1.33; p = 0.25) and 192 first gonococcal infections, 95 in the intervention arm and 97 in the control arm with an overall incidence of 2.4/100wy (RH 0.98, 95%CI: 0.74–1.30; p = 0.90). Per protocol results indicated that when diaphragm adherence was defined as “always use” since the last visit, there was a significant reduction in the incidence of GC infection among women randomized to the intervention arm (RH 0.61, 95%CI: 0.41–0.91, P = 0.02).

**Interpretation:**

There was no difference by study arm in the rate of acquisition of CT or GC. However, our per-protocol results suggest that consistent use of the diaphragm may reduce acquisition of GC.

**Trial Registration:**

ClinicalTrials.gov NCT00121459 [NCT00121459]

## Introduction

The feminization of HIV/AIDS in recent years has underscored the urgent need for safe and effective women-initiated HIV/STI prevention options. Sub-Saharan Africa is home to 68% of HIV-infected people worldwide, with Southern Africa being the epicenter of the pandemic [1]. Recent epidemiological studies suggest a high incidence of both HIV and STIs among women in Southern Africa coupled with low condom use. Despite intensive condom counseling, many women in these settings are unable to negotiate condom use with their male partners [2].

Concerted efforts are being made to develop new female initiated methods of HIV/STI prevention but finding a safe and effective product is challenging. Several trials of candidate microbicides have had disappointing outcomes [3]–[7].

Observational studies suggest that women who use physical barriers that cover the cervix have lower rates of infections with *Chlamydia trachomatis* (CT) and *Neisseria gonorrhoeae* (GC), which preferentially infect the cervix and upper genital tract [8]–[11]. Although it is no longer widely used, the vaginal diaphragm is a female-initiated barrier method which has been available worldwide as a contraceptive method for decades. The proposed mechanism of prevention of pregnancy and STIs is that the device, which is worn internally, acts as a physical barrier of the cervix and prevents sperm and infectious pathogens from ascending into the upper genital tract [12].

We recently reported the primary results of a phase III multisite evaluation of the diaphragm for HIV prevention: the Methods for Improving Reproductive Health in Africa (MIRA) trial of the Diaphragm and Replens® lubricant gel showed that the intervention offered no added protection against HIV when offered with a comprehensive HIV prevention package, including male condoms, safer sex counseling and treatment of sexually transmitted infections in both arms [3]. The secondary objective of the MIRA trial, reported here, was to determine the effectiveness of the diaphragm and lubricant gel in preventing CT and GC cervical infections among women at risk for sexually transmitted infections.

## Methods

The MIRA trial was conducted in Durban and Johannesburg, South Africa and Harare, Zimbabwe between 2003 and 2006. The study protocol was reviewed and approved by all participating institutions' ethical review boards, including that of the University of California, San Francisco (UCSF). After trial completion, UCSF investigators, moved to RTI International. Ethics approval from RTI International was treated as an exempt application to continue data analysis and manuscript writing. The protocol for this trial and supporting CONSORT checklist are available as supporting information; see Checklist S1 and Protocol S1.

### Participants

Women were recruited from the general community, family planning clinics and general health clinics. In Zimbabwe the study was conducted at two clinics in the greater Harare area: Epworth and Chitungwiza. In Durban, the study was also conducted at 2 clinics; in Umkomaas and Botha's Hill. In Johannesburg, the clinic was based at the Chris Hani Baragwanath Hospital in Soweto. Eligibility criteria have been described in detail elsewhere [3]. Briefly, women had to have a healthy cervix and be: sexually active; between 18 and 49 years old; HIV negative; CT and GC negative at screening, (or willing to be treated if positive); and be willing to follow all study protocol requirements. Women were excluded from the study if they: were allergic to latex; had a history of toxic shock syndrome; were pregnant and/or desired to become pregnant; had previously undergone a total hysterectomy; had clinical evidence of epithelial disruption or lesions; had undergone pelvic surgery in the past 6 weeks; reported illicit drug use; or were unable or unwilling to insert the diaphragm correctly after 5 attempts. All women provided written informed consent at screening and enrollment visits.

### Intervention

Eligible consenting women were randomized into one of two groups. In the intervention group, women received a clinician-fitted diaphragm (All Flex Arching Spring diaphragm: Ortho- McNeil Pharmaceutical, Raritan, NJ, USA), a supply of lubricant gel (Replens®, Lil Drug Store Products, Cedar Rapids, IA, USA) and male condoms. Women randomized to the control group received male condoms only. Participants in both groups received a comprehensive HIV prevention package consisting of HIV/STI pre- and post-test counseling, treatment of curable laboratory-diagnosed STIs, and intensive risk reduction counseling.

Those in the intervention arm were counseled to insert the diaphragm into the vagina at any convenient time before sexual intercourse. Women were instructed to empty an applicator of gel (2.5 g) into the dome of the diaphragm and to spread some gel onto the rim before insertion. They were asked to apply a vaginal dose of gel ≤1 h prior to intercourse for any additional sexual acts. Women were asked to leave the diaphragm *in situ* for six hours after their last act of sexual intercourse. At each visit, women were provided with a three month supply of the gel based on their self-report of coital frequency and could return to the clinic for more if desired. All women were counseled to use condoms for every sexual act as the effectiveness of the diaphragm for HIV/STI prevention was unknown. Women were given a supply of condoms, as above, and invited to come for a re-supply at any time. Women were also informed that although the diaphragm is an effective contraceptive when used with a spermicide, its efficacy for pregnancy prevention when used without a spermicide was not known. Women were counseled to use an effective contraceptive method and could receive free hormonal contraception at the study clinics.

### Randomization and Masking

Details of the randomized scheme are published elsewhere [3]. In brief, participants were randomized to assigned treatment groups using a permuted block randomization scheme, with block size stratified by study site. Consecutive participants were assigned by sequentially drawing an opaque, sealed envelope. Given the nature of the intervention, group assignment was not blinded to the participant or the clinical team. Statistical analyses were based on methods specified in an analytic plan completed prior to unblinding of the randomized treatment assignments.

### Procedures and outcomes

At the screening visit, consenting participants were offered HIV pre-test counseling before HIV and STI testing. A behavioral questionnaire on demographics and sexual behavior was administered by trained interviewers. HIV diagnostic testing was done using two rapid tests on whole blood from either finger-prick or venipuncture: Determine HIV-1/2 (Abbot Laboratories, Tokyo, Japan) and Oraquick (Orasure Technologies, Bethlehem, PA, USA). All women were counseled following their test. Those found to be HIV infected were referred to appropriate referral clinics for HIV care in their community.

For diagnosis of cervical STIs, a first catch urine sample was collected for DNA PCR testing for GC and CT (Roche Pharmaceuticals, Branchburg, NJ, USA). *Trichomonas Vaginalis* (TV) was also diagnosed with a urine sample using DNA PCR (Roche Pharmaceuticals, Branchburg., NJ, USA). Assays were performed in two laboratories, one each in Zimbabwe and South Africa. Each of the 2 laboratories participated in quality control programs locally and internationally, and was monitored annually by a study monitor who reviewed all non-negative laboratory test results. All assays were run with appropriate positive and negative controls and the laboratories participated in external proficiency testing for all available analytes. Systematic review of all positive results and tracing back to source documents detected (and rectified) clerical errors. PCR and ELISA assays were also reviewed for clustering of results that might suggest contamination and sample spill-over. Internal controls were used to validate negative PCR results. Urine specimens were stored in −20°C freezers for a maximum of 14 days before testing. Urine was the specimen of choice as a result of the use of cervical products in this trial that may have affected the quality of either cervical or vaginal samples. The GC PCR assay has been validated for use on female urine by many clinical diagnostic laboratories in the US and Western Europe. The use of a stringent definition of a positive result was implemented in this study in order to maintain high specificity [13]. Women with positive STI tests results were treated with a single (STAT) dose of 1 g of Azithromycin for CT infection and a STAT dose of 500 mg Ciprofloxacin for GC. In the third year of the trial, the latter was amended to ceftriaxone 125 mg IM for the South African sites because of emerging quinolone resistance in local GC isolates. Of all the baseline cases, 11 women with positive CT tests were untreated or treated inadequately, that is not according to protocol (10 cases in Harare, 1 case in Durban). Similarly, 3 women with baseline positive GC tests were untreated or treated inadequately (2 cases in Harare, 1 case in Durban).

At the enrollment visit, after participants provided written informed consent, a pelvic exam was performed by a study clinician. In addition, a blood sample was collected for syphilis (rapid plasma regain (RPR) and *Treponema pallidum* heamagglutinin (TPHA), Randox Laboratories, Crumlin, UK) and herpes simplex virus 2 testing (HSV2: ELISA, FOCUS Diagnostics, Cypress,CA, USA) and urine for pregnancy testing (results not presented here).

All women received education and demonstration of diaphragm insertion and removal using a pelvic model, and had the opportunity to practice using this model. Women in both arms had to demonstrate the ability to insert and remove the diaphragm to be eligible for the study. All enrolled women completed an Audio Computer Assisted Self-interview (ACASI) baseline questionnaire on demographics, sexual behavior and product use.

After enrollment each woman, regardless of randomization arm, was scheduled to return to the clinic after two weeks to address any difficulties with product use and to provide appropriate counseling about product use and risk reduction. At each quarterly follow-up visit, we conducted HIV and STI counseling and testing, treatment of curable STIs (as applicable), product adherence and risk reduction counseling and pregnancy testing. If self-report of adverse events warranted it, and at all closing visits, women received a pelvic examination. Product use was assessed by ACASI at every quarterly visit.

### Statistical considerations

The initial study sample of 4500 women was selected to provide 90% power to detect an intervention-related decrease in HIV incidence of at least 33%, significant at the 5% level. Other outcomes were not considered in the supporting calculations. Lower than expected overall incidence of HIV in the first months of follow-up led to the decision to increase the total sample size, leading to the final total sample of 5045 women. The study data was reviewed twice in November 2005 and May 2006 by an independent Data Safety Monitoring Board (DSMB), with monitoring criteria focused on the primary HIV outcome. Further details on statistical issues in study design and monitoring have been reported previously [3].

Incident infection was defined as time from enrollment to first CT or GC infection using the discrete time scale determined by the quarterly visit schedule. The primary analysis was conducted using an Intent-to-Treat (ITT) approach, which included all randomized participants. The ITT analysis was based on a Cox proportional hazards model for discrete time outcomes including a binary indicator of group assignment as the only predictor variable, and allowing for study site-specific baseline hazards. The results of the primary analysis were summarized by the estimated relative hazard comparing CT or GC incidence in the intervention group to that in the control group, with the associated 95% confidence interval (CI). Modified ITT analyses were conducted, removing from each sample the untreated or inadequately treated CT or GC cases. We used generalized estimating equation (GEE) logistic regression for longitudinal outcome measures [14] to examine site differences in levels of condom use at last sex, among women in the intervention arm. Assessment of the safety of the intervention is not reported here, as it has been previously published [3], with the proportions of participants reporting adverse events and serious adverse events, including those related to the reproductive tract, being similar across sites and between groups.

For the per-protocol analysis, two different measures of “diaphragm use” were considered: use at last sex and “always use” since last quarterly visit (both measured by ACASI). Because diaphragm and gel use were highly correlated (van der Straten et al., unpublished manuscript), for simplicity, we limited our examination of product use to that of the diaphragm. The per-protocol analysis repeated the between-group comparisons of outcomes excluding data from visits meeting the following conditions: visits for women in the intervention group where diaphragm use was not reported and visits for women in the control group where diaphragm use was reported.

## Results

A total of 10,941 women were screened. Prevalence of HIV and cervical STIs in the screening population was higher in Durban compared to Johannesburg and Harare. The HIV prevalence was 39% in Durban, 31% in Harare and 21% in Johannesburg. The prevalence of CT was 8.5%, 6.9% and 2.0% at Durban, Johannesburg and Harare, while prevalence of GC was 2.7% in Durban, 1.1% and 0.9% in Johannesburg and Harare, respectively (data not shown).

**Figure 1:**
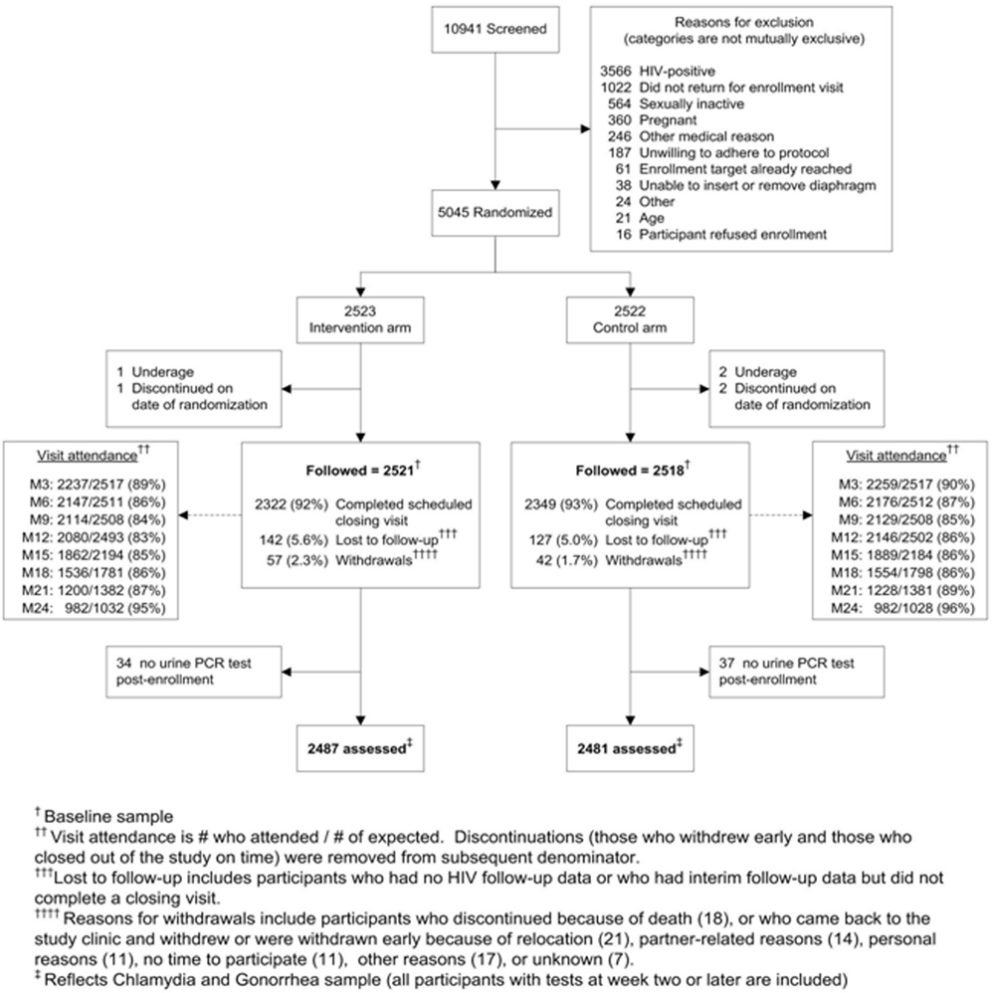
CONSORT Flow Diagram (CT/GC Trial Profile).

As shown in Figure 1, of the total screened 5,045 women were randomized. The final analytic sample included 4,968 participants who had at least one follow-up urine sample for CT/ GC analysis, including 2468, 1492 and 1008 women from Harare, Durban and Johannesburg, respectively. Rates of discontinuation (including both withdrawals and loss to follow up) were similar between both arms. The median follow-up period was 21 months and 93% of the women completed a scheduled study closing visit.

The baseline characteristics and reported sexual behavior were similar in both study groups: 31.2% reported always using condoms in the previous 3 months and two-thirds (65.4%) reported a coital frequency of 3 times or less per week; 36.2% reported using oral contraceptive pills for contraception, and 24.7% injectables, while 20.3% used a barrier method, predominantly male condoms (Table 1). Similar proportions of women (15.8%) in both groups tested positive for STI at screening (including CT, GC, syphilis and TV). In the enrolled population, the prevalence at screening for CT and GC, was 4.6% and 0.8%, respectively.

**Table 1:** Baseline socio-demographic characteristics, reproductive history and sexual behavior.

Category	Subcategory	Total (n = 5039)	Total (%)	Intervention arm (%, n = 2521)	Control arm (%, n = 2518)
***Age***	*24 years old or younger*	1933	38.4	39.6	37.2
	*25 to 34 years old*	1972	39.1	38.5	39.8
	*35 years old or older*	1133	22.5	22.0	23.0
***At least high school education***	-	2214	44.0	45.0	43.0
***Living Together***	-	3402	67.5	67.4	67.6
***Coital frequency (per week)***	*3 times or less*	3296	65.4	65.0	65.8
	*>3 times*	1743	34.6	35.0	34.2
***Regular partner circumcised***	*Yes*	1087	21.6	22.4	20.8
	*No*	2945	58.6	57.8	59.4
	*Don't know*	995	19.8	19.8	19.8
***Tested positive for STI(s)*** 1	-	794	15.8	15.4	16.2
***Tested positive for CT***	-	231	4.6	4.4	4.8
***Tested positive for GC***	-	39	0.77	0.91	0.64
***Tested positive for TV or Syphilis***	-	579	11.5	11.1	11.9
***Tested positive for HSV-2 (enrollment)***	-	2947	58.5	57.1	59.9
***High behavior risk: at least one indicator vs. none*** 2	-	1440	28.7	27.9	29.4
***High partner risk: at least one indicator vs. none*** 3	-	3447	68.6	68.7	68.5
***Frequency of condom use in past 3 months (enrollment)***	*Never*	1499	29.8	30.1	29.6
	*Sometimes*	1959	39.0	39.8	38.1
	*Always*	1570	31.2	30.2	32.3
***Current contraceptive use (screening)***	*Long term* 4	304	6.0	6.0	6.1
	*Injectable hormones*	1242	24.7	24.1	25.2
	*Pill* 5	1825	36.2	36.3	36.1
	*Barrier* 6	1024	20.3	21.1	19.5
	*Other/none*	644	12.8	12.5	13.1

1 At least one positive test for CT, GC, TV or Syphilis at screening or enrollment.

2 Indicators include: Any exchange of sex for money/food/drugs/shelter, 2 or more sexual partners within last 3 months, ever had vaginal sex under influence of drugs/alcohol in last 3 months, ever used needle for injectable drug use, ever had anal sex.

3 Indicators include: Having any sexual partners test positive for HIV, Suspect or know that regular partner had other sex partners in the last 3 months, ever had vaginal sex when partner was under influence of drugs/alcohol in last 3 months, regular partner was away from home for 1 or more months.

4 Long term methods include tubal ligation, vasectomy, IUD, implants such as Jadelle & Norplant.

5 Pill methods include combined oral contraceptive and progesterone only pills.

6 Barrier methods include male or female condoms.

**Figure 2:**
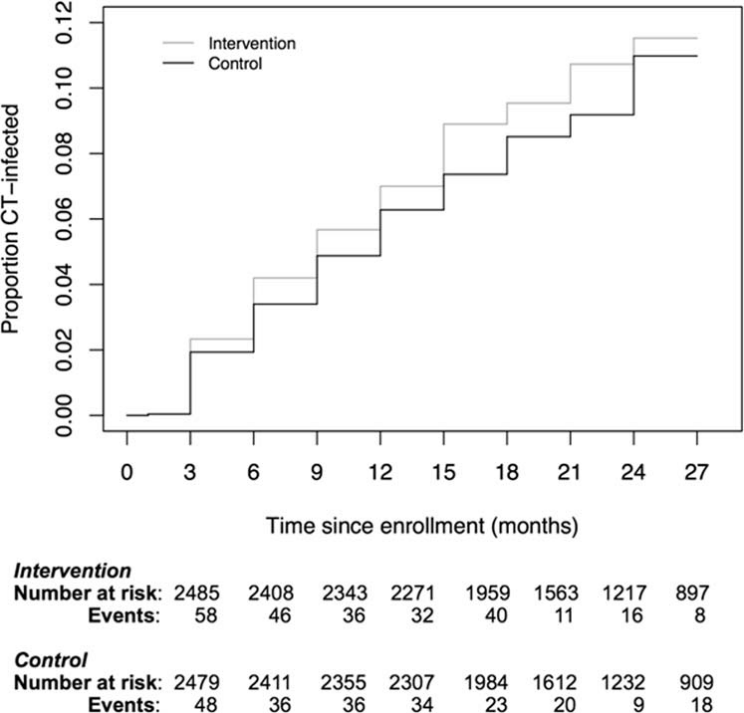
Cumulative probability of CT infection (Kaplan-Meier estimates) by group.

There were 471 first incident CT infections among 4968 participants during study follow-up, 247 among women assigned to the intervention arm and 224 among women assigned to the control arm with an overall incidence of 6.2/100 woman-years (wy) (Table 2). ITT analysis revealed no difference in the incidence of CT between treatment assignment groups (RH 1.11, 95% CI 0.93–1.33, p = 0.25). There was a significantly higher rate of CT infection in the intervention arm compared to control arm at the Zimbabwean site. The same was not observed at the South African sites. A modified ITT analysis excluding 11 participants with untreated or inadequately treated CT at baseline resulted in similar findings (data not shown). Figure 2 shows the Kaplan–Meier group-specific estimate of cumulative probability of CT acquisition.

**Table 2:** Summary of CT, overall and by site (n = 4968).

Trial Site	Study Arm	Total no. of participants	No. of events	Cumulative proportion of infections (%)*	Relative Hazard (95% CI) Intervention vs. Control arm	p-value
***All***	*Overall*	4968	471	9.48	-	-
	*Intervention*	2487	247	9.93	-	-
	*Control*	2481	224	9.03	1.11 (0.93, 1.33)	0.25
***Harare***	*Intervention*	1236	85	6.88	-	-
	*Control*	1232	53	4.30	1.63 (1.15, 2.29)	0.01
***Durban***	*Intervention*	748	98	13.10	-	-
	*Control*	744	102	13.71	0.96 (0.73, 1.27)	0.79
***Johannesburg***	*Intervention*	503	64	12.72	-	-
	*Control*	505	69	13.66	0.93 (0.66, 1.31)	0.68

* Over entire follow-up period.

**Figure 3:**
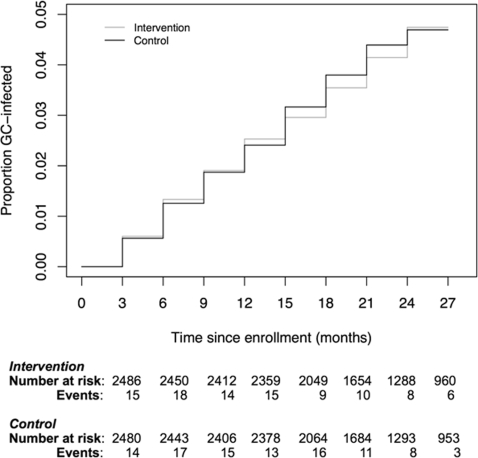
Cumulative probability of GC infection (Kaplan-Meier estimates) by group.

There were 192 first incident GC infections among 4968 participants during study follow-up, 95 infections among women assigned to the intervention arm and 97 among women assigned to the control arm with an overall incidence of 2.4/100wy (Table 3). ITT analysis also revealed no difference between the intervention and control groups with regard to the incidence of first GC infection (RH 0.98, 95% CI 0.74–1.30, p = 0.90). A modified ITT analysis excluding three participants with untreated or inadequately treated GC at baseline resulted in similar findings (data not shown). In contrast to CT, there were no significant differences observed at the site level between intervention and control arms. Figure 3 displays the Kaplan Meier curve showing the proportion of women infected with GC over time since enrollment.

**Table 3:** Summary of GC, overall and by site (n = 4968).

Trial Site	Study Arm	Total no. of participants	No. of events	Cumulative proportion of infections (%)*	Relative Hazard (95% CI) Intervention vs. Control arm	p-value
***All***	*Overall*	4968	192	3.86	-	-
	*Intervention*	2487	95	3.82	-	-
	*Control*	2481	97	3.91	0.98 (0.74, 1.30)	0.90
***Harare***	*Intervention*	1236	29	2.35	-	-
	*Control*	1232	27	2.19	1.07 (0.64, 1.81)	0.79
***Durban***	*Intervention*	748	43	5.75	-	-
	*Control*	744	42	5.65	1.03 (0.68, 1.58)	0.88
***Johannesburg***	*Intervention*	503	23	4.57	-	-
	*Control*	505	28	5.54	0.83 (0.48, 1.43)	0.50

* Over entire follow-up period.

We also conducted a per-protocol (PP) analysis to examine the possible contribution of diaphragm adherence to our results (Tables 4 and 5). In the PP analysis we observed that participants in the intervention arm had a non-significant reduction in the risk of CT acquisition when adherence was defined as diaphragm use at last sex (RH 0.90, 95% CI 0.72–1.13, p = 0.35); we found similar results when it was defined as “always use” of the diaphragm since the last quarterly visit (RH 0.84, 95% CI 0.67–1.06, p = 0.15). Of note, in both per protocol analyses, the relative hazard for CT infection at the Zimbabwean site was no longer significantly elevated. The risk of GC infection in PP analyses was also reduced, albeit non-significantly, when considering diaphragm use at last sex (RH 0.74, 95% CI 0.52–1.04, p = 0.09). When adherence was defined as “always use” of the diaphragm since the last quarterly visit, there was a significant reduction in the incidence of GC among women assigned to the intervention arm (RH 0.61, 95% CI 0.41–0.91, p = 0.02).

**Table 4:** Summary of CT per-protocol analyses (non-adherent participant visits were excluded).

Measure of diaphragm use	Trial Site	Study Arm	Total no. of participants	No. of events	Cumulative proportion of infections (%)*	Relative Hazard (95% CI) Intervention vs. Control arm	p-value
**Used at last sex**	***All***	*Overall*	4552	307	6.74	-	-
		*Intervention*	2164	130	6.01	-	-
		*Control*	2388	177	7.41	0.90 (0.72, 1.13)	0.35
	***Harare***	*Intervention*	1148	48	4.18	-	-
		*Control*	1195	43	3.60	1.24 (0.82, 1.88)	0.30
	***Durban***	*Intervention*	621	51	8.21	-	-
		*Control*	722	81	11.22	0.81 (0.57, 1.15)	0.24
	***Johannesburg***	*Intervention*	395	31	7.85	-	-
		*Control*	471	53	11.25	0.73 (0.47, 1.14)	0.17
**Always used in past three months**	***All***	*Overall*	4401	311	7.07	-	-
		*Intervention*	1959	110	5.62	-	-
		*Control*	2442	201	8.23	0.84 (0.67, 1.06)	0.15
	***Harare***	*Intervention*	1075	44	4.09	-	-
		*Control*	1228	49	3.99	1.15 (0.77, 1.73)	0.49
	***Durban***	*Intervention*	560	43	7.68	-	-
		*Control*	737	94	12.75	0.73 (0.51, 1.05)	0.09
	***Johannesburg***	*Intervention*	324	23	7.10	-	-
		*Control*	477	58	12.16	0.71 (0.44, 1.15)	0.16

* Over entire follow-up period.

**Table 5:** Summary of GC per-protocol analyses (non-adherent participant visits were excluded).

Measure of diaphragm use	Trial Site	Study Arm	Total no. of participants	No. of events	Cumulative proportion of infections (%)*	Relative Hazard (95% CI) Intervention vs. Control arm	p-value
**Used at last sex**	***All***	*Overall*	4607	138	3.00	-	-
		*Intervention*	2197	52	2.37	-	-
		*Control*	2410	86	3.57	0.74 (0.52, 1.04)	0.09
	***Harare***	*Intervention*	1161	18	1.55	-	-
		*Control*	1198	26	2.17	0.75 (0.41, 1.38)	0.36
	***Durban***	*Intervention*	631	22	3.49	-	-
		*Control*	732	38	5.19	0.76 (0.45, 1.29)	0.31
	***Johannesburg***	*Intervention*	405	12	2.96	-	-
		*Control*	480	22	4.58	0.68 (0.34, 1.38)	0.29
**Always used in past three months**	***All***	*Overall*	4428	124	2.80	-	-
		*Intervention*	1976	35	1.77	-	-
		*Control*	2452	89	3.63	0.61 (0.41, 0.91)	0.02
	***Harare***	*Intervention*	1082	15	1.39	-	-
		*Control*	1229	23	1.87	0.83 (0.43, 1.59)	0.57
	***Durban***	*Intervention*	568	12	2.11	-	-
		*Control*	739	41	5.55	0.48 (0.25, 0.91)	0.03
	***Johannesburg***	*Intervention*	326	8	2.45	-	-
		*Control*	484	25	5.17	0.60 (0.27, 1.33)	0.21

* Over entire follow-up period.

Overall, condom use at last sex in the intervention arm was reported 54% of the time, 51% in Harare, 55% in Durban and 62% Johannesburg. Site differences were statistically significant (data not shown).

## Discussion

This is the first randomized controlled trial to evaluate the effect of providing a diaphragm and lubricant gel on acquisition of cervical STIs. By ITT analysis, we found no added benefit of providing the diaphragm and lubricant gel, over and above that of condoms, on the rate of CT or GC acquisition. However, by per-protocol analysis, for those women reporting consistent (always in the past three months) diaphragm use, we observed a reduced risk of cervical gonococal infection. A similar trend, although not significant, was noted for chlamydial infection. Of note, in MIRA, condom use was lower in the intervention arm compared to the control arm [3]. This observation, combined with our finding that reported consistent diaphragm use may confer some protection against GC warrants further investigation. Additional analyses will need to adjust for possible confounding factors such as condom use that may be associated with both self reported use of diaphragms and with infection outcomes, and account for the possibility of error in self-reports of diaphragm and condom use.

A recently published observational acceptability study of the vaginal diaphragm among female sex workers and women attending sexual and reproductive health services in Kenya reported a lower incidence of gonococcal infection among women who always used the diaphragm (28.6/100wy of observation) compared to women who inconsistently used the diaphragm (48/100wy) (rate ratio 0.60, 95% CI 0.17–1.87, p = 0.34) [15].

Another RCT of the diaphragm combined with a microbicide to prevent reinfection of cervical STIs is underway in a different population [16]. If high adherence is achieved in that trial and it is demonstrated that diaphragms can prevent cervical STIs or at least GC, then there may be a public health benefit of adding the diaphragm to the prevention method mix to address the risk of STI among at-risk women.

Although exploration of site differences was beyond the scope of our analytic plan and this paper, our analyses offered no immediate explanations for the increased risk of first CT acquisition at the Harare site, observed by ITT analysis. Removal of untreated or inadequately treated baseline CT cases (most of which were in Harare) did not change the finding. Condom use at last sex in the intervention arm was lowest in Harare, but only by 4% compared to Durban, a difference which is unlikely to explain the elevated risk for CT in Harare. Furthermore there was no elevated risk for GC in Harare. Laboratory- or data-related errors were ruled out given excellent reliability performances of the laboratory and no abnormalities identified during regular laboratory monitoring visits at the site. Furthermore, laboratories were blinded to the participant study arms, so all specimens were treated the same. Finally no elevation in risk of GC was noted in Harare, and as GC and CT tests were run together, a laboratory test problem with CT is unlikely to explain this finding. Importantly, as the elevated risk for CT in the intervention arm in Harare was attenuated in the per protocol analyses, it is unlikely to be due to a harmful effect of using the diaphragm.

Prevention technologies currently undergoing clinical trials may or may not prevent STI infections in addition to HIV. Indeed, second generation microbicides containing ARV medications, pre-exposure prophylaxis, or vaccines are specific to HIV only and do not address the need for STI prevention. Although six randomized controlled trials to measure the effect of STI treatment on HIV transmission have been conducted, only the first study in Mwanza, Tanzania found nearly 40% reduction in HIV when STIs were treated through syndromic management [17], whilst the others showed no effect [18]–[22]. In addition two recent intervention trials to reduce HSV2 acquisition for HIV prevention also had disappointing outcomes [23], [24]. Nonetheless, STI prevention remains critical to prevent the significant morbidity due to non-HIV STIs, and also because these infections increase the risk of HIV acquisition.

Because many of the current (and future) HIV prevention technologies do not specifically target any STIs, we believe that a combination of prevention options may be necessary for addressing both the current HIV and STI epidemics. If future RCTs can demonstrate that the diaphragm reduces cervical infections, using a diaphragm as a physical barrier together with another HIV-specific intervention such a vaccine, second-generation microbicide, or pre-exposure prophylaxis, may have a significant public health benefit.

We could not confirm prior observational findings indicating a protective effect of the diaphragm on acquisition of cervical STIs [8], [10], [15]. However, our results suggest that consistent use of the diaphragm with lubricant gel may partially protect against some cervical infections. The biological plausibility of diaphragm use to prevent cervical STI remains, provided that the barrier methods is used correctly and consistently [12]. At a minimum, this hypothesis warrants future study.

## Supporting Information

Checklist S1 CONSORT Checklist
(0.06 MB DOC)

Protocol S1 Trial Protocol
(1.00 MB DOC)
