# Supplementary material for: The Diaphragm and Lubricant Gel for Prevention of Cervical Sexually Transmitted Infections: Results of a Randomized Controlled Trial
Source: PLoS One. 2008 Oct 22;3(10):e3488. doi: 10.1371/journal.pone.0003488 (PMC2567030; doi:10.1371/journal.pone.0003488)
Supplement: Protocol S1 — Trial Protocol (1.00 MB DOC) [file pone.0003488.s002.doc]

#
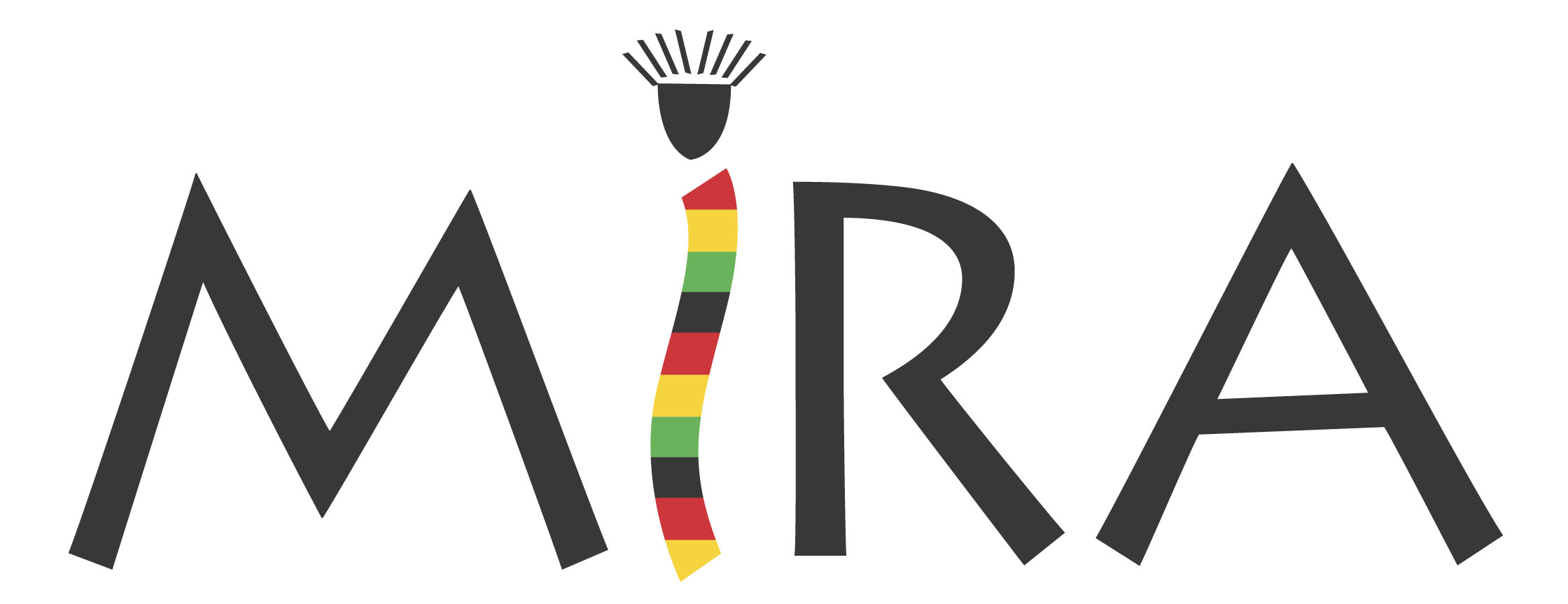


# Methods for Improving Reproductive Health in Africa

# The Latex Diaphragm to Prevent HIV Acquisition Among Women: A Female-Controlled, Physical Barrier of the Cervix

**Technical Protocol version (8.1)**

May 1, 2007

**Sponsored by**

University of California, San Francisco (UCSF)

Women’s Global Health Imperative

Department of Obstetrics, Gynecology and Reproductive Sciences

50 Beale Street, Suite 1200

UCSF Box 1224

San Francisco, CA 94105

Email: [npadian@globalhealth.ucsf.edu](mailto:npadian@globalhealth.ucsf.edu)

**Collaborating Institutions**

Ibis Reproductive Health, Cambridge, MA, USA

Medical Research Council, Durban, South Africa

University of Zimbabwe, Harare, Zimbabwe

Perinatal HIV Research Unit, Johannesburg, South Africa

**Principal Investigator**

Nancy Padian (UCSF)

**Co-Investigators**

Ariane van der Straten (UCSF)

Kelly Blanchard (Ibis Reproductive Health)

Guy de Bruyn (Perinatal HIV Research Unit)

Gita Ramjee (Medical Research Council)

Taazadza Nhemachena (University of Zimbabwe)

**Other Investigators and key staff:**

Tsungai Chipato (University of Zimbabwe)

Glenda Gray (Perinatal HIV Research Unit)

James McIntyre (Perinatal HIV Research Unit)

Steve Shiboski (UCSF)

**Project Period**

1 October 2002 to 31 May 2007

TABLE OF CONTENTS

1 Trial Abstract [5](#__RefHeading___Toc166920479)

2 Study Summary [5](#__RefHeading___Toc166920480)

3 Background [6](#__RefHeading___Toc166920481)

3.1 Why Cover the Cervix? [7](#__RefHeading___Toc166920482)

3.2 Sexually Transmitted Infections [7](#__RefHeading___Toc166920483)

3.3 Human Immuno-Deficiency Virus (HIV) [8](#__RefHeading___Toc166920484)

3.4 Preliminary Study [8](#__RefHeading___Toc166920485)

3.5 Design Rationale [8](#__RefHeading___Toc166920486)

4 Study Objectives and Design [9](#__RefHeading___Toc166920487)

4.1 Primary Objective – Effectiveness Against HIV [10](#__RefHeading___Toc166920488)

4.2 Secondary Objective 1 – Effectiveness Against Cervical Bacterial STIs [10](#__RefHeading___Toc166920489)

4.3 Secondary Objective 2 – Acceptability [10](#__RefHeading___Toc166920490)

4.4 Secondary Objective 3 – Effect of Other Reproductive Tract Infections [10](#__RefHeading___Toc166920491)

4.5 Secondary Objective 4 – Diaphragm Feasibility [11](#__RefHeading___Toc166920492)

5 Advisory Groups [11](#__RefHeading___Toc166920493)

5.1 Technical Advisory Committee (TAC) [11](#__RefHeading___Toc166920494)

5.2 Community Advisory Board (CAB) [12](#__RefHeading___Toc166920495)

5.3 Data Safety Monitoring Board (DSMB) [12](#__RefHeading___Toc166920496)

5.4 Study Monitors [13](#__RefHeading___Toc166920497)

6 Study Population [13](#__RefHeading___Toc166920498)

6.1 Study Sites [13](#__RefHeading___Toc166920499)

6.2 Eligibility Criteria [14](#__RefHeading___Toc166920500)

6.3 Exclusion Criteria [15](#__RefHeading___Toc166920501)

7 Accrual, Follow-up, and Sample Size [15](#__RefHeading___Toc166920502)

7.1 Primary Endpoint [15](#__RefHeading___Toc166920503)

7.2 Secondary Endpoints [18](#__RefHeading___Toc166920504)

8 Study Products [18](#__RefHeading___Toc166920505)

8.1 Composition of Study Products [18](#__RefHeading___Toc166920506)

8.2 Randomization [18](#__RefHeading___Toc166920507)

8.3 Product Administration Plan [19](#__RefHeading___Toc166920508)

8.4 Product Supply and Accountability [19](#__RefHeading___Toc166920509)

8.5 Compliance Counseling and Assessment [20](#__RefHeading___Toc166920510)

9 Study Procedures [20](#__RefHeading___Toc166920511)

9.1 Scheduled Visit Summary [20](#__RefHeading___Toc166920512)

9.2 Recruitment and First Contact [21](#__RefHeading___Toc166920513)

9.3 Screening Visit [21](#__RefHeading___Toc166920514)

9.4 Enrollment Visit [23](#__RefHeading___Toc166920515)

9.5 Quarterly Follow-up Visits [25](#__RefHeading___Toc166920516)

9.6 Unscheduled Visits [26](#__RefHeading___Toc166920517)

9.7 Contraceptive Use and Pregnancy [26](#__RefHeading___Toc166920518)

9.8 STI and RTI Diagnosis and Treatment [26](#__RefHeading___Toc166920519)

9.9 Cohort Retention [27](#__RefHeading___Toc166920520)

9.10 Care and Support to Study Participants [28](#__RefHeading___Toc166920521)

9.11 Recontacting Participants after Exiting the Trial [29](#__RefHeading___Toc166920522)

9.12 Use of Other Products and Medications [29](#__RefHeading___Toc166920523)

9.13 Focus Group Discussions and In-depth Interviews [30](#__RefHeading___Toc166920524)

10 Specimen Evaluation [30](#__RefHeading___Toc166920525)

10.1 Specimen Tracking and Storage [30](#__RefHeading___Toc166920526)

10.2 Specimen Collection and Testing [31](#__RefHeading___Toc166920527)

10.3 Standardization and Quality Control [32](#__RefHeading___Toc166920528)

11 Adverse Event Guidelines [32](#__RefHeading___Toc166920529)

11.1 Adverse Events and Serious Adverse Events [32](#__RefHeading___Toc166920530)

11.2 Product Interruption and Discontinuation [34](#__RefHeading___Toc166920531)

11.3 Communicable Disease Reporting Requirements [35](#__RefHeading___Toc166920532)

12 Data Collection, Management, and Analysis [35](#__RefHeading___Toc166920533)

12.1 Identification Numbers [35](#__RefHeading___Toc166920534)

12.2 Case Report Forms [36](#__RefHeading___Toc166920535)

12.3 Translation of Forms [36](#__RefHeading___Toc166920536)

12.4 Forms Tracking and Storage [36](#__RefHeading___Toc166920537)

12.5 Data Entry and Management [37](#__RefHeading___Toc166920538)

12.6 DSMB analyses [38](#__RefHeading___Toc166920539)

12.7 Final Data Analysis [38](#__RefHeading___Toc166920540)

13 Human Subjects [40](#__RefHeading___Toc166920541)

13.1 IRB Review [40](#__RefHeading___Toc166920542)

13.2 Confidentiality [40](#__RefHeading___Toc166920543)

13.3 Compensation [41](#__RefHeading___Toc166920544)

13.4 Benefits and Risks [41](#__RefHeading___Toc166920545)

13.5 Trial Closure Considerations [41](#__RefHeading___Toc166920546)

14 Administrative Procedures [42](#__RefHeading___Toc166920547)

14.1 Investigator Responsibilities [42](#__RefHeading___Toc166920548)

14.2 Study Activation [43](#__RefHeading___Toc166920549)

14.3 Study Monitoring and Independent Audits [43](#__RefHeading___Toc166920550)

14.4 Protocol Amendments and Deviations [44](#__RefHeading___Toc166920551)

14.5 Use of Information, Publishing, and Ancillary Studies [45](#__RefHeading___Toc166920552)

15 References [45](#__RefHeading___Toc166920553)

16. Appendices [48](#__RefHeading___Toc166920554)

16.1 Protocol for Bacterial Vaginosis Sub-Study [48](#__RefHeading___Toc166920555)

16.2 Protocol for Human Papillomavirus Sub-Study [52](#__RefHeading___Toc166920556)

16.3 Protocol for Social Science Component of the MIRA Trial [66](#__RefHeading___Toc166920557)

16.4 Protocol for HSV-2 Sub-Study [76](#__RefHeading___Toc166920558)

16.5 Protocol for Managing Seroconverters in the MIRA Trial [83](#__RefHeading___Toc166920559)

16.6 Protocol for Prostate-Specific Antigen (PSA) Ancillary Study [93](#__RefHeading___Toc166920560)

16.7 Protocol for Male Partner Involvement Study [95](#__RefHeading___Toc166920561)

**Abbreviations and Acronyms**

ACASI Audio Computer-Assisted Self-Interviewing

AE Adverse event

AIDS Acquired Immunodeficiency Syndrome

ARV Anti-retroviral drug

BV Bacterial vaginosis

CAB Community Advisory Board

CDC (US) Centers for Disease Control and Prevention

CRF Case Report Form

CT Chlamydia/*Chlamydia trachomatis*

DSMB Data Safety Monitoring Board

ELISA/EIA Enzyme-Linked Immuno-Sorbent Assay/Enzyme Immunoassay

FDA (US) Food and Drug Administration

NG Gonorrhea/*Neisseria gonorrhoeae*

hCG Human chorionic gonadotropin

HIV Human immuno-deficiency virus

HSV Herpes Simplex virus

HPV Human Papilloma virus

IND (USFDA) Investigational New Drug

IRB Institutional Review Board

LEEP Loop electrosurgical procedure

mm millimeters

MCC Medicines Control Council (South Africa)

N-9 Nonoxynol-9

NCR® paper No Carbon Required paper

NDA New Drug Application (USFDA)

NGO Non-governmental organization

Pap Papanicolaou

PBS Phosphate-buffered saline

UCSF University of California, San Francisco

PCR Polymerase chain reaction

PI Principal Investigator

QC Quality Control

RTI Reproductive tract infection (not necessarily sexually transmitted)

SAE Serious adverse event

SPSS® Statistical Package for Social Sciences (Chicago, IL)

STI/STD Sexually transmitted infection/sexually transmitted disease

TAC Technical Advisory Committee

TSS Toxic shock syndrome

TV Trichomoniasis/*Trichomonas vaginalis*

TP Syphilis/*Treponema pallidum*

US United States

USFDA United States Food and Drug Administration

UTI Urinary tract infection

WHO World Health Organization

# 1 Trial Abstract

This multi-site randomized, controlled trial will measure the effectiveness of the diaphragm in preventing heterosexual acquisition of HIV infection among women. This Phase III study is powered to detect effectiveness (biological efficacy combined with adherence) of 33 percent. Women in South Africa and Zimbabwe (N=5,000) at risk of contracting HIV are invited to participate and are followed for up to 24 months, with a total study duration of 4 years. All women receive safer-sex counseling, free male condoms and diagnosis and treatment of sexually transmitted infections (STIs). Half of the participants are selected randomly to receive an Ortho All-Flex® latex diaphragm and Replens® lubricant gel. We are assessing whether women using diaphragms and lubricant gel have lower rates of HIV or STI infection than do their non-diaphragm and gel counterparts. Additionally, we are investigating the long-term acceptability of the diaphragm in this study population.

# 2 Study Summary

**Study Purpose:** To determine the effectiveness of the diaphragm with Replens® lubricant gel in the reduction or prevention of the heterosexual acquisition of HIV among women.

**Study Design:** Multi-site randomized, controlled, trial with two study arms:

1) Ortho All-Flex® Arcing Spring latex diaphragm with Replens® lubricant gel, and 2) a non-diaphragm and gel control arm.

Women in both arms receive male condoms, safer-sex counseling, and diagnosis and treatment of sexually transmitted infections.

**Sample Size:** 5,000 women (2,500 per arm) seen for an average of 18 months (range of 12-24 months of follow-up).

**Study Population:** HIV-negative, sexually active, non-pregnant women.

**Visit Schedule:** Screening, enrollment, two-week post-enrollment visit, and 4 to 8 quarterly visits depending on date of enrollment. Optional visits any time for counseling, obtaining additional supplies, or other concerns.

**Study Duration:** Approximately 48 months total, with 6 months for start up activities and 6 months for analysis and write-up.

**Primary Objectives:** To determine the effectiveness of the diaphragm with Replens®

lubricant gel in preventing heterosexual transmission of HIV

among at-risk women.

**Secondary Objectives:**

1. To determine the effectiveness of the diaphragm with lubricant gel in preventing cervical infections from *Chlamydia trachomatis* (CT) and *Neisseria gonorrhoeae* (NG) among women at risk for sexually transmitted infections.

1. To assess the long-term acceptability of diaphragms with lubricant gel for use as an HIV prevention method.
2. To examine the effect of using the diaphragm and lubricant gel on *Trichomonas vaginalis* (TV) and herpes simplex virus type 2 (HSV-2) infection and to evaluate *Treponema pallidum*, TV, and HSV-2 as potential effect modifiers of a diaphragm-HIV relationship.
3. To examine the feasibility of the diaphragm and lubricant gel as a sustainable HIV prevention strategy including logistical factors such as access to product in a timely fashion, hygiene and diaphragm washing and reuse, problems associated with use, ability to hide use from partners when so desired, as well as other issues related to user feasibility.

**Study Sites:** Zimbabwe (1 site), South Africa (2 sites)

#

# 3 Background

The Joint United Nations Programme on HIV/AIDS estimates that 44 million people worldwide were living with HIV/AIDS at the end of 2004, and that about 6 million new infections occurred in 2004.^1^ The majority of new infections are transmitted through heterosexual sex. Correct and consistent male condom use has been shown to reduce the sexual transmission of HIV, but women are often unable to negotiate the use of condoms by their male partners. The female condom has been a good option for some women, but is not widely available. Furthermore, although the woman inserts the female condom, she needs a high level of cooperation from male partners to use it correctly and it cannot be used covertly. In the absence of a vaccine, the search for a safe and effective HIV-preventive whose use is controlled by women has focused to date mainly on chemical barriers, or microbicides. For a time, that priority seemed reasonable, particularly during the days when approved and available nonoxynol-9 products held promise. Today, however, even the microbicidal candidates closest to the market are still nearly a decade away. Meanwhile, new evidence has emerged that physical barriers covering the cervix might offer safe and effective protection. We are thus exploring the simple, latex diaphragm as a candidate woman-controlled HIV prevention technology, testing it in a randomized, controlled trial.

Several existing studies suggest that covering the cervix reduces risk for infection. Strong evidence that the cervix is the initial site of numerous STIs—many of which likely facilitate HIV transmission—has been well established. Most importantly, new evidence points to the cervix as the site for most HIV infections as well. Even if diaphragms prove not to be sufficiently effective by themselves, they could be ideal for holding microbicidal jellies or foams near the cervix, assuming such products reach the market in the years to come.

If effective, diaphragms would meet the key criteria established for microbicides, whether used now with a lubricant or in the future with a proven chemical protectant. Diaphragms are woman-controlled and can be used clandestinely when negotiating male condom use is impossible. They are also inexpensive, simple, reusable, accessible, acceptable, and stable under any environmental conditions, imposing no disposal problems and little difficulty with cleaning and storage. Most importantly, diaphragms have a further vital advantage over the candidate microbicides now being tested: they are already approved, backed by a major pharmaceutical, Ortho-McNeil, and marketed worldwide. Diaphragms are even already included in public-sector commodities procurement programs, such as that of USAID, making them affordable even to the poorest women in many developing countries.

## Why Cover the Cervix?

There are several reasons why the cervix might benefit from the type of protection a diaphragm could offer. Cervical cells are fragile. Like the rectum, the cervix is lined with delicate columnar epithelial cells, infected far more easily than the tough squamous epithelium of the vagina. The cervix also stands guard to the upper genital tract, itself highly susceptible to infection. For these and other reasons, the cervix is the portal of entry for many bacterial (and possibly viral) STI pathogens known to increase susceptibility to HIV.^2^ Preventing such cervical infections could decrease the likelihood of HIV acquisition. But more compelling still, new evidence points to the cervix itself as the primary “hot spot” for HIV infection, independent of its role in acquisition of other pathogens.

## Sexually Transmitted Infections

Numerous studies have confirmed that sexually transmitted infections are co-factors for HIV transmission because they enhance both the likelihood of acquisition of HIV among those infected with an STI who are HIV-negative and the infectiousness of HIV among those infected with both.^3^

Bacterial pathogens such as *Neisseria gonorrhoeae* and *Chlamydia trachomatis* preferentially infect the cervix and upper genital tract. Human papilloma virus (HPV) appears to follow the same pattern.^4^ Although previously no one had tested the diaphragm experimentally in a randomized, controlled trial against such STIs, virtually all studies examining the effect of the diaphragm on acquisition of STIs, although observational in design, have found the device to reduce infection.^5,6,7^ The diaphragm may also protect against pelvic inflammatory disease, typically a consequence of *Chlamydia trachomatis* or *Neisseria gonorrhoeae*, and even against cervical neoplasia, associated with infection from human papilloma virus.^8,9,10^

HSV-2 is a viral STI known to affect the cervix, vagina, and external genital skin.^11^ However, the cervix may be a site of higher susceptibility than vaginal or genital skin since HSV directly infects the columnar epithelial cells which are at the surface. In contrast, susceptible cells of the vagina and skin reside deeper in the epithelium and may require microtrauma to be accessible by the virus.^11^ A cervical barrier thus may be protective against HSV-2 infection as well.

A healthy vaginal flora and acidic pH appear to protect against cervico-vaginal infections. Bacterial vaginosis (BV) and *Trichomonas vaginalis* are associated with an increase in vaginal pH.^12^ Additionally, much evidence suggests that BV causes genital tract inflammation and immune activation, which possibly increases risk for HIV infection.^13, 14^

At least two other groups, including the Centers for Disease Control and Prevention (CDC) and the Population Council, are currently planning or conducting rigorous controlled studies of the diaphragm as a prevention technology against these scourges, thus taking major steps in addressing the burden bacterial STIs impose on women.

## Human Immuno-Deficiency Virus (HIV)

We hypothesize that the diaphragm’s role may extend beyond preventing other STIs to protecting women against HIV itself. If this is indeed correct, diaphragms could play a direct role in preventing HIV acquisition even in countries like Zimbabwe where the prevalence of bacterial STIs is low (*Chlamydia trachomatis* and *Neisseria gonorrhoeae* affect approximately 3% of women) and therefore cannot be fueling the HIV epidemic.

A growing number of HIV virologists and immunologists (*e.g.* Jay Levy at the University of California, San Francisco (UCSF), Myron Cohen at the University of North Carolina, and Deborah Anderson at Harvard Medical School) have demonstrated that, like other

STI pathogens, HIV also preferentially infects the cervix and upper genital tract. The nature of the epithelium of the cervix and upper genital tract offers a general explanation. But more specifically, several very recent studies reveal that, compared to the vagina, the cervix in particular and upper genital tract in general have a greater concentration of HIV-receptor sites (*i.e.* infection targets). These include CD4 and Langerhans cells, macrophages^15^ and cells bearing CCR5 and CXCR4s,^16,17,18^ and Fc-gamma receptors^19^. Consequently, physical barriers covering the cervix, such as diaphragms, should directly help prevent HIV acquisition in women.^10^

## 3.4 Preliminary Study

We completed a diaphragm acceptability study among Zimbabwean women who were unsuccessful condom users, which included documentation of problems associated with diaphragm use. Women first enrolled in a 2-month condom intervention phase. Inconsistent condom users were then enrolled into the diaphragm acceptability phase and received a diaphragm educational session, including practice on how to insert the device and use it with KY jelly, which was followed by a booster session 2 months later. Women were followed every 2 months for a total of 6 months. As of March 2003, 840 women were screened and 405 enrolled into phase I over a 12-month period. 189 women enrolled into the diaphragm acceptability phase. 181 women completed the study and the follow up rate was 96%. Diaphragm uptake was almost universal following the intervention and stayed high over time. Consistent use was reported by close to half the sample despite its stated unknown efficacy against HIV/STIs. Both surveys and exit focus group data indicated that the diaphragm was well accepted by both female and male partners and few reported side effects. Problems associated with use—mainly pain or discomfort—decreased over time as women became more familiar with the product and declined from 24% to 5% at the last follow-up visit.

## 3.5 Design Rationale

We opted for a simple, two-arm study and against adding a third arm such as lubricant only or diaphragm plus a microbicide of unknown efficacy. Such a straightforward design provides easily interpretable results and a smaller sample size compared to multi-arm studies. By choosing approved products rather than testing new products being developed to cover the cervix, our design is also less encumbered by regulatory issues.

# 4 Study Objectives and Design

We are conducting a prospective, randomized, controlled, effectiveness trial of the diaphragm with lubricant gel at three sites, one in Zimbabwe and two in South Africa. We offer all women in the trial an HIV prevention package that includes HIV testing, safer sex counseling, free male condoms, and diagnosis and treatment of STIs. At each site, women are randomized into two groups. One of these groups receives latex diaphragms with Replens® lubricant gel and counseling and training on how to use them while the other does not. We are following women quarterly for up to two years. We are measuring the incidence of HIV, cervical STIs (*Chlamydia trachomatis*, *Neisseria gonorrhoeae*), and other STIs (*Treponema pallidum*, *Trichomonas vaginalis*, and HSV-2), and will compare rates between the two groups. Because our main intervention of diaphragm with lubricant gel is offered as part of an HIV prevention package, we expect that HIV rates in both the intervention and control arms likely will be lower than those observed in the community at large. We took this into account in our sample size calculation.

We are enrolling 5,000 women who will be seen over a 12 to 24 month period, totaling approximately 7,000 woman-years of follow-up. This estimate was based on the expectations that there would be an average HIV incidence rate of 4%, that each woman we enrolled would participate on average 18 months, and that discontinuation rates would be 6-7% per year for a total of approximately 20% over the study period (3 years of field work). The expected discontinuation rates were based on on-going cohort studies at two of the sites and include true loss-to-follow-up (*i.e.* women who are lost and who have no outcome data). See section 7 below for sample size details and justification.

As described more fully in Section 9, potential study participants are screened for eligibility and enrolled if they are eligible and complete the consent process. If a woman is assigned to the diaphragm group, we ask her to insert the device into her vagina at any time that is convenient and acceptable to her and to leave it in place for at least 6 hours following sex. If she chooses to use the diaphragm continuously, we ask her to remove it at least once a day for cleaning/washing. We ask women to put lubricant gel in the dome and rim of the diaphragm at the time of insertion and to reapply gel in the vagina before each act of vaginal sex. The purpose of the gel is to provide physical coating of the vagina and cervix and to facilitate diaphragm insertion and comfort during sex. Recognizing that some women may opt to use other vaginal agents, we do not exclude women who use vaginal products other than the study lubricant together with the diaphragm. However, we collect that information as well as data on frequency of diaphragm use. At every quarterly visit we collect urine and blood specimens for laboratory data, collect behavioral information on sexual behavior (*e.g.* frequency of diaphragm, gel, and condom use) and on other behaviors (e.g. vaginal hygiene and contraceptive practices) through structured questionnaires and ACASI, and provide safer sex counseling, free male condoms and STIs treatments if needed.

After enrollment, women receive a counseling visit two weeks later, followed by 4-8 quarterly follow-up visits, depending on when they enrolled into the study. These follow-up visits are scheduled at months 3, 6, 9, 12, 15, 18, 21, and 24 (post-enrollment) with optional, additional visits as requested by participants and determined necessary by staff. Women are able to see the study staff at any time they need in order to report adverse events, request STI treatment, or obtain additional study supplies. Staff initiate optional clinic or home visits to maintain contact with hard-to-reach participants who experience difficulty complying with a less frequent study schedule.

Trial objectives and endpoints will be evaluated as described below.

## 4.1 Primary Objective – Effectiveness Against HIV

We will determine the effectiveness of the diaphragm in preventing HIV acquisition by comparing HIV incidence in the diaphragm and lubricant gel arm with the non-diaphragm and gel arm. Only HIV-negative women are enrolled. HIV testing is performed at screening, which must occur within two weeks prior to enrollment, and at each quarterly follow-up visit. We are measuring HIV infection by seroconversion using a combination of two rapid tests and a confirmatory ELISA test. If the HIV rapid tests and HIV ELISA results are discordant, an HIV DNA PCR test is used as a “tiebreaker.” To confirm that participants with no interim follow-up visit data were not HIV-infected at enrollment, HIV DNA PCR is performed on dried whole blood spot specimens stored at the enrollment visit. Those results are corroborated by RNA PCR from stored plasma from the enrollment visits if the DNA PCR was not conducted under protocol conditions.

## Secondary Objective 1 – Effectiveness Against Cervical Bacterial STIs

We will determine the effectiveness of the diaphragm in protecting against cervical bacterial STIs by comparing the incidence of *Chlamydia trachomatis* and *Neisseria gonorrhoeae* in both study arms. We test all women for these outcomes at screening, which must occur within 30 days prior to enrollment, and at each quarterly follow-up visit. *Chlamydia trachomatis* and *Neisseria gonorrhoeae* infection are tested by urine PCR tests. Recurrent infections will be defined as those with an interim negative CT/ NG PCR result between the first positive CT/ NG PCR result and a recurrent positive CT/NG PCR.

## 4.3 Secondary Objective 2 – Acceptability

As stated above (see Preliminary Study), we completed an acceptability study of the diaphragm in Zimbabwe. However, the addition of this aim to the current protocol allows us to continue to study acceptability among these women over a longer period of time. Acceptability studies at the three sites offer an opportunity to examine these issues anew and to compare findings across sites. We are assessing acceptability by asking participants in the diaphragm arm to answer detailed questions about various aspects of diaphragm acceptability during visits at month 3 and either month 24 or the woman’s closing visit if it is before 24 months. We are recording reasons for voluntary discontinuation and non-use of the diaphragm or condoms. Focus group discussions and in-depth interviews will be held with sub-groups of participants and their male partners to discuss aspects of acceptability and use dynamics in more depth (See Appendix 16.3 for the Social Science Component protocol).

## 4.4 Secondary Objective 3 – Effect of Other Reproductive Tract Infections

Because sexually transmitted infections are co-factors for HIV transmission, it is likely that these conditions will modify any potential relationship between diaphragm use and HIV. To be able to control for this, we are testing for *Treponema pallidum*, herpes simplex virus type 2, and *Trichomonas vaginalis* and will evaluate them as potential effect modifiers of a diaphragm-HIV relationship. Additionally, some of these STIs preferentially infect cervical cells (*i.e.* HSV-2) while other conditions are susceptible to changes in pH such as the buffering action produced by Replens® lubricant gel.^11^ Consequently, we have a unique opportunity to assess the protective effect of the diaphragm plus Replens® lubricant gel on these secondary outcomes. We use urine PCR tests to detect *Trichomonas vaginalis*, Enzyme-linked immuno-sorbent assay (ELISA) to detect HSV-2, and rapid plasma reagin (RPR) and confirmatory treponemal tests (TPHA) to detect *Treponema pallidum*.

## 4.5 Secondary Objective 4 – Diaphragm Feasibility

We will assess the feasibility of the diaphragm and lubricant gel as a sustainable HIV prevention strategy including logistical factors such as access to product in a timely fashion, hygiene and diaphragm washing and reuse, problems associated with use, ability to hide use from partners when so desired, as well as other issues related to user feasibility. These factors will be assessed via data that record use, requests for additional diaphragms, and problems associated with insertion and use. More specific issues regarding reuse, washing, and partners’ attitudes, will be assessed in the same focus groups that are convened to assess acceptability (See Appendix 16.3).

# 5 Advisory Groups

We have convened three advisory groups: Technical Advisory Committee (TAC), Community Advisory Board (CAB), and Data and Safety Monitoring Board (DSMB).

## Technical Advisory Committee (TAC)

As a team, we have been soliciting guidance from a small group of experts, including specialists in epidemiology, prevention, virology, immunology, clinical practice, and community advocacy. The TAC advises us on technical as well as policy matters. During the first meeting held in October 2002, the committee helped to finalize the study design and consult on issues such as selecting Replens® as the lubricant gel. Several members were also involved in selecting the third study site, the Perinatal HIV Research Unit at Chris Hani Baragwanath Hospital. They may also be consulted on various DSMB-related issues as well as interpretation of audits.

The current list of TAC members includes:

1. Chris Mauck  - [CMauck@conrad.org](mailto:CMauck@conrad.org)
2. Deborah Anderson  [-danderson@partners.org](mailto:-danderson@partners.org)
3. Janet Vail  [- jvail@path.org](mailto:-jvail@path.org)
4. Beverly Winikoff -  [bwinikoff@gynuity.org](mailto:bwinikoff@gynuity.org)
5. James Trussell  -  [trussell@princeton.edu](mailto:trussell@princeton.edu)
6. Lut Van Damme  - [lvandamme@conrad.org](mailto:lvandamme@conrad.org)
7. Marianne Callahan  - [mcallahan@conrad.org](mailto:mcallahan@conrad.org)
8. Myron Cohen  -  [mscohen@med.unc.edu](mailto:mscohen@med.unc.edu)
9. Jay Levy  - [jalevy@itsa.ucsf.edu](mailto:jalevy@itsa.ucsf.edu)
10. Chris Elias  - [celias@path.org](mailto:celias@path.org)
11. Renee Rizdon - renee.ridzon@gatesfoundation.org
12. Dorothy Patton  - [dpatton@u.washington.edu](mailto:dpatton@u.washington.edu)
13. Nicol Coetzee  -  [nick.coetzee@virgin.net](mailto:nick.coetzee@virgin.net)
14. Anwar Hoosen  -  [hoosen@medunsa.ac.za](mailto:hoosen@medunsa.ac.za)
15. Mike Mbivzo [-mbizvom@who.ch](mailto:-mbizvom@who.ch)
16. Paul Blumenthal - [pblumen@jhmi.edu](mailto:pblumen@jhmi.edu)
17. Peter Kilmarx - pbk4@cdc.gov

## Community Advisory Board (CAB)

At each site, a community advisory board (CAB) was established to meet and advise the trial team on a regular basis prior to and during the trial. The groups have clear terms of reference and are advisory in nature. Members include local researchers, NGOs, activists, health authorities, and other interested parties. The CAB process helps to ensure that the study staff are aware of and responsive to local cultural and political concerns and to develop a cadre of local colleagues who help represent the study. The process also provides a means to offer the community clear information about the study design, objectives, and parameters and to solicit suggestions from community members. Study sites have also convened wider community consultations in their trial communities as well as meetings with key policymakers, scientists, regulators, and community representatives.

## Data Safety Monitoring Board (DSMB)

The TAC was informed of and approved the final selection of DSMB members. The DSMB includes two experienced clinical trial statisticians, three physicians with appropriate clinical experience (*i.e*. gynecology and genito-urinary medicine), one epidemiologist with HIV prevention trial methodology expertise, and one women’s health advocate. All DSMB members are in general concurrence with the trial objectives, endpoints and design. None of the DSMB members are directly involved in conducting the trial. Conflicts of interest are minimized as outlined in the *FDA Guidance on the Establishment and Operation of Clinical Trial Data Monitoring Committees*. The Chair of the DSMB is selected by the PIs based on prior DSMB experience, scientific qualifications, willingness to commit an adequate amount of time to reviewing the trial data, and communication skills.

The current DSMB members include:

**Statisticians**

1. Dr. James Trussell, USA

2. Dr. Barbara Richardson, USA (Chair)

**Epidemiologist**

3. Dr. Paul Feldblum, USA

**Clinicians**

4. Dr. James Hakim, Zimbabwe

5. Dr. Paul Blumenthal, USA

6. Dr. Elizabeth Bukusi, Kenya

**Women’s Health Advocate**

7. Prudence Mabele

The DSMB originally included Khosi Xaba, a women’s health advocate from South Africa. However, Ms. Xaba withdrew herself from the board just prior to the November 2005 meeting. Ms. Xaba was replaced by Ms. Prudence Mabele in October 2005.

The trial co-PIs, consulting statisticians, and data management team will consult with the DSMB, which will then be responsible for the following duties: creating a meeting schedule and format, determining the format for data presentation, specifying who will have access to interim data and who may attend all or part of the DSMB meeting, developing procedures for assessing conflicts of interest, overseeing the method and timing of the interim analysis, and organizing report preparation and distribution.

DSMB meetings will occur by telephone unless the chair requests a face-to-face meeting. All other communication with DSMB members will be by e-mail and teleconference. DSMB meetings will include an open session, in which overall trial conduct issues will be discussed. Dr. Padian and Kelly Blanchard or their designees and possibly other trial staff will attend the open session to answer questions and raise issues for the DSMB to consider. The other co-PIs will not be permitted to attend the closed session of the meeting when trial results are discussed.

Analyses and specific duties to be conducted by the DSMB are described in section 12.6.

## 5.4 Study Monitors

Study monitors provide quality assurance to the study. Because their reviews are independent as opposed to advisory, their duties are described below in Quality Assurance (section 14.3).

# 6 Study Population

## Study Sites

(Note that all sites did not begin the study at the same time; site-specific accrual is presented below, section 7.1.2.)

### Harare, Zimbabwe

In Zimbabwe, HIV prevalence among adults increased from about 9% in 1990 to an estimated 25% in 1999.^20,21^ A population survey of close to 10,000 people, fielded between 1998 and 2000, found that women aged 17 to 24 years were four times more likely to be HIV positive than were men of the same age.^22^ Recent UCSF studies based in Harare, the capital city, (see URL http://www.uz-ucsf.co.zw) observe prevalence rates of 38% and incidence rates of between approximately 3.5%- 5% a year throughout the reproductive age span.

The UZ-UCSF Collaborative Research Programme in Harare, Zimbabwe enjoys a long-standing relationship with several family planning and reproductive health clinics in and around Harare, Zimbabwe. These clinics serve urban and peri-urban women, many of who are appropriate candidates for participation in this trial. Our Zimbabwe site has a successful track record for enrolling several hundreds to thousands of women into large epidemiologic studies. This was the first site to begin the study with enrollment starting in September 2003. Women exiting their participation in other studies are among those recruited for this study. Likewise, staff including clinicians who are trained in counseling, testing and contraceptive counseling have transitioned into this study.

The recruitment areas identified for the MIRA study are Chitungwiza, a peri-urban suburb 30 km south of Harare, and Epworth, another Harare suburb approximately 25 km to the southeast that is slightly poorer and less developed than Chitungwiza. Enrollment of women began in Chitungwiza in September 2003 and in March 2004 at Epworth.

### Durban, South Africa

Durban is located on the Indian Ocean coast in the province of KwaZulu-Natal, the province in South Africa with the highest HIV incidence. Between 1999 and 2000, HIV prevalence among women in their twenties in KwaZulu-Natal grew from 32.5% to 36.2% and incidence rates greater than 10% have been reported in certain areas.^23^

Durban's surrounding areas are the base for two groups of women who together comprise one cohort. One is peri-urban, drawn from the Umkomaas region located 60 km south of Durban. The other is rural, drawn from the Bothas Hill area located 40 km west of Durban. It is important to include rural women in the study for generalizability and reasons related to public policy, even if the cost per woman is marginally higher. Scheduled study visits take place at the MRC clinic. Enrolment for this site began in November 2003.

Other current studies in communities of this nature in areas north of Durban have shown that community-based HIV prevalence is around 45%. We estimate the prevalence of HIV in areas south and west of Durban to be around 40%. We do not have incidence data in this research naïve population. However, taking the high prevalence of HIV in RSA and sero-prevalence data from young women in KwaZulu-Natal, we estimate that the incidence in child-bearing women in these communities is around 5%.^24^

### Johannesburg, South Africa

The Perinatal HIV Research Unit (PHRU), which is based at the Chris Hani Baragwanath Hospital in Johannesburg, South Africa, was selected as the third study site. Enrollment began at PHRU in March 2004. The study population is being recruited from Soweto, an urban African township in the province of Gauteng, 15 km to the southwest of Johannesburg, with a population estimated between 1.2-2 million people in an area of 63 km^2^. The PHRU has close links with all 23 of the Soweto Primary Health Care clinics, which provide reproductive health and primary care services, and has a daily presence in 13 of the clinics. For the MIRA trial, women are recruited from family planning and well-baby clinics and from the community in Soweto. The Unit has an established Community Advisory Board and VCT services in a range of settings in Soweto and counsels and tests over 28,000 people for HIV each year. The reported HIV prevalence rate in Gauteng Province among adults ages 15-49 was 22.2% in mid-2006.^25^

## 6.2 Eligibility Criteria

To be eligible for participation, a woman must satisfy the following criteria:

- Age 18 to 49 years old
- Sexually active (coital frequency at least four times per month on average)
- HIV-negative based on testing within 14 days prior to enrollment
- *Chlamydia trachomatis-* and *Neisseria gonorrhoeae*-negative based on testing within 30 days prior to enrollment or, if positive, completes treatment before enrollment
- Have a healthy cervix as assessed by naked-eye speculum exam at enrollment.
- Planning to live in the study area for the next 24 months
- Willing to be randomly assigned either to use or not use a latex diaphragm with lubricant gel during her participation in the study and to follow the protocol, including being tested for HIV and STIs
- Willing and able to give informed consent

## 6.3 Exclusion Criteria

The following criteria will exclude a woman from study participation:

- Known sensitivity or allergy to latex
- History of TSS (as suggested by current labeling for diaphragm use)
- Currently pregnant or desiring to become pregnant in the next two years
- No cervix (total hysterectomy)
- Refuses treatment for diagnosed or suspected current STI and/ RTI requiring treatment at enrollment
- Within six weeks of any pelvic surgery or last pregnancy outcome at the time of enrollment.
- Presence of clinically apparent lesion(s) with epithelial disruption at enrollment (may enroll after lesion(s) has healed, if otherwise eligible)
- Injected illicit drugs in the 12 months prior to screening and enrollment
- Blood transfusion or received blood products in 3 months prior to screening and enrollment
- Unable or unwilling to insert the diaphragm correctly
- Participation in any other clinical trial including those of a vaginal product or barrier contraceptive; verification by comparing participant lists to other concurrently running research studies may be conducted if necessary
- Unable to speak English, Zulu, Shona, or Sotho
- Other conditions that, in the opinion of the investigator, would constitute contraindications to participation in the study or would compromise ability to comply with the study protocol, such as a major psychiatric disorder
- Does not meet eligibility criteria within 5 screening or 5 enrollment visits

# 7 Accrual, Follow-up, and Sample Size

## 7.1 Primary Endpoint

**7.1.1 Required sample size**

Sample size calculations are made by comparing HIV incidence rates between study arms using a log rank test stratified by study site and using approximate methods described by Lachin and Foulkes. ^26^We initially assumed that participants will be recruited over a period of 2 years, that the duration of the study would be 3 years, and that annual HIV incidence in the non-diaphragm arm (control group) would be approximately 4%, 5% and 3.5% at the Harare, Durban, and Johannesburg sites respectively. We further assumed that these sites would contribute 49%, 29%, and 22% of the total sample respectively and that discontinuation rates, including lost to follow up and true discontinuations, would not exceed approximately 7% per year at all sites or a total of approximately 20% over the three-years field implementation period. With these assumptions we required a total of 4500 women to have 90% power to detect a 33% treatment-related reduction in incidence as significant at the 5% level, assuming a two-sided alternative. This sample size and the assumption of constant rates of recruitment and censoring implies that approximately 4000 woman years of follow-up will be required per arm to detect a treatment-related reduction in incidence of 33%. This corresponds to a total of approximately 293 total serconversions in both arms combined. The following table presents enrollment goals and expected numbers of seroconversions by site.

**Table 4***: Approximate numbers of women to be enrolled to reach required number (293) seroconversions. We assume approximately 20% discontinuation rate at each site over the entire study period.*

|  | **Enrolled** | **Estimated annual incidence (overall)** | **Minimum estimated**  **Infections** |
| --- | --- | --- | --- |
| Harare | 2200 | 4% | 134 |
| Durban | 1300 | 5.0% | 99 |
| Johannesburg | 1000 | 3.5% | 60 |
| **Total** | **4500** | **~4.0%** | **293** |

If incidence is actually higher than assumed here, the diaphragm actually confers more than 33% protection, or the discontinuation rate is lower, it may be possible to end the study early. This decision will be made via stopping rules that will be considered during the interim analysis conducted by our DSMB (section 12.6. below).

In April 2005, the study statistician did a recalculation of the sample size based on actual HIV incidence rates to date. The overall annual incidence was found to be 3.5%. In order to maintain 90% power to detect a 33% reduction in HIV infection, it was estimated that 500 more women were needed in the trial, bringing the total sample size to 5000 women. Each site increased their target enrollments: Harare to 2500, Durban to 1500, and Johannesburg to 1000. In addition, re-calculations of the estimated number of infections and woman-years of data needed to detect a difference between study arms have produced higher figures. Thus while previous versions of the MIRA Technical protocol listed 280 incident infections needed, and 3500 woman-years of data per arm, we are now operating under the estimates of 293 incident cases and 4000 woman-years per arm.

The final enrollment numbers for the study sites were 2502 at UZ-UCSF, 1515 at MRC, and 1028 at PHRU. A total of 5045 women were enrolled in the study.

### Assumptions Underlying Sample Size Estimates:

### 7.1.3.a HIV Incidence of 4% Per Year

All estimates we have obtained are from settings that offer state-of-the-art HIV prevention services, such as HIV counseling and testing, condom distribution, and STI treatment, similar to what we offer in our trial. To ensure we can detect an effect, we have chosen a conservative strategy in estimating an HIV incidence of 4% in the non-diaphragm control arm, which is representative of the general population. Although realistically, incidence will vary somewhat by site (Table 4), 4% is a reasonable, overall estimate.

In South Africa for example, it is likely that the incidence is somewhat higher. This is based on an estimate of current HIV incidence data from Population Council ongoing Phase II trial in South Africa. An incidence of up to 5% in the first year of the trial may be more realistic here. However, this was a 12-month study and HIV incidence in our population may decline with a longer 24-months follow-up period.

In one of our cohort studies currently ongoing in Zimbabwe, we observed a yearly incidence rate of 3.74% among women who received quarterly a full package of HIV preventive services. In another condom promotion and counseling study, a yearly incidence of 4.8 % has been observed. Both are robust estimates observed after study of more than 3000 women over three years. Each estimate was derived from a target population identical to that proposed here. Thus, an average incidence rate of 4% seems realistic.

Together, estimates from the three sites indicate that an expected incidence rate of 4% is a reasonable overall average.

### 7.1.3.b Effectiveness of 33%

This Phase III study is powered to detect effectiveness (biological efficacy combined with adherence) of 33 percent. In Microbicide Efficacy Trial Design Meetings convened in January 1998 and August 2000, comprehensive groups of scientists considered key methodological issues and agreed at both meetings that an effect size of 33 percent was justifiable. This effect size also is consistent with the current recommendations of the International Working Group on Microbicides. The HIV Prevention Trials Network (HPTN) also uses 33% as their effect size. Additionally, this is consistent with the figure employed in most HIV vaccine trials. Reduction in risk of one-third would have an enormous impact on the epidemic. Although it necessitates a larger sample size, a trial capable of detecting a smaller effect size lowers the chances that a life-saving technology will be discarded for failing to clear an unreasonably high hurdle.

### 7.1.3.c Two-tailed versus One-tailed

Despite the fact that the diaphragm is unlike some candidate microbicides whose mechanism of action is to break down cell walls of both HIV and the cervical epithelium, the possibility remains that diaphragm use may be associated with increased infection risk via unknown mechanisms. For this reason, we felt that a two-tailed test would be most appropriate.

### 7.1.3.d Confidence of 95% (α=0.05) and Power of 90%

If HIV incidence is higher than we estimated, we may achieve higher than expected statistical power, or may be able to stop the study early. A DSMB will review interim data after one-third of expected HIV seroconversions—approximately 100 events—have occurred.

Each study site has established participant retention procedures to achieve loss-to-follow-up rates that do not exceed the HIV infection rate among local study participants to minimize potential bias associated with loss-to-follow-up. However, the assumed overall retention rate of 80% was factored into the sample size calculations to adjust for the increase in variability associated with these rates.

## Secondary Endpoints

Although the primary endpoint of this trial is HIV infection, we are studying other sexually transmitted pathogens as secondary endpoints. Evidence from a recently completed Phase II Population Council microbicide trial in South Africa suggests that the incidence of *Chlamydia trachomatis* will be higher than the incidence of HIV and the incidence of *Neisseria gonorrhoeae* will be similar to the incidence of HIV. In our current studies in Zimbabwe, incidence rates for *Chlamydia trachomatis* and *Neisseria gonorrhoeae* mirror those we observed for HIV and is higher for *Trichomonas vaginalis*. A very high incidence rate for HSV-2 is expected as well. Thus, our sample size will be adequate to also assess the effect of the latex diaphragm used with lubricant gel against these pathogens. Additionally, we will evaluate them as potential effect modifiers of a diaphragm-HIV relationship.

# 8 Study Products

## Composition of Study Products

**Diaphragm**

The All-Flex® Arcing Spring diaphragm (Ortho-McNeil Pharmaceutical, Inc.) is a molded, buff-colored, natural rubber vaginal diaphragm containing a distortion-free, dual spring-within-a-spring that provides unique arcing action.

**Lubricant Gel**

Replens® (Lil’ Drug Store Products, Inc.) is an over-the-counter vaginal moisturizer which has been used as a placebo in recent microbicide safety and effectiveness trials.^27,28,29^ Ingredients include purified water, glycerin, mineral oil, polycarbophil, carbomer 934P, hydrogenated palm oil glyceride, sorbic acid, methylparaben, and sodium hydroxide. Replens® is not a known contraceptive.

## Randomization

Ibis Reproductive Health produced a randomization scheme to assign women to one of the two study groups based on a permuted block randomization design based on randomly assigned block sizes of 8, 10 and 12. The randomization scheme is stratified by study site. When each woman enrolls, field staff open the next sealed, opaque, numbered envelope at that site to reveal the study group to which the woman will be assigned. Staff do not open the envelopes until a woman is ready to be enrolled to avoid manipulation of the randomization scheme. Each woman’s group and randomization number are be noted on relevant study forms and in the study register.

## Product Administration Plan

At enrollment, women randomized to the diaphragm group are fitted for diaphragm size and receive an Ortho All-Flex® diaphragm. Staff also instruct women about how to use the Replens® gel and how to properly clean and store their diaphragms and gel applicators***.***

To improve diaphragm compliance during the trial, only women who are able to correctly insert the diaphragm are enrolled into the study. In order to avoid introducing a bias between the study arms, all women practice diaphragm insertion *before* they are randomized. Women who are not able to correctly insert/remove the diaphragm after five attempts are not eligible to enroll into the study. Following randomization, women in the diaphragm arm will receive condoms, a diaphragm and gel, and more counseling. Women in the condoms-only arm receive condoms and counseling and are told that they can receive a diaphragm to keep and use as they wish at the end of their participation in the study.

Throughout the study, we encourage women to return to the study site if they experience any problems using the diaphragm or would like further instructions or advice. We also ask women to return to the clinic or mobile site at any time during the study if they feel discomfort. A clinician provides a larger- or smaller-sized device as needed. Women who have a vaginal delivery during the study are re-fitted for diaphragm size. As part of ongoing quality assurance processes, there are systematic checks of diaphragm fittings, which sometimes result in a woman being fitted with a new diaphragm size. Replacements for damaged or lost diaphragms can be obtained in the clinic at any time during the study or can be distributed through home visits when compatible with confidentiality requirements.

Women are instructed to insert the diaphragm at any time before sex. We ask them to put lubricant gel in the dome and rim of the diaphragm at the time of insertion and to reapply gel in the vagina before each act of vaginal sex. We ask them not to cleanse the vagina or the diaphragm immediately after sex but to leave the device in place for at least 6 hours. Staff also instruct women about how to properly clean and store their diaphragm and gel applicator. Women are allowed to resume normal vaginal hygiene after sexual intercourse (*i.e.* cleanse outer genitalia with water or water and soap) as long as they do not remove the diaphragm within six hours after sex.

As stated earlier, women in both study arms receive safer-sex counseling and are offered free male condoms. Condoms dispensed by the study are unlubricated or have a non-spermicidal lubricant. The number of condoms women receive is based on expressed need, and if a participant runs short of condoms, she can come to the study clinic at any time to obtain more. While condom use will be promoted throughout the trial, women who indicate that they will not use condoms will not be required to take condoms home with them.

At closing, all women in both arms of the study are eligible to receive a diaphragm, plus a supply of Replens gel to last through the end of the study (May 2007).

## Product Supply and Accountability

Once received at the study site, all supplies will be stored in a secure, limited-access area. Clinic staff will account for all supplies received and dispensed, and at the end of the study, may then destroy or otherwise dispose of surplus supplies as agreed upon with UCSF.

UCSF will outline the product-related responsibilities of each site, including procedures for adequate and safe receipt, handling, storage, and dispensing of diaphragms, lubricant gel and condoms. This includes product accountability records that must be kept to document supplies received and dispensed to study participants.

## Compliance Counseling and Assessment

Compliance counseling is provided to study participants upon enrollment and at every visit thereafter to help ensure high rates of product use. Counseling is provided in accordance with a standard manual that addresses such topics as client-centered strategies to remember to use study products for each episode of vaginal intercourse, ensure the availability of the products both in the home and away from home, and to negotiate product use with “primary” and “non-primary”’ partners. For participants who have adherence problems, every effort is made to identify adherence strategies to increase their rates of product use throughout the course of the study. We record diaphragm, gel and condom use at each quarterly follow-up visit.

In an effort to improve validity of self-reported sexual behavior and compliance data, we use Audio Computer Assisted Self Interviews (ACASI). ACASI has been shown to increase reporting of sensitive behaviors due to perceived increased confidentiality and privacy and has been found feasible in developing country settings. The Population Council tested ACASI at two South African trial sites during a Phase II microbicide trial and found that it was feasible and highly acceptable to trial participants, including those of low literacy.^30^ Similarly, in Zimbabwe in a recent study where women of reproductive age were randomized into an ACASI or face-to-face interview about reproductive health issues, the majority of women in all educational groups said that it was easy to understand the recorded questions, that they were very comfortable entering their responses into the computer, and that they preferred ACASI to in-person interviews, especially when answering questions about sexual behavior.

Face-to-face interviews provide our main data on demographics, medical history, acceptability, and clinical assessments. The ACASI program includes questions that are particularly sensitive, such as questions about diaphragm and gel use, condom use, and other sexual behaviors such as numbers of partners and anal sex. Experience has shown that diary data do not provide accurate estimates of use in this type of study population.^29^ Using ACASI, we ask women whether they use study products “every time,” “more than half the time,” “less than half the time,” or “Never” to be able to distinguish measures of effect among perfect users, never users, and estimates of a dose response for users in-between. Together with our TAC, we have been monitoring compliance rates over time and adherence-counseling methods have been updated to address lower-than expected rates.

# 9 Study Procedures

## Scheduled Visit Summary

There are seven to eleven scheduled visits over one to two years depending on the participant’s time of entry into the study including:

- Screening visit
- Enrollment visit
- Product and safer sex counseling visit at 2 weeks post-enrollment
- 4-8 quarterly visits post-enrollment through months 12 to 24 (closing visit)[[1]](#footnote-2)

Unscheduled interim and post-trial visits take place when desired by a participant or by the study staff to follow-up on adverse events (AEs), to provide test results, for re-supply of study products, or for other reasons associated with trial participation.

We will conduct exit focus group discussions and in-depth interviews with a sub-sample of participants and their male partners after women complete the trial to further assess the acceptability of diaphragm and gel use. Volunteers will go through a separate consent process before participating in the focus group discussions. Specific procedures to measure acceptability will be developed.

## Recruitment and First Contact

Study sites recruit women from family planning and general health clinics, through community-based organizations, through word-of-mouth in the community, by postering and leafleting in strategic areas, and by advertising in the general media if necessary. These methods have proven successful in existing studies in each site.

Study staff approach potentially eligible women at recruitment sites and ask them if they are interested in learning more about a diaphragm trial in which they may be able to participate. Recruitment and educational materials are used to convey the key aspects of the trial to potential participants, such as study objectives, inclusion and exclusion criteria, study procedures including random assignment to a study arm, diaphragm and condom use, enrollment exam and laboratory testing, the visit schedule, and study risks and benefits. All materials used for recruitment and education of potential participants are reviewed by the UCSF Committee on Human Research and by other relevant local and US-based IRBs.

If a potentially eligible woman expresses interest in trial participation, she is given an appointment for screening at one of the study clinics.

## Screening Visit

Screening is a step-wise process, beginning with questioning about inclusion and exclusion criteria using a standardized eligibility checklist and ending with laboratory and clinical assessments. These laboratory and clinical procedures only take place after the participant has given written informed consent for screening. Study staff obtain oral consent from the potential participant before administering the checklist, maintain privacy and confidentiality, and do not document the participant’s name or any other personal identifiers on the checklist.

A study staff member reviews the Screening Informed Consent Form with the potential participant and provides further information as needed. If the potential participant wants to join the study and the staff member feels confident that the woman understands they both sign the Screening Informed Consent Form. If the participant is illiterate, either her representative or a witness who is not a member of the study staff may sign the consent form in her place. Alternatively, a thumbprint together with the signature of the study staff member who gave the information about the study and obtained consent can be used.

The following procedures take place:

- Oral consent to complete eligibility checklist obtained.
- Eligibility Checklist completed. Women who are not eligible based on the checklist complete their study participation at this point.
- Written screening informed consent.
- Short questionnaire on demographics and condom use.
- Pre-test counseling for HIV and other STIs.
- Urine specimen for *Chlamydia trachomatis*, *Neisseria gonorrhoeae*, and *Trichomonas vaginalis* testing.
- Fingerstick (or venipuncture) for blood sample to do OraQuick®[[2]](#footnote-3) and Determine^®c^ rapid HIV testing.
- HIV results notification.
- Post-test counseling for both HIV-positive and HIV-negative women.
- Women who have inconsistent HIV rapid testing results (one positive and one negative result) at screening will have a venipuncture to collect a blood sample for confirmatory laboratory ELISA^e^ testing.
- Scheduling of results/enrollment visit within two weeks post-screening.
- Collect locator information.

All procedures are performed by qualified, trained study staff. Staff are free to provide additional services to study participants or to refer them to appropriate services for any reproductive health complaint.

Women can refer their male partners for testing and counseling for HIV and other STIs at any point during the study. Male partners of women identified with laboratory-confirmed *Neisseria gonorrhoeae*, *Chlamydia trachomatis*, or *Trichomonas vaginalis* are notified according to national partner notification guidelines. Care and support for women who test positive for HIV at screening or during the trial is outlined in Section 9.10.

UCSF, the laboratory monitor, and the medical advisor on this protocol developed clinical procedures that include instructions on urine collection, OraQuick® and Determine® rapid HIV testing, PCR and other STI diagnostic tests, and performing pelvic exams. They also developed guidelines for the treatment of STIs and vaginal infections, clinical and psychosocial referrals, clinical management of safety endpoints and AEs, universal precautions, and quality control of clinical procedures. Each site modified these procedures and guidelines according to local circumstances. In addition, each site developed a site-specific accidental exposure to blood or body fluids procedure and a partner notification procedure.

We provide all participants with the name and telephone number of the local principal investigator and a study staff member who speaks their language in case they have any questions about the study. In the event that a participant has questions or concerns about her rights as a study participant and would like to discuss them with a non-study staff member, all participants are given the name and telephone number of a member of the local IRB as an independent source of information.

## Enrollment Visit

For the majority of eligible women (*i.e.* those who do not need confirmatory HIV testing) the enrollment visit takes place within two weeks after the screening visit. Women who have inconsistent HIV tests at screening and require confirmatory testing also return to the clinic to receive their HIV results. Those with negative confirmation results may then enroll provided they return to the clinic within two weeks.

Women who return to the clinic for their enrollment visit greater than two weeks but within 30 days of their screening visit have repeat HIV testing. If HIV-negative and otherwise eligible, they can be enrolled into the trial.

For women who do not enroll within 30 days of their screening visit, repeat screening procedures including HIV and STI testing are completed and they are asked to return to the clinic within two weeks for enrollment. We give participants the results of the laboratory tests performed at the screening visit at their enrollment visit*.*

The following additional procedures take place at the enrollment visit:

- STI test results notification. Women who needed confirmatory HIV testing due to inconsistent results at screening are given their results.
- Post-test counseling.
- Treatment of women who tested positive for CT / NG / TV at screening visit and, when compatible with confidentiality requirements, provision of referral testing and treatment services to their partners.
- Review of eligibility checklist to confirm eligibility.
- Review of screening questionnaire.
- Written informed consent for main study.
- Urine specimen for pregnancy testing. Urine sample is stored for future testing. Pregnant women complete their study participation at this point.
- UTI diagnosis. Urine specimen for culture is only collected if a woman is symptomatic and has a positive dipstick urinalysis. Women who are treated for UTIs may still be enrolled.
- ACASI baseline questionnaire.
- Pelvic exam with speculum and inspection of vagina and cervix solely to determine presence of a healthy cervix for eligibility.
- Assessment of any signs noted by clinician during pelvic exam or symptoms self-reported by participant of STIs and reproductive and urinary tract infections. If women are diagnosed with a current STI/RTI and it is treatable at the visit, they can be administered treatment and continue with the enrollment process.
- Pap smears are provided as an optional service for women.
- Following fitting procedures, the clinician fits the participant with as many sizes as deemed necessary before determining a final diaphragm size.
- Instructions on use, including insertion and removal techniques are provided to the participant. A study clinician supervises diaphragm insertion and removal. Only women who are able to insert and remove the diaphragm are eligible. Women are provided with education and given five attempts to correctly insert and remove the diaphragm. The clinician collects all diaphragms after removal.
- Venipuncture for blood sample is used for TP and HSV-2 testing. Blood samples from all women are stored for future testing.
- Blood of HIV-negative women is used for later PCR testing of timing of infection for those participants who test positive for HIV at the month 3 visit.
- Randomization to one of the two study groups.
- Distribution of Ortho All-Flex® diaphragms and Replens® gel to all applicable women, with instructions on use, including insertion and removal techniques.
- Safer-sex counseling and distribution of male condoms to all women.
- Instructions on available resources for minor or moderate medical problems or emergencies.
- Administration of the Informed Consent Quiz form that assesses a participant’s understanding of the informed consent process and retention of that information.
- Scheduling of two-week visit.
- Update of locator information.

We attempt to minimize social desirability bias throughout the study by interviewing participants about sexual behaviors before counseling them about safer sex and/or by having different staff members interview and counsel each woman at each visit, depending on preferences and feasibility at each study site.

**9.4a Two-week Follow-up Visit**

All women return to the clinic two weeks after enrollment to receive any laboratory results and for product and safer sex counseling. The procedures are as follows for women in both groups:

- Notification of laboratory results and treatment for positive results.
- Post-test counseling.
- Short questionnaire on study product use.
- Safer-sex counseling and distribution of male condoms to all women.
- Brief diaphragm counseling for applicable women, including assessment and problem-solving of difficulties with using the diaphragm, re-supply of gel and replacement diaphragm if damaged or lost to participants in the diaphragm group.
- Scheduling of 3-month follow-up visit.
- Update locator information.

## Quarterly Follow-up Visits

After enrollment, women return quarterly for the duration of their participation in the trial (at the end of months 3, 6, 9, 12, 15, 18, 21 and 24). The visit schedule and procedures will be identical for women in both study arms.

At each follow-up visit, participants undergo the following procedures:

- Follow-up interview—short interview about compliance and sexual behavior at every visit, longer interview including acceptability assessment during the month 3 and month 24 visits or closing visit if it is before 24 months.
- Urine specimen for pregnancy, CT, NG, and TV testing; urine sample is stored for future testing.
- UTI diagnosis: urine specimen for culture is only collected for laboratory testing if a woman is symptomatic and has a positive dipstick urinalysis. Women who are treated for UTIs may continue to use the study products.
- HIV and STI pre-test counseling.
- Venipuncture for blood sample to be used for HIV, HSV-2, and TP testing (TP testing only done at closing visit). Blood samples from all women will be stored for future testing.
- Blood of HIV-positive women (seroconverters) will be used for later PCR testing to determine timing of HIV infection.
- Women who were HSV-2 negative at enrollment are tested again at month 24 visit or closing visit if it is before 24 months. Stored blood samples collected at quarterly visits from women who are HSV-2 positive at closing are tested to determine timing of HSV-2 infection.
- OraQuick® and Determine® rapid HIV testing.
- HIV results notification.
- HIV and STI post-test counseling for both HIV-positive and HIV-negative women. Samples from women who have positive or inconsistent HIV results undergo confirmatory laboratory ELISA testing.^f^
- Reminder to participants to return to the study clinic if they experience discomfort, abnormal discharge or other symptoms, or to the nearest clinic/hospital in case of a medical emergency.
- Safer sex counseling and distribution of male condoms to all women who accept them. Brief diaphragm counseling including assessment and problem-solving of difficulties with using the diaphragm, re-supply of gel and replacement diaphragm if damaged or lost to participants in the diaphragm group.
- Administration of Informed Consent Quiz form at 12-month visit or closing visit if it is before 12 months.
- Scheduling of next follow-up visit.
- Update locator information. Exiting participants will be asked for updated contact information and to sign a form indicating their permission or not to contact them after their participation in the trial is completed in order to inform them about trial results, inquire about study product use experience, and/or to participate in future trials.

Women only receive a pelvic exam when clinically indicated. Instructions for complete documentation of AEs and SAEs appear in Section 11. If a women tests positive for HIV or STIs, or if any other abnormalities are found, we provide treatment as outlined in Sections 9.8 and 9.10.

Women may participate and be followed in the study for up to 24 months.

## Unscheduled Visits

We encourage participants to return to the clinic at any point if they feel there is a problem or if they need additional supplies. If participants report for unscheduled visits, an evaluation of the problem is undertaken and any necessary treatment provided. Findings are recorded on the appropriate study forms.

The following procedures take place at unscheduled visits:

- Notation of last use of diaphragm, gel and condoms.
- Notation of reason for interim visit:
- If for AE or SAE or re-evaluation of previous AE or SAE, appropriate physical exam procedures are conducted as necessary or participant is referred for appropriate medical follow-up. Each AE is documented on an Adverse Event Form and each SAE on a Serious Adverse Event Form (see Section 11).
- If for interim HIV counseling and testing in response to presumed exposure to HIV and/or symptoms of acute infection, counseling and testing are provided as needed.
- If to receive results of laboratory tests, results and treatment are provided as appropriate.
- If to obtain a replacement diaphragm or more gel or male condoms, supplies are provided as needed and recorded on product accountability records.
- If to ask questions or discuss problems with study protocol, guidance is provided as needed.
- Remaining visit schedule is confirmed and changes made as needed
- Locator information is updated

## Contraceptive Use and Pregnancy

All women receive contraceptive counseling at enrollment into the study, including discussion of the relative contraceptive efficacy of condoms and diaphragms used without spermicide. If the MIRA study clinic is unable to provide contraception, referrals for contraception and other family planning services are given to those who desire it.

We continue to follow up pregnant participants. Those in the diaphragm and lubricant gel group are not required to discontinue diaphragm or gel use, though they may chose not to use their device or gel for the duration of the pregnancy.

## STI and RTI Diagnosis and Treatment

As described in Sections 9.3 and 9.4, we test all women who are eligible for the trial and choose to participate for HIV and other STIs. Women who are laboratory positive for *Chlamydia trachomatis* and/or *Neisseria gonorrhoeae* complete treatment before they are enrolled. Women who have a diagnosed or suspected current STI or reproductive tract infection at enrollment including laboratory diagnosed *Trichomonas vaginalis* and/or *Treponema pallidum* are provided treatment and enrolled. Throughout the study, we collect specimens for STI testing at each follow-up visit. We contact and inform infected women of their results. We provide them with STI treatment in accordance with WHO guidelines free of charge and offer STI testing and treatment for their partners. We pay special attention to possible urinary tract infections (UTIs) because of the association between such infections and diaphragm use.^31,32^ The study pays for the treatment of incident UTIs diagnosed during the study period.

For other medical conditions identified during study screening and/or follow-up procedures, we refer participants to other sources of care available in their communities. In certain cases of infections or epithelial lesions, the study clinician may decide to interrupt diaphragm and gel use.

## Cohort Retention

Once a participant enrolls in this study, the study site makes every effort to retain her in follow-up to minimize possible bias associated with loss-to-follow-up. Each site has established participant retention procedures to strive for loss-to-follow-up rates that do not exceed the incidence rate of the primary study endpoint. As such, an average annual retention rate of 93% including drop-out and true loss-to-follow-up is targeted across sites. Study site staff are responsible for developing and implementing local procedures to achieve this goal. Components of such procedures include:

- Thorough explanation of the study visit schedule and procedural requirements during the informed consent process, and re-emphasis at each study visit.
- Thorough explanation of the importance of both study treatment groups to the overall success of the study.
- Collection of detailed locator information at the study Screening Visit and active review and updating of this information at each subsequent visit; locator information may be verified for follow-up purposes.
- Use of mapping techniques to establish the location of participant residences and other locator venues.
- Use of appropriate and timely visit reminder mechanisms.
- Immediate and multifaceted follow-up on missed visits. If a participant is more than one week late for a scheduled visit, attempts are made to locate the participant. If a participant returns to the clinic for a quarterly follow-up visit more than 5.5 months after the scheduled visit date, the visit is considered a missed visit and the next quarterly visit is completed instead.
- The use of trained outreach workers to trace participants and visit them regularly at their homes and/or other community locations when compatible with confidentiality requirements. This is standard procedure even in Zimbabwe where we usually see participants in the clinic. Missed visits are followed by tracing in the community and a possible home visit.
- Regular communication with the study community at large to increase awareness of HIV/AIDS and explain the purpose of HIV prevention research and the importance of completing research study visits.

We generate monthly reports on the number and percentage of participants completing the quarterly follow-up visits at which STI and HIV testing are performed to assess study endpoints. The Protocol Director, study coordinators, and monitors track retention rates closely and work with study sites as needed to take any required action to address below-target retention.

Every time a study staff member attempts contact with a study participant, including letters and phone calls, this is documented in the participant’s study file. If the participant cannot be located, her file remains open and attempts to locate her continue until study close-out. Participants cannot be deemed lost to follow-up until the study has closed. If the participant is located or returns to the clinic on her own but indicates she is no longer interested in study participation, the closing form indicates that the participant withdrew consent and the reason why. If the study closes, the participant is not located, or the participant does not return to the clinic before the study closes, the closing form indicates that the participant was lost to follow-up.

## Care and Support to Study Participants

The following services are provided to all women in the trial:

- Safer-sex counseling and free male condoms.
- Referral of male partner(s) to a voluntary HIV counseling and testing site or provision of VCT services at the trial site. The trial does not pay for referrals for partner(s) of women screened, but pays for referrals of male partner(s) of women who are enrolled in the trial.
- Screening and treatment for STIs at the study clinic.
- Treatment of *Chlamydia trachomatis*, *Neisseria gonorrhoeae*, and *Trichomonas vaginalis* for male partner(s) at the study clinic as needed.
- Treatment of incident UTIs diagnosed during the study period**.**
- Pap smears are provided as an optional service for women. Referrals are provided for those women who need clinical follow-up for abnormal results.
- Provision of selected methods of contraception at the study clinic or at a nearby public sector family planning clinic (contraception is free/subsidized in the public sector in both South Africa and Zimbabwe).

Each site developed a care and support strategy for women who test positive for HIV at screening and for women who seroconvert during the trial. Study staff provide participants with their HIV test results in the context of post-test counseling. They also refer persons found to be HIV-infected to available resources for medical and psychosocial care and support as well as to any available research studies for HIV-infected persons. For any participants found to be HIV-infected who also become pregnant during follow-up, every effort is made to facilitate access to single-dose nevirapine and/or other interventions to reduce the probability of HIV transmission to the participant’s infant.

We offer women who test HIV-positive at screening the following services, but do not pay for any referral care resulting from clinician contact at screening:

- Supportive counseling, counseling about healthy living, and other psychosocial care at study clinic and/or by referral. Referral to support group(s).
- Referral for tuberculosis screening, and if applicable, prophylaxis as per local guidelines.
- Referral for clinical staging of HIV disease and/or laboratory tests to determine immune status (full blood count, white cell count, or CD4 count) if available.
- Referral for treatment of opportunistic infections and common morbidity.
- Referral for other prophylaxis (*e.g*. influenza vaccine, co-trimoxazole) as per local guidelines.
- Referral for antiretroviral treatment, if applicable and available, as per local guidelines.
- Referral to HIV treatment trials, if applicable and available.
- Referral to prevention of mother-to-child transmission programs, if applicable and available.

Women who seroconvert during the trial receive the following care and support throughout the trial:

- Services described above. In addition, services can be performed at the study clinic or referrals paid for with trial funds. Participants are monitored as appropriate throughout the trial. Please see the “Managing Seroconverters in the MIRA Trial” protocol in the Appendix section for a complete description of services provided to trial seroconverters.

HIV-positive women who choose to enroll in an HIV treatment trial are discontinued from the study. Seroconverters who do not enroll in an HIV treatment trial may continue to participate and are be followed for ascertainment of secondary endpoints.

## Recontacting Participants after Exiting the Trial

If positive test results are received after a participant’s closing visit, she will be recontacted for treatment and counseling. Otherwise, women will not be recontacted after exiting the trial unless they give their express permission for staff to do so using the “Permission to Contact” form and indicate the manner(s) of contact that are acceptable to them. Study site PIs will decide whether to disseminate trial results to participants by contacting all those who indicated that they wished to be contacted with this information or by holding community meetings. Information about post-trial evaluative studies and new research trials will be shared with women who expressed an interest in participating in future research.

## Use of Other Products and Medications

### Condoms

Study staff provide all potential participants with intensive counseling to promote safer sex practices, including male condom use, at all study visits. Condoms that are unlubricated or lubricated with a non-spermicidal lubricant are provided free of charge to all interested participants regardless of the trial arm. Women who indicate that they will not use condoms and are not interested in receiving them are not be required to take any. Polyurethane condoms are available to participants who indicate that their partner is allergic to latex. A small number of “novelty” (*i.e.* flavored) condoms are distributed to women in both arms of the study.

### Other Vaginal Products

Women in the three trial communities may be accustomed to a variety of vaginal practices, such as vaginal douching/cleansing, and the use of traditional vaginal substances to dry and tighten the vagina. Study participants are discouraged from continuing these practices. However, those who choose to continue are encouraged to share that information with investigators. They are also told not to clean the vagina or insert other vaginal products after they have inserted the diaphragm and before sex takes place. We also tell women that they should not use any vaginal products that have nonoxynol-9 or other spermicidal compounds in them, such as spermicides and spermicidally-lubricated condoms. We encourage participants to report their vaginal practices and document them at follow-up visits. Women are allowed to resume usual vaginal hygiene after sex, such as washing outer-genitalia with water or water and soap, as long as they do not remove the diaphragm within six hours after sex.

### Concomitant Medications, Including Contraceptives

The use of concomitant medication, including western medicines and contraception, traditional medicines, and vitamins, is recorded on the concomitant medications log. Concurrent use of all such medications is permitted under the protocol.

## Focus Group Discussions and In-depth Interviews

Subgroups of participants and their male partners are asked to participate in focus group discussions or in-depth interviews about aspects of diaphragm acceptability after completion of their participation in the trial. Their purpose is to complement the acceptability and compliance questionnaire data, which is collected throughout the trial. A separate protocol has been developed for these portions of the study (see Appendix 16.3).

# 10 Specimen Evaluation

## Specimen Tracking and Storage

Two types of laboratory testing are conducted throughout the trial:

- On-site testing: pregnancy testing, dipstick urinalysis, rapid HIV testing using OraQuick® and Determine®, and wet mounts when clinically indicated.
- Testing at local laboratories: NG, CT, and TV PCR, HSV-2 and HIV ELISA tests, HIV PCR confirmatory tests, culture for symptomatic UTIs, and TP RPR and TPHA tests.

Specimens are stored in the appropriate freezers (-20°C or below) or slide boxes dedicated to trial specimens. All specimens are clearly labeled with the participant’s ID number, study site, and the specimen collection date. Any personal identifiers that link this ID to the participant are kept in a separate location under lock and key or secured within a password-protected computer. Only co-investigators and their designees have access to any personal identifying information that could link the participant to the ID number.

Urine and blood samples will be taken from consenting participants for long-term storage and will be labeled as described above. Each trial site will develop a specimen-tracking database and specimen-tracking forms. Specimens will be maintained by employees at each site’s laboratory facility. They are strictly for use by co-investigators of the study, and will not be made available to other investigators. Samples will be used for further STI testing or testing for other pathogens. Specimens will never be used to develop commercial products.

These urine and blood specimens will be kept for up to ten years, at which point they will be disposed of by site personnel. Participants may withdraw their consent to store specimens or request that the specimens be destroyed at any time. Specimens from participants who do not consent to long-term storage will be destroyed when the study is completed and all data is considered “clean.” *No specimens should be destroyed without first obtaining written permission from UCSF.*

## Specimen Collection and Testing

The following specimens are taken at all or some study visits:

- Urine sample for on-site hCG pregnancy testing.
- Urine for TV, NG, and CT testing. Urine samples are stored for future testing.
- Urine specimen for culture is only collected for laboratory testing if a woman is symptomatic for a UTI and has a positive dipstick urinalysis (result of +1 or greater for blood, leukocyte esterase, protein, or nitrites).
- Vaginal fluid sample for wet mount when clinically indicated.
- Blood specimens for HIV, HSV-2, and TP testing are collected via venipuncture and blood samples for each participant are stored for future testing. Blood from HIV-negative women is used for later PCR testing for timing of infection.
- On-site rapid HIV testing (Oraquick® and Determine®). Samples from women who have inconsistent HIV rapid test results or two rapid tests, incidating a possible HIV-seroconversion will undergo confirmatory laboratory Enzyme linked immuno-sorbent assay (ELISA) testing.

Clinic staff conduct rapid on-site pregnancy and HIV tests according to test kit instructions. The same HIV test kits are used at all study sites. Findings are recorded on the relevant clinical and lab results forms.

*Chlamydia trachomatis* and *Neisseria gonorrhoeae* PCR tests (Roche) and HIV DNA PCR tests (Roche) are conducted by local laboratories according to the test kit manufacturer’s instructions. *Trichomonas vaginalis* PCR tests are conducted by local laboratories according to guidelines provided by the study Laboratory Consultant. Results of all such tests are available within 14 days from sample collection. Results are reported on laboratory-specific forms and transcribed onto the Lab Results Form. In the event of inconsistent HIV rapid tests or two positive rapid tests (indicating a potential seroconversion), HIV ELISA is performed on serum to confirm HIV serostatus. HIV DNA PCR and in certain circumstances, HIV RNA PCR, tests (Roche) are used to confirm HIV serostatus at enrollment.

All women are tested for HSV-2 at enrollment. Women who test negative or indeterminate at enrollment are retested for HSV-2 at Month 24 visit or closing visit if it is before 24 months. Stored blood samples of those women who tested positive at closing will be tested to determine the timing of HSV-2 infection. Local laboratories conduct all HSV-2 ELISA tests (Focus Technologies, Inc.) according to the test kit manufacturer’s instructions.

Women enrolled into the study are tested for *Treponema pallidum* at the enrollment and closing visits. RPR positive samples undergo TPHA testing. All testing is completed according to manufacturer’s instructions.

## Standardization and Quality Control

Because on-site tests are conducted in several different study clinics and local lab testing in several local labs, special attention is paid to standardization and QC across clinics and labs. All on-site, local laboratory technicians were trained at the inception of the study and a MIRA-appointed laboratory consultant monitors central laboratory and site laboratory procedures at each site, once per year.

Each local laboratory adheres to standards of Good Laboratory Practice (GLP), has external QC procedures, and documentation of these procedures in place. Furthermore, they use the internal controls provided by the manufacturers of automated tests and test kits.

Monitors for this trial (see Section 14.3) review laboratory source documents and Lab Results Forms for all tests performed as part of the study protocol. This includes review of results from different types of HIV tests to verify consistency. Special emphasis is placed on minimizing transcription errors of HIV test results to avoid reporting incorrect test results to study participants.

# 11 Adverse Event Guidelines

## 11.1 Adverse Events and Serious Adverse Events

### 11.1.1 Definitions

The following definitions apply to adverse events occurring in clinical studies involving drugs:

- An adverse event (AE) is any health-related reaction, effect, toxicity or abnormal laboratory result that a participant experiences during the course of the study, irrespective of relationship to study product use.
- A serious adverse event (SAE) is defined as any untoward medical occurrence that:
- Is life threatening or results in death
- Requires in-patient hospitalization or prolongs existing hospitalization
- Results in persistent or significant disability/incapacity
- Is a congenital abnormality/birth defect
- Jeopardizes participant and required medical/surgical intervention to prevent serious outcome, *or*
- Any other event that the investigator considered serious.

### 11.1.2 Adverse Event and Serious Adverse Event Reporting

AEs are to be reported only for untoward medical events that occur in participants after they have enrolled in the trial. An event that occurs in a participant between her screening and enrollment visits is recorded, but is not considered an AE. Only new untoward events or a worsening of an existing condition are considered AEs. Conditions that were present before enrollment into the trial and that remain stable or improve during the trial are not considered AEs. Untoward events that seem clearly unrelated to the study product, such as broken bones or lacerations sustained during car accidents, are also reported as AEs or SAEs.

In this study, the following routine trial measurements are not be considered AEs because they are defined as trial endpoints:

- Any laboratory-diagnosed incident HIV, CT, NG, TP, HSV or TV infection, regardless of whether it is diagnosed at a scheduled or unscheduled study visit. These infections are routinely captured on Lab Results Forms. However, they are reported as SAEs if they result in hospitalization or another unexpected complication. Symptoms of any laboratory-diagnosed incident HIV, CT, NG, TP, HSV, or TV infections are not recorded as adverse events.
- A positive pregnancy, regardless of whether it is diagnosed at a scheduled or unscheduled study visit, at another facility, or by the participant herself. These results are routinely captured on Lab Results Forms. Though pregnancy is not considered an AE, pregnancy-related conditions, such as nausea and vomiting, are considered AEs. Similarly, “normal” childbirths, including vaginal births and elective caesareans are not considered an automatic SAE or AE, but childbirth-related complications are considered S/AEs. This includes hospitalizations for emergency caesareans.
- An abnormal Pap smear, regardless of whether it is diagnosed at a scheduled or unscheduled visit. These results are captured on Lab Results Forms. However, any treatment, such as colposcopy, biopsy, or loop excision, or symptoms of treatment for abnormal Pap smears is recorded as an AE or SAE.

We question the participant and conduct laboratory testing at regular intervals throughout the trial to assess the presence of study endpoints and AEs. Participants are instructed to contact the study clinician in the event of any study endpoint or AE that they want evaluated between study visits. However, in the case of SAEs and particularly life-threatening ones, participants are instructed to seek immediate emergency care at the nearest clinic or emergency room and to notify study staff as soon as possible after their medical needs have been addressed. With appropriate permission of the participant, records from all off-site medical visits related to study endpoints, AEs and SAEs are obtained and relevant data is recorded on the Case Report Forms. All participants who report study endpoints, AEs, or SAEs will be followed clinically until the condition resolves or stabilizes. A recurrence of signs, symptoms, or infections—any of which could trigger a pelvic exam or other diagnostic procedures— reported as an AE previously, but for which adequate treatment was given or evidence of resolution was documented, is considered a new AE.

Details about each AE and each SAE are recorded on an Adverse Event Form or a Serious Adverse Event Form respectively. The on-site study clinician determines the relationship of the AE/SAE to study products as not related, possibly related, probably related, or definitely related. He or she also deems the severity of the AE/SAE as mild, moderate, or severe. For each AE/SAE, the date or approximate date of onset and resolution is recorded. This may require interim visits or continued monitoring after termination of diaphragm use. Treatment is dispensed as needed and available and use of study products is interrupted or terminated as necessary(see Section 11.3). In all cases of SAEs where a relationship with a study product is probable, the use of that product is discontinued immediately.

In compliance with GCP, SAEs and associated AEs may be reported for up to 30 days after discontinuation from the study. All unresolved AEs or SAEs that are possibly, probably or definitely related to study product use at the time of a participant’s study discontinuation are followed up until resolved, or until referral or treatment is provided.

### 11.1.3. Parties Responsible for AE/SAE Reporting

Site investigators and UCSF are responsible for reporting SAEs and related unexpected AEs to all relevant local and US-based IRBs including the UCSF Committee on Human Research (CHR). The following minimum criteria are necessary to trigger expedited reporting of an AE/SAE to these institutions:

- An identifiable patient (Enrollment ID)
- A suspect medicinal product (diaphragm, lubricant gel or male condom)
- An identifiable reporting source (co-PI or designee)
- An event or outcome that can be identified as serious AND unexpected
- A reasonable suspected causal relationship with the investigational product or with study procedures

While the initial SAE expectedness and causality assessments are approved by the site investigator on the original SAE form, the trial’s medical advisor may advise changing the SAE relatedness based on his/her medical review.

Site investigators are responsible for reporting the following to UCSF immediately and to relevant local IRBs no later than ten working days after learning about the event:

- All SAEs that resulted in death or were life-threatening for which expedited reporting is required
- All other SAEs that meet the requirements for expedited reporting
- Unexpected AEs related to study product use

UCSF in turn follows all reporting guidelines of US-based IRBs and informs all other study investigators of the event within 10 working days of being notified by the study site.

## 11.2 Product Interruption and Discontinuation

Active participation in the trial, including use of the diaphragm and/or gel, may be discontinued for any of the following reasons:

- The participant withdraws her consent. Participants may withdraw from study participation at any time, for any reason, without loss of any services to which they may be entitled outside of study participation.
- The participant develops a medical condition that is incompatible with the protocol or the clinician determines it to be in the best interest of the participant to discontinue study product use.
- The participant develops an SAE that may be related to study product use.
- Study staff determine it to be in the best interest of the participant for a non-medical reason.
- UCSF, Ibis Reproductive Health, South African MRC, the University of Zimbabwe, PHRU, the Zimbabwe and South African Ministries of Health, the DSMB, or any relevant IRB or regulatory authority halts the study at one or more site(s).

Women who acquire an STI or a vaginal infection may continue to use the diaphragm and gel. In the event of significant genital ulceration, genital lesions with epithelial disruption, or severe generalized erythema, the participant may also continue to use the diaphragm and gel if she wishes and the clinician agrees. These symptoms are picked up routinely through questions about gynecological symptoms and complaints during regularly scheduled study visits. Re-evaluation visits are scheduled at the discretion of the study clinician until healing or resolution is documented. If the condition does not improve after one or more re-evaluation visits with continued diaphragm and gel use, product use may be interrupted until healing or resolution has been documented. Diaphragm and gel use may then be restarted, if doing so is considered safe by the study clinician. If the condition reappears, permanent discontinuation is considered.

The study clinician, in consultation with the local co-PI and/or another study physician, is responsible for deciding whether temporary or permanent discontinuation of diaphragm and gel use is warranted. Every effort is made to complete all protocol-specified visits and procedures for women whose diaphragm and gel use has been discontinued. Diaphragm and gel use may be restarted at any time, if doing so is considered safe by the study clinician and after consultation with a physician as needed).

Participants are asked to abstain from having sex and using the diaphragm and gel for six weeks post-birth, LEEP, or cryotherapy. Participants are asked to abstain from having sex and using the diaphragm and gel for two weeks after a cervical biopsy, spontaneous abortion, or induced abortion.

## Communicable Disease Reporting Requirements

It is the responsibility of the co-PI of each study site to report communicable diseases identified among study participants to local health authorities as required by local guidelines.

# 12 Data Collection, Management, and Analysis

## 12.1 Identification Numbers

At screening, each woman is assigned a unique identification number, which is separate from the randomization number discussed in Section 8.2. This identification number is entered into the Study Register and written on all screening forms. This identification number is retained for this one woman and used in all subsequent visits for the duration of the trial. Each study site is assigned a range of identification numbers that adequately address the maximum number of screened women.

The Study Register is kept by study staff and contains the name, randomization, and ID number of each participant as well as her contact information for any necessary tracing. This register is kept in a secure environment at the study site, separate from the data collection forms (see Section 13.2). Signed informed consent forms are also be kept locked at the study site, separate from other study forms.

## Case Report Forms

Case Report Forms (CRFs) are used to record all clinical and laboratory information obtained in this trial. As discussed in Section 8.5, all of the self-interview data are collected using ACASI. Source documents include original laboratory reports, study clinician’s notes, counseling forms, and off-site medical records. Medical records may be obtained in the case of SAEs. Completed CRFs and source documents are kept in participant folders in locked files at each study site. One folder is kept per participant. Only study staff, monitors, and auditors or their designees can access these files.

A team of trial investigators at UCSF and Ibis designs the CRFs. Study staff at each site have been trained in how to complete and make corrections to the CRFs. CRFs are completed at the time of participants’ visits in black or blue ballpoint pen. No white-out or other obscuring method is used to correct errors. All corrections to the data is made by drawing a single line through the mistake. All corrections include the date when the change is made and the initials of the person making the change. Staff filling out the CRF check the form for accuracy and completeness. The local study coordinator or designee re-checks each form for completeness and accuracy within the next few days before transmitting and filing them.

Laboratory test results can be reported on forms that are normally used by the laboratories that conduct the tests. In that case, results are transcribed from the source documents onto the relevant study forms at each site.

## 12.3 Translation of Forms

Each study site is responsible for translating the informed consent forms, educational materials, and questions that are directed to participants on relevant CRFs into local language(s). Back-translations into English are completed by a different translator to check the accuracy of the original translations. Back-translations are compared with the original English versions by trial management staff at each site to verify that translations are appropriate. Informed consent forms and educational materials in the local language are submitted to the relevant IRBs. Translations of informed consents are verified by the Project Director at the UZ-UCSF site, by the local IRB at the MRC site, and by translation certificates from a certified company at the PHRU site. Translations, back-translations, and verification documents for informed consents are kept on file at the study site and at UCSF.

## 12.4 Forms Tracking and Storage

All study forms are completed, checked, and filed at each study site and forms will kept at the sites throughout and after completion of the data collection phase for a minimum of five years. *Records may not be destroyed without written permission from UCSF.*

## 12.5 Data Entry and Management

Following each visit, all CRFs are checked at the site for accuracy and completeness. They are then transmitted by email or fax to the Center for International Data Evaluation & Analysis at UCSF where quality control and data management procedures are conducted for all three study sites.

All study participants receive a unique study identification number (ID) that is recorded on all paper forms and in the ACASI system. One electronic file that links participants’ names and study ID numbers per site is maintained by the study coordinator or their designees and is stored on a password protected, secure computer. The file associating names and ID numbers and the ACASI data files is not saved on the same computer.

Paper consent forms, contact information sheets, and other paper data collection instruments are stored in a locked file cabinet located in the project offices at the three study sites. Documents containing personal information, such as consent forms and contact information, are stored separately from study data.

Data from ACASI-based interviews will remain on the password protected computers through the duration of data collection. Daily backups of the ACASI data are done. Copies of the ACASI data are printed at the study sites on a biweekly basis. Print outs of data are signed and dated by the study coordinator and kept in a locked file cabinet. The electronic data is merged once a week and sent to the UCSF data center via email in compressed and encrypted form. ACASI data is converted into SAS (Version 8, SAS, SAS Inc., Cary, NC, USA) to check for cross-visit consistency and to compare ACASI data with associated CRF data.

Incoming CRF data is processed and reviewed using DataFax version 3.7 (Clinical DataFax Systems, Inc.), a commercial software system. This software converts the data on paper CRFs to an exportable database through automatic optical character recognition. 100% of the images of the original CRFs are compared against the database to verify the accuracy of recognized data and to enter data that cannot be recognized electronically. Data is checked for completeness and accuracy, including checks for ranges and skip patterns, by data coordinators using validation tools in DataFax and in SAS. Validated and checked data from clinic visits and ACASI is merged using SAS for additional data integrity checks, the generation of monthly reports on recruitment and follow-up rates, and analysis.

Study data at UCSF is backed up nightly. Backup schemes are based on a five-day weekly schedule. A full backup is performed once a week, with incremental backups on the other days of the week. Weekly tapes with a full backup of the data are stored at a remote location. Monthly tapes will be regularly archived with professional data storage and recovery service in Sacramento, California. Six months of data is maintained at the storage facility.

Information collected on study forms is checked five times: by staff completing the forms at the study visit, by the local study coordinator or designee, by monitors during monitoring visits (see Section 15.3), by data verifiers upon receipt of the images in DataFax, and during data cleaning by UCSF data manager or designee(s). Coding of AEs to specific body systems using the MedDRA coding system is carried out at UCSF by experienced staff that do not have participant contact.

## 12.6 DSMB analyses

The DSMB will review interim data when 100 HIV seroconversions (approximately one-third of the expected 293 infections) have been observed. Based on the advice of the DSMB, an additional interim analysis will be conducted when 195 HIV seroconversions (approximately two-thirds of the expected 293 infections) have been observed. They will tabulate events by study group, site, and woman-years of exposure.

The interim analyses will be performed using an O’Brien Fleming group sequential design. The assumptions used to determine sample size (*i.e.*, accrual, HIV seroincidence, and retention rates at each site) will be assessed and alterations to the overall sample size will be made if recommended by the DSMB. A consulting statistician will develop the DSMB data analysis plan in close collaboration with the co-PIs and data managers of the trial. However, the analyses will be conducted by the consulting statistician and his/her team without involvement of the co-PIs, data managers, any other trial investigators, or any other trial management staff.

The DSMB will also provide clear stopping rules and guidelines for other protocol modifications based on the interim results. The DSMB may also recommend other protocol changes. If interim results provide convincing evidence of safety concerns, significant benefit of diaphragm use, faster accumulation of trial endpoints than anticipated, or futility (*i.e.* a very low probability that the trial will ultimately demonstrate that the diaphragm is effective), the DSMB may recommend early termination of the trial. If interim results suggest that the incidence of trial endpoints is lower than anticipated, but there are no futility concerns, the DSMB may recommend increasing the total woman-years of follow-up. The DSMB may also recommend other protocol changes.

The DSMB report will consist of open and closed sections. The open section will be made widely available, including to all study investigators, who may choose to share the report with IRBs, donors, and other interested parties. This section will include data in aggregate form (*i.e.* not broken down by study arm), focusing on trial conduct issues such as overall accrual and drop-out rates, eligibility rates and reasons for ineligibility, baseline characteristics and condom use, and compliance with protocol procedures, including diaphragm use. The closed section of the DSMB report will be made available to DSMB members only. This section will include the above data reported by trial arm and data concerning trial endpoints—such as HIV and STI incidence and rates of adverse events—overall and by trial arms and clinical sites.

## 12.7 Final Data Analysis

The final data analysis of the primary and secondary trial objectives will be a joint process between investigators at UCSF, Ibis Reproductive Health, the Medical Research Council, the University of Zimbabwe, and the Perinatal HIV Research Unit. The four co-PIs in consultation with our TAC, will jointly determine the appropriate data analysis and paper writing processes (see Section 15.5). Fine-tuning of the data analysis plan may take place after the trial begins, but it should be complete by the time the interim analysis is conducted. The data analysis plan will be kept in the regulatory binders at the study sites and at UCSF together with the protocol.

Analyses will be performed on two types of analysis sets: the full analysis set (intent-to-treat analysis) and the per-protocol set (per-protocol analysis). The full analysis set will include all eligible, randomized participants. The per-protocol analysis entails further stratification of the full analysis set based on compliance with the study protocol, most notably diaphragm, gel, and condom use. The precise composition of the per-protocol set will be outlined in the data analysis plan. The intent-to-treat analysis provides the most conservative analysis strategy, with randomization fully intact. A per protocol analysis will also be performed to account for reported study product use (adherence), and not just treatment assignment. Per protocol analyses are generally interpreted as less stringent than ITT analyses because other factors may confound the relationship between study product use and HIV/ STI endpoints.

Any differences in results between the two types of analyses will be explicitly discussed and taken into account in the overall interpretation of trial results. Missing values represent another source of potential bias. While every effort will be made to minimize missing data, the data analysis plan will specify how to handle any missing data.

### 12.7.1 Overall Study Performance

We will calculate accrual, retention and eligibility rates, and describe reasons for ineligibility, other non-enrollment, and discontinuation. We will calculate the number of woman-years of follow-up accumulated in each study arm, the total number of skipped and late visits, and the average follow-up period per woman per study arm. We will calculate the total number of coital acts and diaphragm and condom use during coital acts in a variety of ways. If appropriate, we will test for statistical differences in retention and compliance rates between the study arms using generalized estimating equations. We will test for differences in summary variables using standard t-tests for means and Fisher’s exact test for categorical variables.

### Success of Randomization

We will summarize baseline demographic, sexual behavior, and other characteristics assessed at the screening and enrollment visits for each trial arm to assess the success of randomization. Continuous data will be summarized as means and ranges and categorical variables as percentages. If appropriate, we will test differences between the study arms by standard t-tests for means and Fisher’s exact test for categorical variables.

### 12.7.3 Primary Objective and Secondary Objectives 1 & 3: Effectiveness

Cox proportional hazards models, stratified by study site, will be performed to compare the HIV incidence of the diaphragm arm with the incidence in the non-diaphragm control arm. We will consider the diaphragm and gel effective if the HIV infection rate among women randomized to the diaphragm and gel arm is statistically significantly lower than the infection rate among women randomized to the non-diaphragm arm with appropriate adjustment for multiple comparisons and correlated hypothesis tests. Our sample size and power will afford us to have 90% power to detect a relative hazard comparing incidence in women in the diaphragm arm compared to that in the controls of .67 corresponding to an intervention-related reduction of 33% as significantly less than one at the 5% level. Similar analyses will be performed with incident STI infections as secondary endpoints. In the per-protocol analyses, additional co-variates will be included in the hazards models, including frequency of diaphragm, gel, and condom use, and baseline characteristics that were statistically significantly different between the study arms at baseline. We also will conduct analyses, controlling for a number of STIs (*i.e.* TP, HSV-2, and TV), and evaluate these STIs as potential effect modifiers of a diaphragm-HIV relationship. All results will be reported as rate ratios with associated 95% confidence intervals.

### 12.7.4 Secondary Objectives 2 and 4: Acceptability and Feasibility

More details on the analyses will be provided in the separate protocol to be developed. Briefly, we will measure women’s experiences with the diaphragm and gel in structured interviews at follow-up visits and in focus group discussions and/or in-depth interviews using the following groups of variables: 1) general acceptability of the diaphragm and gel (assessments of best and worst features. willingness to recommend to a friend), problems with use and re-use, and factors related to diaphragm hygiene, 2) effects of diaphragm and gel use on sexual activity (*e.g.* changes in sexual pleasure and in coital frequency), ability to hide use from partner when so desired; and 3) general acceptability, and effects of diaphragm and gel on condom use. We will calculate overall rates of key acceptability and feasibility variables, as well as an overall acceptability rating, at the start and end of the trial (visits at month 3 and month 24 or closing visit, if it is before 24 months). We will also calculate factors noted by study staff and ascertained from study and clinic records such as the need for re-fitting and requests for new products. We will also evaluate changes in acceptability over time by comparing key acceptability variables by study arm and by visit, the overall acceptability rating by study arm and by visit, and each woman’s first acceptability report with her last one. We will use generalized estimating equations to perform the first two analyses and McNemar’s Chi-square tests to perform the last. Logistic regression models will be used to evaluate effects of demographic and sexual co-variates on the overall acceptability rating at various time points.

# 13 Human Subjects

## 13.1 IRB Review

The protocol requires that written informed consent must be obtained from all study participants. The protocol has been approved by IRBs of UCSF, Ibis Reproductive Health, MRC, the University of Zimbabwe, and PHRU. The protocol has been shared with the Ministries of Health in South Africa and Zimbabwe and additional local, regional or national ethics approval boards as necessary or required.

## 13.2 Confidentiality

All interviews and clinical exams are conducted in private and all study-related information is stored securely at the study site, in locked file cabinets. All records and specimens are identified by ID numbers only to maintain participant confidentiality. Completed study forms are kept in locked files at the study sites. All records that contain names or other personal identifiers, such as the study register, locator forms, signed informed consent forms, logbooks, cash books and appointment book are kept locked separately from records identified by ID number. All local databases are password-protected. Data that are transmitted electronically do not contain any identifying information. While regulatory agencies, study monitors, and auditors or their designees may have access to study records, the identity of participants remains confidential.

Although study staff make every effort to protect privacy and confidentiality, a participant’s involvement in the study could become known to others and social harms may result if study participation is associated with being at high risk for HIV infection. To minimize such stigma, much emphasis is placed on community outreach to educate the community about HIV/AIDS, diaphragms, and this clinical trial.

## Compensation

Participants receive a monetary reimbursement at each scheduled study visit for transportation to and from the clinic for the clinic-based cohort study, time spent in the study and any other costs which are associated with the volunteer's participation in the study. Participants who return for unscheduled visits or visits that are scheduled for medical follow-up or treatment are reimbursed for transport costs at the discretion of the site coordinators on a case-by-case basis. Site-specific reimbursement amounts are determined by each site and are comparable to amounts given to participants in other studies in the surrounding areas. The determined amount for a site is stated in its Informed Consent Form. All reimbursement amounts are approved by the sites’ local IRBs. Sites may choose to provide reimbursement in the form of food/grocery coupons if they cannot safely keep cash on hand.

## Benefits and Risks

Participation in this trial involves numerous benefits, including diagnosis and treatment of STIs, pregnancy testing, HIV counseling and testing, safer-sex counseling, and free male condoms. Women who test positive for HIV at screening are offered psychosocial support by study staff and psychosocial and medical referral as needed. Women who seroconvert for HIV during the trial receive psychosocial and medical services as needed from study staff or referral to such services in the community as appropriate and feasible at each study site. For example, in Zimbabwe, we have established weekly support groups for HIV-infected women identified in all of our studies. Male partners of participants are eligible for free STIs diagnosis and treatment as well as referral for HIV counseling and testing or provision of free testing at the site. Participants and others may benefit in the future from the trial results. Specifically, the trial results may lead to knowledge about whether the latex diaphragm with Replens® gel can help to protect women from HIV infection.

Study risks are minimal because diaphragms and Replens® gel have been deemed safe by regulatory agencies and have been used widely for decades. However, a small percentage of women may discover that they are allergic to latex and may experience adverse effects before being discontinued from the study. In addition, there may be an increased risk of developing urinary tract infections and Toxic Shock Syndrome with diaphragm use. Furthermore, participants may experience minimal vaginal bleeding and/or discomfort during or after the enrollment pelvic exam. Finally, participants may become embarrassed or anxious when receiving HIV counseling or test results. Being diagnosed with HIV or another STI may cause stress on relationships. Trained counselors are available to help participants with these feelings.

## Trial Closure Considerations

UCSF reserves the right to terminate the trial prematurely at one or more sites if study participant recruitment is too slow, if study participant retention in the study is insufficient, if undue risk related to the study intervention arises, or if there are serious deviations from the study protocol.

# 14 Administrative Procedures

## Investigator Responsibilities

The co-PIs obligate themselves and all study staff to:

- Conduct the study in accordance with the relevant, current protocol, and not make changes without permission of the sponsor (UCSF) except when necessary to protect the safety, rights or welfare of participants.
- Personally conduct or supervise the described investigations.
- Inform participants in the study that use of the study products for the prevention of STIs is under investigation and ensure that requirements relating to obtaining informed consent and IRB review and approval are met.
- Report AEs and SAEs that occur in the course of the investigation to UCSF in accordance with this protocol.
- Understand the designated use of the Ortho All-Flex® diaphragm and Replens® gel, including the potential risks and side effects
- Ensure that all associates, colleagues, and employees assisting in the conduct of the study are informed about their obligations in meeting the above commitments.
- Maintain adequate and accurate records in accordance with this protocol and make those records available for inspection to study monitors and auditors or their designees, and store them at the study site.
- Ensure that an IRB that complies with the requirements of 45 CFR 46 will be responsible for the initial and continuing review and approval of the clinical investigation.
- To promptly report to the IRB all changes in the research activity and all unanticipated problems involving risks to human subjects or others.
- To not make any changes in the research without IRB approval except where necessary to eliminate apparent immediate hazards to human subjects.
- Comply with all other requirements regarding the obligations of clinical investigators and all other pertinent requirements in 45 CFR 46.

As part of the above responsibilities, the study site co-PIs are obliged to maintain a regulatory binder on-site, containing at a minimum the following documents:

- Contractual agreements between UCSF and their site (sub-award documents in the case of the South African sites and Memorandum of Agreement between UCSF and the University of Zimbabwe).
- The original signed study protocol and all amendments, clarifications, addenda, documentation of protocol deviations, *etc*. The co-PIs must sign and date the cover page of this study protocol and all subsequent amendments.
- Ortho All-Flex® manufacturer insert and instructions.
- Replens® manufacturer insert and instructions.
- Locally approved consent forms (English and local language versions), records of IRB approval, records of IRB annual review, and all other IRB correspondence.
- Local approval for importation of study device and accountability records.
- Listing of local laboratories, documentation of their accreditation to perform protocol-specified testing for which they have been contracted, CVs of laboratory staff involved in the testing for this study, and laboratory test normal values.
- Study staff roster, signature log and CVs for all staff members.
- Study monitoring visit log.
- Monitoring and progress reports.

All study sites will weekly progress reports using a format designed jointly by UCSF and Ibis Reproductive Health to the co-PIs, and other key trial management personnel describing the number of participants screened, enrolled and completed, reasons for study discontinuation, numbers of AEs reported, a description of all SAEs, and unanticipated problems.

## 14.2 Study Activation

Following ethical review and approval, study sites submitted all required administrative documentation (see Section 15.1) to UCSF. A comprehensive study training took place when data collection forms were close to final. After the training, final adjustments were made to the SOPs, forms, translations and back-translations. The trial was initiated at each study site independently when the co-PI of that study site and UCSF agreed that all systems were in place.

## Study Monitoring and Independent Audits

UCSF, as the sponsor and overall coordinator of this trial, is responsible for ensuring the proper conduct of the trial. UCSF designates monitoring and quality control staff for the trial. Part of this responsibility is shared with Ibis Reproductive Health staff.

Monitoring is separated into two categories: general monitoring and clinical monitoring. QC procedures for laboratory testing are described in Section 10.3. In addition to the monitoring procedures outlined below, UCSF and Ibis arrange for independent audits of the trial, including laboratory audits. Quintiles Transnational Corporation conducted a complete external audit of each MIRA study site in October-November 2005. Quintiles produced final reports that were addressed by the sites, Ibis and UCSF. Responses to audit findings were submitted to Quintiles.

General monitoring is conducted by our monitors who travel regularly to the three trial sites according to a pre-determined monitoring schedule. The monitoring coordinator trains the monitors and checks their work periodically throughout the trial.

The monitors’ duties are:

- To verify compliance with protection of human subjects and other research regulations and guidelines
- To assess adherence to the study protocol and GCP guidelines
- To confirm the quality and accuracy of information collected at the study site and entered into databases

Study site investigators and staff allow study monitors to inspect study facilities and documentation and to observe the performance of study procedures. As part of the monitoring process, study participants are asked to acknowledge the possibility that a representative of UCSF may request an interview. If an interview is requested, the study participant has the option of accepting or declining the interview. Monitors verify that written informed consent is being obtained from participants, that all information is being kept confidential, that regulatory files are up to date, and that AEs and SAEs are properly documented and reported. They ensure that participants enrolled in the trial are indeed eligible, that follow up is occurring according to protocol, and that reported data are accurate and complete. They inspect storage and inventory of diaphragms and other study supplies to ensure proper accountability. During the monitoring visit, the monitors document all actions they take. After the monitoring visit, they write a monitoring report, which is distributed to the Monitoring Coordinator, Protocol Director, Project Director, Data Manager, and to all co-PIs. The study site will maintain a monitoring log to document all monitoring visits.

Monitoring of clinical procedures is conducted in conjunction with the Medical Advisor on this trial. The Medical Advisor trains or helps develop training curriculum for the study clinicians prior to trial initiation and develops and reviews clinical operating procedures. Periodic monitoring of clinical procedures is conducted throughout the trial, including direct observation of clinical procedures. After monitoring visits, the monitor distributes a report as outlined above.

## Protocol Amendments and Deviations

Changes to the protocol are made only when written protocol amendments signed by the co-PIs have been approved by the relevant IRBs. Protocol amendments may affect consent forms for current and future patients, which have to be modified accordingly and approved by the relevant IRBs.

If a protocol deviation is required to protect the life or physical well being of a participant in an emergency, it may be carried out without prior approval from UCSF or relevant IRBs. The study site co-PI or designee must, however, report the deviation to UCSF co-PI, protocol director and Project Director as soon as possible and no later than five working days after the emergency comes to the attention of the study staff. They also keep a record of all such deviations, which documents the dates of and reasons for each deviation, and report them to the local IRB. The UCSF Project Director notifies the UCSF IRB according to IRB guidelines.

Other deviations from the protocol may not be carried out without prior approval from UCSF and possibly the relevant IRBs when the change involves the rights, safety or welfare of participants. If an inadvertent protocol deviation or incident (unanticipated problem) has occurred, the site investigator or designee reports it to the UCSF Project Director and/or Protocol Director within 5 working days of becoming aware of the event. The UCSF IRB requires that all major violations be reported from all MIRA sites. Major protocol violations are defined as any unapproved changes in the research study design and/or procedures that are within the investigator’s control and not in accordance with the approved protocol that may affect the participant’s rights, safety or well-being, or the completeness, accuracy and reliability of the study data.

## Use of Information, Publishing, and Ancillary Studies

No presentations or publications about any data collected during this trial may be made without the joint approval of all co-PIs. Writing and publication of the main publications, which address the primary and secondary trial objectives outlined in this protocol, will be a joint process between investigators at UCSF, Ibis Reproductive Health, the University of Zimbabwe, the Medical Research Council, and the Perinatal HIV Research Unit. The co-PIs will jointly determine the appropriate manuscript writing, review and publication process for the main publications as well as additional publications that are directly related to the trial.

Ancillary studies are defined as studies that do not address the primary and secondary objectives of the trial, but directly involve trial participants, trial site resources reserved for the trial, and specimens or data collected during the trial as described in this protocol. Examples of ancillary studies include surveys or qualitative research with trial participants, the testing of stored specimens, the addition of specimens or lab tests to the ones described in this protocol, and secondary data analysis of trial data. Proposals for all such studies should be reviewed and approved by the four co-PIs as well as relevant IRBs if applicable before they can be implemented. UCSF reserves the right to overrule decisions made by the co-PIs if deemed necessary to avoid jeopardizing the primary and secondary objectives of the trial.

# 15 References

# 16. Appendices

## Protocol for Bacterial Vaginosis Sub-Study

**An Investigation of the Association Between the Diaphragm used with Replens® and Bacterial Vaginosis**

**Prepared by:**

Craig Cohen, MD, MPH, UCSF

Ariane van der Straten, PhD, UCSF and the MIRA team

Zimbabwe Site:

Tsungai Chipato, MD, Investigator

**A. Introduction**

Bacterial vaginosis, the most common bacterial genital infection in women of reproductive age has been linked to considerable gynecologic and obstetric morbidity. Recent studies demonstrate that BV may be a cofactor in acquisition of human immunodeficiency virus type (HIV) infection. ^1-3^. The highest prevalence of BV has been in sub-Saharan Africa where HIV infection is also common ^2,3^. It has been postulated that BV may increase susceptibility to HIV infection by a variety of mechanisms including possibly altering the host’s defense system through the absence of the normal H_2_O_2_-producing vaginal lactobacillus (which allow to maintain the natural acidic pH of the vagina), with loss of the protective myeloperoxidase halide-hydrogen peroxide system ^4^, and by production of metabolic by-products that may increase HIV replication ^5,6^ or activate target cells for HIV infection ^7^. If any of these hypotheses are correct, reduction of BV prevalence could significantly reduce female HIV acquisition. The high prevalence of BV in sub-Saharan Africa remains unexplained. Though considerable evidence supports sexual transmission of BV, factors like clothing, climate and hygiene may influence vaginal flora. Of the various risk factors associated with BV, the contribution of each to causing BV may vary depending on hygienic, sexual and other practices ^8^. BV treatment with metronidazole was first found to be to be effective in the initial clearing of symptoms and signs of BV ^9^, but has since proven disappointing even in industrialized countries, since recurrence rates reach 40% within 3 to 6 months after treatment with oral or intravaginal medication ^10,11^. In Uganda, administration of metronidazole, azithromycin and ciprofloxacin to large populations of adults of reproductive age every 10-months did not reduce the prevalence of BV below 50% ^12^.

Use of the diaphragm with nonoxynol-9 (N9) based spermicide has been associated with altered vaginal bacterial microflora including decrease rates of lactobacillus colonization ^11^. However, most regard the alteration of vaginal flora and increased risk of urinary tract infection a direct result of the N-9 spermicide and not related to the diaphragm alone ^12^. Importantly, Buffergel® a microbicide with the ability to maintain a low vaginal pH was associated with profound reductions in BV after a short period of use ^13^. Therefore, we propose to study the effect of the diaphragm used with Replens® (a commercially available vaginal lubricant with pH buffering capacity) on the vaginal flora and on the rate of BV in women enrolled in a randomized controlled trial.

**B. The Specific Aims**

**Specific Aim 1:** To compare rates of BV in women in the diaphragm with Replens with those of women in the condom only arm.

- 1. To compare Nugent’s scores in the 2 arms as the full 10 point scoring system during a 12 – 24 month follow-up after randomization, and
  2. To assess the influence of the intervention on vaginal colonization by *Lactobacillus spp*. as note on Gram stain.

**Specific Aim 2:** To determine whether women in the diaphragm used with Replens® arm have an altered rate of recurrence after treatment of BV compared to women in the condom only arm.

**C. Methods**

**C.1 Overview**

At the Zimbabwe site, self-collected vaginal swabs will be collected on the 500 women enrolled in the RCT at their enrollment visit and every three months per study protocol. Data on recent vaginal hygiene and other practices that may affect vaginal microflora will be collected on an additional questionnaire at every follow-up visit. Since the site will enroll over 2,000 women over the course of the randomized trial, enrollment for the BV component will take place at one of the three recruitment centers, and will be closed once the full sample size has been attained.

To study Aim II, 500 women with symptomatic BV will be required. Unfortunately, since we will not be able to perform vaginal Gram stains in real-time, the initial diagnosis of BV will be made using the modified Amsel’s criteria (pH > 4.7, positive whiff test and clue cells on wet mount). Those diagnosed with BV will receive a 5-day course of metronidazole 400 mg three times per day. Vaginal Gram stains will also be collected. Only women with a Nugent score ≥ 7 and with 3 of 3 Amsel’s criteria will be analyzed. Women enrolled in Aim II will have a vaginal Gram stain repeated on a self-collected vaginal swab three months after treatment. The prevalence of BV after three months will serve as the primary endpoint for this aim.

**C.2.1 Analysis of major study endpoints**

**C.2.1.1 Aim 1 major endpoints**

The primary endpoint will be based on Nugent criteria; BV free survival will be defined as Nugent score < 7. Data will be analyzed initially in an intent-to-treat manner comparing the percent of quarterly visits (on average women will undergo 6 follow-up visits) with BV for those assigned to the diaphragm/Replens® arm vs. the condom only arm. A secondary analysis will be performed to compare women compliant with the diaphragm and Replens® to the control arm.

**C.2.1.2 Aim 2 major endpoints**

Similar to Aim 1, the primary endpoint will be based on Nugent criteria; BV free survival will be defined as Nugent score < 7. Data will be analyzed in an intent-to-treat manner comparing survival without BV three-months after treatment for those assigned to the diaphragm/Replens® arm vs. the non-intervention arm.

**C.2.2 Sample size determination, Data Management and statistical analysis**

**C.2.2.1 Aim 1 sample size**

Based on data from other studies conducted on BV in sub-Saharan Africa, we assume that 20% of women will have BV by Gram stain at baseline, and at every visit for those enrolled in the non-intervention condom only arm. We further assume that > 80% of BV will be asymptomatic, and therefore treatment with metronidazole will have a limited effect on the overall prevalence of BV at subsequent visits. The primary outcome will be visit-specific BV prevalence. We assume that outcomes will be compared between arms on an intent to treat basis using generalized estimating equation (GEE) logistic regression model controlling for a within-participant correlation between repeated outcomes of 0.2, and that annual rates of loss to follow-up will be 5%. Based on these assumptions, 250 women per arm should be sufficient to insure 80% power to detect a ± 25% difference in prevalence between the two study arms as significant at the 5% level (based on a two-sided test). This corresponds to a relative risk (odds ratio) of 0.75 (0.70).

**C.2.2.2 Aim 1 major endpoints**

Data will be analyzed in an intent-to-treat manner comparing BV prevalence at 3-monthly quarterly visits (on average women will undergo 6 follow-up visits) between arms using generalized estimating equation (GEE) logistic regression methods.

**C.2.2.3 Aim 2 sample size**

We assume that a minimum 5% of participants will present with symptomatic BV at every visit. Given the projected sample of 2200 women for the Zimbabwe (and a total of nearly 13,000 follow-up visits), this implies that approximately 660 diagnosed cases of symptomatic BV should be observed. The primary outcome for this aim will be recurrence of BV within three months following treatment for symptomatic disease. We assume that recurrence rates will be compared across arms on an intent-to-treat basis using a chi-squared statistic, and that loss-to-follow up rates in a three month visit cycle will not exceed 5%. Given these assumptions, and assuming three-month recurrence rates among women receiving only condoms in the range 0.1 - 0.2, a total sample size of 500 women will provide 80% power to detect a 33% reduction in recurrence associated with treatment at the 5% significance level.

**C.2.2.4 Aim 2 major endpoints**

The primary outcome for Aim 2 will be a binary indicator of BV recurrence observed three-months after treatment. Recurrence proportions will be compared across treatment arms using a chi-squared test.

**D. Literature Cited**

1. Cohen CR, Sinei S, Reilly M, Bukusi E, Eschenbach D, Holmes KK, Ndinya-Achola JO, Bwayo J, Grieco V, Stamm W, Karanja J, Kreiss J. Effect of human immunodeficiency virus type 1 infection upon acute salpingitis: a laparoscopic study. J Infect Dis 1998; 178: 1352-8.
2. Taha TE, Hoover DR, Dallabetta GA, Kumwenda NI, Mtimavalye LA, Yang LP, Liomba GN, Broadhead RL, Chiphangwi JD, Miotti PG. Bacterial vaginosis and disturbances of vaginal flora: association with increased acquisition of HIV. Aids 1998; 12: 1699-706.
3. Martin HL, Richardson BA, Nyange PM, Lavreys L, Hillier SL, Chohan B, Mandaliya K, Ndinya-Achola JO, Bwayo J, Kreiss J. Vaginal lactobacilli, microbial flora, and risk of human immunodeficiency virus type 1 and sexually transmitted disease acquisition. J Infect Dis 1999; 180: 1863-8.
4. Klebanoff SJ, Hillier SL, Eschenbach DA, Waltersdorph AM. Control of the microbial flora of the vagina by H2O2-generating lactobacilli. J Infect Dis 1991; 164: 94-100.
5. Al-Harthi L, Roebuck KA, Olinger GG, Landay A, Sha BE, Hashemi FB, Spear GT. Bacterial vaginosis-associated microflora isolated from the female genital tract activates HIV-1 expression. J Acquir Immune Defic Syndr 1999; 21: 194-202.
6. Sturm-Ramirez K, Gaye-Diallo A, Eisen G, Mboup S, Kanki PJ. High levels of tumor necrosis factor-alpha and interleukin-1beta in bacterial vaginosis may increase susceptibility to human immunodeficiency virus. J Infect Dis 2000; 182: 467-73.
7. Hashemi FB, Ghassemi M, Faro S, Aroutcheva A, Spear GT. Induction of human immunodeficiency virus type 1 expression by anaerobes associated with bacterial vaginosis. J Infect Dis 2000 May; 181: 1574-80.
8. Priestley CJ, Jones BM, Dhar J, Goodwin L. What is normal vaginal flora? Genitourin Med 1997; 73: 23-8.
9. Pheifer TA, Forsyth PS, Durfee MA, Pollock HM, Holmes KK. Nonspecific vaginitis: role of Haemophilus vaginalis and treatment with metronidazole. N Engl J Med 1978; 298: 1429-34.
10. Potter J. Should sexual partners of women with bacterial vaginosis receive treatment? Br J Gen Pract 1999; 49: 913-8.Holmes K, Hillier S. Bacterial Vaginosis. In: KK H, PF S, PA M, SM L, E SW, P P, JN W, eds. Sexually Transmitted Diseases: Third edition Edition. New York: Mc Graw Hill, 1999; pg 563-585.
11. Hooton TM, Roberts PL, Stamm WE. Effects of recent sexual activity and use of a diaphragm on the vaginal microflora. Clin Infect Dis. 1994;19:274-8.
12. Gupta K, Hillier SL, Hooton TM, Roberts PL, Stamm WE. Effects of contraceptive method on the vaginal microbial flora: a prospective evaluation. J Infect Dis. 2000;181:595-601.
13. van De Wijgert J, Fullem A, Kelly C, Mehendale S, Rugpao S, Kumwenda N, Chirenje Z, Joshi S, Taha T, Padian N, Bollinger R, Nelson K. Phase 1 trial of the topical microbicide BufferGel: safety results from four international sites. J Acquir Immune Defic Syndr. 2001;26:21-7.

## Protocol for Human Papillomavirus Sub-Study

**Diaphragms: a novel strategy to prevent cervical cancer and HIV
in low-resource settings?**

**Prepared by:**

George F. Sawaya, MD

Karen Smith-McCune, MD, PhD

Zimbabwe Site:

Zvavahera Michael Chirenje, MD

Tsungai Chipato, MBChB

**Introduction**

The randomized controlled trial of diaphragm use in Zimbabwean women will determine whether provision of diaphragms reduces the rate of male-to-female vaginal HIV transmission. This study is also an unparalleled opportunity to examine the role of the diaphragm in reducing human papillomavirus (HPV) infection. The role of HPV in the development of cervical cancer is well supported by extensive epidemiologic and biological data. As the most prevalent sexually transmitted disease worldwide, genital HPV infection places millions of women at risk for cervical cancer. No randomized controlled trials have been performed to determine the effect of diaphragms on any sexually transmitted infection, including HPV. We propose that diaphragm provision will decrease HPV incidence and persistence, and hence reduce risk of cervical cancer. This straightforward hypothesis can be readily tested with the addition of a limited number of tests to the existing Diaphragm Intervention Study. If substantiated, we may be able to offer women worldwide a simple, affordable, readily available intervention to reduce the risk of cervical cancer.

**Study Hypotheses and Specific Aims**

***1. Diaphragm use will decrease the incidence of HPV.*** We propose to collect samples at each visit for HPV testing and typing by PCR in the randomized cohort of Zimbabwean women. We will determine the effect of diaphragm use on the rates of incident infections.

***2. Diaphragm use will decrease the persistence of HPV.*** We will determine the effect of diaphragm use on persistence of HPV in women found to be positive at enrollment.

***3. Diaphragm use will decrease the incidence of cervical cytologic abnormalities.*** Women will receive cytologic evaluation at enrollment and exit. We will determine the effect of diaphragm use on the rates of cytologic abnormalities.

***4. HPV infection increases the risk of male-to-female vaginal HIV transmission.*** We hypothesize that HPV infection will result in recruitment of a local immune response, which might place women at increased risk for heterosexual HIV acquisition. We will compare the rates of HIV acquisition in HPV-negative *versus* HPV-positive women. In addition, we will examine the effect of HPV viral load on HIV transmission.

**Background**

**Etiology.** The vast majority of squamous-cell cervical cancer is now thought to be the long-delayed consequence of sexually transmitted oncogenic human papillomavirus (HPV) infection ^33^. Women progress from infection, to asymptomatic high-grade preinvasive dysplastic lesions and finally to invasive cancer. Important risk factors for progression to cervical neoplasia include persistence of oncogenic viral types ^34-37^ and high viral loads ^38-40^. Periodic screening offers many opportunities to discover and treat preinvasive lesions before cancer develops ^41^. Widespread screening in the US has been highly successful: a 70% decline in cervical cancer incidence and mortality has been noted in the last 5 decades ^42^.

**Burden of suffering.** An estimated 80% of the 466,000 case of invasive cervical cancer that occur each year are diagnosed among women in developing countries ^43^. From 1988 to 1992, Zimbabwe reported the highest rates (65-70 per 100,000 women) followed by areas of Brazil and Peru ^43^; rates in the United States over that same period ranged from 7 to 12 per 100,000. That cervical cancer is an important cause of morbidity and early mortality among women in the developing world is undisputed. Given that cervical cancer is largely a preventable disease, these statistics are discouraging. In 1995, cervical cancer accounted for 30% of all registered cancers and 80% of all gynecological cancers in Zimbabwe ^44^.

**Obstacles to cervical cancer prevention.** Lack of consumable supplies, qualified staff and access to curative surgical treatments are barriers to establishing effective screening programs in low-resource areas such as Zimbabwe ^44, 45^. Cytology-based programs pose particular quality-control and logistical problems, fostering recent interest in alternative screening programs based on detection of human papillomavirus DNA ^46, 47^ and visual inspection with acetic acid followed by cryotherapy ^48^. All secondary prevention programs, however, rely on providing access to screening modalities and interventions administered by trained personnel and implementation of cervical treatments that can be costly and have inherent risks to women.

**Evidence supporting barrier methods for cervical cancer prevention**. Another approach to cervical cancer control is development and adoption of interventions that decrease exposure to HPV. Despite evidence from observational studies suggesting that barrier contraceptives may provide protection, these methods have received little attention. In 1996, the US Preventive Services Task Force summarized evidence on primary prevention of cervical cancer ^49^. The Task Force found that use of barrier contraception (diaphragm and condoms) and spermicides (foam or contraceptive jelly) was associated with lower risk of invasive cervical cancer. Of 11 case-control studies and one cohort study examined, 10 reported that at least one of these methods of contraception was associated with a significantly lower risk. The protective effect persisted after controlling for the potential influence of age at first intercourse, cytology screening history and smoking. Substantial reductions in risk of invasive cervical cancer were observed among both condom users (odds ratios = 0.4-0.8) ^50^ and diaphragm users (OR = 0.2-0.7) ^51, 52^. A longer duration of use was associated with greater protection ^50^. A recent study indicated that longer duration of barrier method use decreased risk of cervical preinvasive lesions specifically among women positive for high-risk HPV types ^53^.

**Potential associations between HPV infection and HIV acquisition**. The immune system plays an important role in the natural history of HPV infection. Over 70 types of HPV have been identified, of which over 30 can infect genital epithelium. HPV infection occurs after genital contact and can result in a spectrum of disease encompassing genital warts, precancerous changes known as dysplasia, and invasive cancer. Typically, however, HPV infection is transient, clinically unapparent, resolves spontaneously, and results in subsequent protection against re-infection with that particular HPV type ^37^. These observations explain the excitement regarding potential HPV vaccines for preventing cervical cancer ^54^. Consistent with a pivotal role for the immune system in combating HPV infection are clinical observations that immunocompromised individuals are more likely to be infected with HPV, to harbor multiple HPV types, to develop precancer as a result of HPV infection, and to recur after therapy ^55-58^. The finding of dense infiltrates of lymphocytes in regressing genital warts also argues that the local immune response plays an important role in the natural history of genital warts ^59^. The immunological activity resulting from HPV infection may increase the risk of vaginal acquisition of HIV by increasing the local presence of immune cells susceptible to HIV infection.

The cervical transformation zone consists of the junction of two distinct epithelial surfaces, the columnar epithelium of the endocervix and the squamous epithelium of the ectocervix. The interface of 2 epithelial surfaces is a rare anatomic occurrence in the human body, and results in a biological phenomenon known as metaplasia, involving ongoing cellular transformation from columnar to squamous histology. Hence the transformation zone is a site of unique biological activity. Of particular relevance for this proposal is the finding that the majority of dysplasia caused by HPV occurs on the cervix, within 1 cm of the transformation zone. The transformation zone is also an active immunologic site capable of eliciting a robust secondary immune response. Germinal centers, which are sites of antigen presentation, B cell clonal proliferation, and T cell activation, are rare in normal cervical transformation zone but are abundant in association with high-grade dysplasia ^60^. A subset of germinal centers associated with dysplasia contains HPV antigens, indicating that the local immune response is directed in part against HPV. The Smith-McCune lab has also quantified local immune cell infiltrates in normal cervical transformation zone compared to areas of high-grade dysplasia and found high grade dysplasia contains significantly elevated numbers of CD4+ and macrophages than normal cervical epithelium. These results indicate that target cells of HIV infection are increased as a result of HPV-induced disease and suggest that HPV, like other STIs, might enhance the local susceptibility to HIV infection by recruiting susceptible immune cells to the site of infection.

**Unparalleled opportunity.** Given the absence of randomized, controlled trial data and the potential for confounding, the US Preventive Services Task Force concluded that evidence was insufficient to recommend for or against routine counseling of women about use of barrier methods for cancer prevention, although they recommended that women be informed about this potential benefit as part of contraceptive counseling. The straight-forward hypothesis that diaphragms will reduce HPV incidence and persistence can be readily tested and if substantiated, will provide a simple, affordable, readily available intervention to reduce the risk of cervical cancer for women worldwide.

**Methods**

This study will be offered to women enrolled in the Zimbabwe cohort of the diaphragm effectiveness trial (MIRA). Separate consent will be required to participate in this sub-study. Enrollees will be informed of the following additional testing to be performed (See Chart A: HPV study flow sheet). Neither study participants nor providers will be informed of HPV results nor will the results be used to direct clinical management.

• Enrollment visit: cytology and cervical swab for HPV testing

• 3 month visit: self-swab for HPV testing

• 6 month visit: self-swab for HPV testing

• 9 month visit: self-swab for HPV testing

• 12 month visit: self-swab and cervical swab for HPV testing

• 15 month visit: self-swab for HPV testing

• 18 month visit: self-swab for HPV testing

• 21 month visit: self-swab for HPV testing

• 24 month visit or last visit: cytology and cervical swab for HPV testing

**Cytologic sample collection and analysis:** At the time of the enrollment visit pelvic examination, a sample for cytology will be obtained from the endo- and ecto-cervix with a spatula and an endocervical brush. The specimen will be smeared onto a glass slide and then fixed immediately using hairspray (or another fixative). Cytology will be stained and interpreted in Zimbabwe where reliable cytology services have been available for the past 10 years (personal communication, M. Chirenje). Cytopathologists will be blinded to the arm of the study to which participants have been randomized.

**HPV sample collection:** At the enrollment visit, after the cytology sample is taken, the cervix and endocervix will be swabbed under direct visualization with a Dacron swab and stored in Sample Transport Medium (Digene Diagnostics, Silver Spring, MD) at 4^0^C.. At follow-up visits, participants will be instructed in the technique of self-sampling and these samples will be stored in specimen transport medium as stated above. At the 12 month follow-up visit, a clinician-collected swab will be collected in addition to the self-collected swab. At study exit, a final clinician-collected swab will be obtained under direct visualization at the time of pelvic examination. Study exit will vary from 12 to 24 months depending on time of enrollment. Samples will be shipped to UCSF with cold packs for analysis in the laboratory of Dr. Joel Palefsky by PCR using MY09/11 primers (see below). In addition, quantification of HPV levels will be performed by real-time PCR in HPV-positive samples from all women who sero-convert to HIV and a matched sample of controls who do not sero-convert. These will be reported as viral loads. Currently Dr. Palefsky’s laboratory has PCR primers and probes for quantitative PCR in HPV 16, 18 and 33-positive samples, and is developing assays for the remaining high risk HPV types.

**Secondary study outcome: Cervical dysplasia, biopsy-confirmed.** Women with abnormal Pap tests interpreted as AGUS, HSIL, adenocarcinoma in situ and cancer will be referred for immediate colposcopy. Women with Pap results of ASCUS or LSIL will undergo a repeat Pap smear after 6 months. If the results indicate ASCUS or worse, she will be referred for colposcopy. At the time of the exit Pap test, the threshold for referral will remain the same as at previous visits (cytologic abnormalities of AGUS, HSIL or worse). Women with exit Pap results of ASCUS or LSIL will be counseled and referred to local clinics for repeat Pap smears 6 months later. Colposcopists will be blinded to the arm of the study to which participants have been randomized. Women with colposcopic abnormalities will undergo diagnostic biopsies, and those with cervical intraepithelial neoplasia (CIN) 2 or 3 will undergo loop electrosurgical excision procedure. Women found to have cancer will be referred for cancer treatment. Histological analyses of biopsy and loop specimens will be performed in Zimbabwe. All colposcopic and histological data will be entered on a case report form and transferred to UCSF.

**Choice of tests: Cytology:** Two major methods by which cervical cytology is collected and prepared are currently used in research protocols: conventional cytology and liquid-based cytology. We will perform conventional cytology since the laboratory in Zimbabwe does not process liquid-based tests and is unlikely to do so in the near future. Also, liquid-based preparations have been shown in a randomized, controlled trial to increase the likelihood that a test will be wholly unsatisfactory and require a return visit and repeat pelvic examination ^61^. We would like to minimize return visits for this large number of participants.

**Choice of tests: HPV determination.** We will perform PCR for HPV detection because it is an extremely sensitive test that provides information on the presence of 29 individual HPV types and one group of 10 less common HPV types. In addition, PCR detects both high- and low-level infection. We will do type-specific classification to determine the various prevalences of HPV types. We will then be able to analyze our results for individual HPV types and will group them according to their association with cancer, as measured in a large international study of cervical cancer ^62^.

**HPV testing protocols.** HPV testing will be performed in the laboratory of co-investigator Dr. Joel Palefsky. After collection, the brush for HPV testing will be placed in 1 ml of Sample Transport Medium (Digene Diagnostics, Silver Spring, MD). This quantity is more than sufficient to perform all HPV testing. The tubes will be transported to Dr. Palefsky’s lab. Two hundred µL of specimen are used for PCR. PCR is performed using MY09/MY11 consensus HPV L1 primers as well as primers for amplification of the human beta-globin gene. After 30 amplification cycles, 6 µL of amplification mixture are applied to a nylon membrane and probed with a biotin-labeled HPV L1 consensus probe mixture. A separate membrane is probed with a biotin-labeled probe to the human beta-globin gene. Specimens positive with the HPV consensus probe mixture are then studied to determine the specific HPV type by preparing membranes as described above with 6 µL of specimen. The membranes are studied with probes to 29 different HPV types (6, 11, 16, 18, 26, 31, 32, 33, 35, 39, 40, 45, 51, 52, 53, 54, 55, 56, 58, 59, 61, 66, 68, 69, 70, 73, AE2, Pap 155, Pap 291, as well as the following 10 HPV types together in a probe mixture: HPV 2, 13, 34, 42, 57, 62, 64, 67, 72, and W13B). Specimens that are positive with the generic probe, but negative for the 39 specific types are considered to have an "unknown" HPV type.

**Testing quality control.** Dr. Palefsky’s laboratory performs extensive quality assurance and quality control measures with each HPV assay. Because of its high sensitivity, PCR by definition must be performed with great care to avoid the possibility of contamination. The laboratory takes all possible precautions in handling samples, including separation of pre- and post-amplification products in different parts of the research building. Each run contains several random negative control specimens and positive controls for each HPV type being sought. His laboratory will also use HPV-positive tissue culture cells containing known numbers of HPV 16 copies as a sensitivity control. If any of the above negative and positive controls do not work, the reaction is repeated. The lab performs a large volume of HPV testing for several studies and to date has not had problems with contamination. Each specimen is also studied for the presence of beta-globin as a positive control for human DNA and for the overall amplification procedure. If a specimen is negative for beta-globin it is presumed that an inadequate specimen was obtained, that the reaction failed or that the specimen contained an inhibitor of the PCR reaction. In their experience this occurs in less than 5% of the specimens, and the HPV results from the specimen are excluded from analysis. As a final quality control measure, Dr. Palefsky’s laboratory participates in routine exchange of cervical specimens with the other laboratory performing PCR in the WIHS, the laboratory of Dr. Robert Burk. The results of PCR detection are compared between the two labs, including the detection of specific types. In the past they have had excellent agreement and continue to do so.

For cytology quality control, a random sampling of 10% of normal and 100% of abnormal cytologic samples will be shipped to UCSF and reviewed by co-investigator Dr. T. Darragh of the Department of Pathology at UCSF. Due to regulatory constraints in Zimbabwe, histologic specimens cannot be released for outside review.

**Definitions of abnormal tests.**  All cytologic specimens will be interpreted using the 1991 Bethesda System ^63^. As per standard practice, we will consider a Pap smear “abnormal” if interpreted as atypical squamous cells (ASCUS), low-grade squamous intraepithelial lesion (LSIL), atypical glandular cells (AGUS), high-grade squamous intraepithelial lesion (HSIL) or squamous cell carcinoma.

All HPV tests will be reported as positive for high-risk types, positive for low-risk types, positive for both, or negative for both. High-risk types will be defined as the presence of types 16, 18, 26, 31, 33, 35, 39, 45, 51, 52, 53, 56, 58, 59, 66, 68 and 73 ^33^. Low-risk types will be defined as 6, 11, 40, 42, 54, 61, 70 and 72 ^33^. If the beta-globin control is positive but no HPV is detected, the specimen will be considered “negative.”

**Statistical methods and analyses.** HPV prevalence will be estimated as the observed proportion positive, and associated precision summarized using a 95% exact binomial confidence interval. Incidence of HPV during follow-up will be investigated using survival analysis techniques appropriate for grouped or discrete time outcomes to account for intervals between visits. Initial analyses will include incidence over follow-up of particular types or groups of types among women confirmed to be negative at the baseline visit and will be summarized using life table estimates and 95% confidence intervals. We will use a similar approach in investigating persistence by examining differences in clearance among prevalent positives in both randomized groups. To accommodate repeated episodes of infection and clearance for high-risk types, we will also investigate the use of Markov models for panel data, taking negative and positive HPV tests at study visits as the observed states. These models will allow all available follow-up data to be used, provide direct estimates of the transition probabilities between positive and negative states and allow time-varying risk factors to be investigated using a proportional hazards model similar to those described above. Outcomes of viral load and cytologic and histologic abnormalities will be analyzed similarly.

For study aim #4, we will compare the prevalence rates of HPV (e.g., all HPV types, low-risk, types, high-risk types) between sero-converters and non-sero-converters using matched analysis controlled for confounders. We will separately evaluate the potential association between sero-conversion and HPV viral load using a 3-to-1 control-to-case ratio rather than the entire cohort of non-sero-converters since this assay is relatively expensive to perform.

**Statistical power.** For study aims #1-3, we calculated minimal detectable relative hazards for the main study outcomes based on the sample size of 2,200 enrollees with a 10% loss-to-follow-up rate, a power of 80% and a significance level of 5% (one-tailed alpha=0.05) using the log-rank test. We chose one-sided tests for this analysis since we do not believe that diaphragms increase risk of HPV outcomes. We performed analyses over a range of plausible prevalence rates of HPV (up to 40% for all types and as low as 20% for high-risk types) and evaluation periods (12 to 24 months). Prior studies of Zimbabwean sero-negative women have indicated a prevalence of HR HPV types of 28% ^64^. We assumed an annual HR HPV incidence rate of 8% and an annual clearance of 65% in the non-diaphragm arm based on studies from a high-risk area of Brazil ^65^. The prevalence of cytologic abnormalities was estimated at 15% and incidence at 10% per year in this high-risk population in the non-diaphragm arm. High-grade cervical intraepithelial neoplasia (CIN 2 or worse) prevalence was estimated at 5.9% based on a prior cross-sectional study in Zimbabwe ^64^. Assuming a cytology sensitivity of 62% ^64^ and an annual incidence rate of 0.5%, we estimated a prevalence of CIN 2 or worse of 3% by the end of the study in the non-diaphragm arm. For study aim #4, we assumed that 88 women would sero-convert to HIV-positive over the course of the study. We determined the minimal detectable odds ratios for a range of prevalence rates of predictors (e.g., HPV) at 80% power and one-tailed alpha of 0.05.

Table 1 details all power analyses for study aims #1-3. Under a variety of assumptions, we will have power to detect 16-37% reductions in incidence and persistence of HPV between the diaphragm group and the non-diaphragm group. We will also have power to detect at least a 26% reduction in incidence of cytological abnormalities. We will have less power to be able to detect differences in biopsy-proven CIN 2 or worse (a secondary study aim): a minimum 63% reduction with diaphragm use must be demonstrable for us to be able to detect a statistically significant effect. For study aim #4, we will have adequate power to detect differences in HPV prevalence between sero-converters (cases) and non-sero-converters (controls) if control prevalence rates are 20-40% and cases have at least 2.2 times the prevalence of HPV (Table 2). We believe that we will have adequate power for this analysis; a prior study, for example, has shown that in Zimbabwean women, the prevalence of HR HPV types is over two-fold greater in HIV-positive women (64%) compared to HIV-negative women (28%) ^64^. Our main hypothesis for study aim #4 involves evaluating a variety of predictors with various prevalence rates (e.g., all HPV types, low-risk, types, high-risk types), and we will have similar power to detect differences for other predictors in this range.

**Table 1:** Least detectable hazard ratios for various HPV-related outcomes over time assuming a sample size of 2,200 enrollees with a 10% loss-to-follow-up rate, a power of 80% and a one-tailed alpha=0.05)

| **Study Aim** | **Outcome** | **Baseline prevalence** | **Observation period (months)** | **Least detectable Relative Hazard** |
| --- | --- | --- | --- | --- |
| **#1** | **HPV incidence**  All types  High-risk types |  | | |
|  |  | 40% | 12 | 0.63 |
|  |  | 20% | 24 | 0.74 |
| **#2** | **HPV persistence**  All types  High-risk types |  | | |
|  |  | 40% | 12 | 0.77 |
|  |  | 20% | 24 | 0.84 |
| **#3** | **Cytologic abnormality incidence** | 15% | 24 | 0.74 |
| **Secondary** | **Cervical intraepithelial neoplasia incidence** | 4% | 24 | 0.37 |

**Table 2:** Study aim #4 power: least detectable odds ratios for risk factor prevalences of 10-40% assuming 88 sero-converters and 2,112 non-sero-converters (power=80%, one-tailed alpha=0.05)

| **Risk factor prevalence (e.g., HPV)** | **Least Detectable Odds Ratio** |
| --- | --- |
| 10% | 2.8 |
|  |  |
| 20% | 2.3 |
|  |  |
| 30% | 2.2 |
| 40% | 2.2 |

**Limitations**

1) Generalizability. As with all population-based studies, our results may not be generalizable to other groups of women. We anticipate that our findings will be generalizable to other groups of similarly-aged women in settings with similar prevalence rates of HPV and adherence to diaphragm use.

2) Co-interventions. Although randomization will allow for equal distribution of both known and unknown confounders, the nature of the trial and intervention may allow for co-interventions that account for differences that may be observed between the two groups. For example, women who use the diaphragm may be more apt to douche which may in turn affect prevalence rates of HPV. We will collect information on plausible interventions and control for these in the analyses. In addition, the lubricant gel may interfere with the PCR-based test for HPV. We are performing a pilot study to determine any interference. If interference is demonstrable, we will stratify analyses by time from last diaphragm use. We do not believe that the gel will affect cytologic or histologic outcomes nor will it affect enrollment HPV results obtained before the subject uses the diaphragm.

3) Power. Although we believe that we will have adequate power to test our main study hypotheses, we may have limited power to perform subgroup analyses that may be important.

4) Subject adherence to follow-up and protocol. We have devised plans to assure subject retention. We will stress the importance of adherence to study protocol and will assure that women have all the information to make informed choices about participation in all aspects of the study,

**Protection of Human Subjects**

**Risks.** Participation in research may result in a loss of privacy, although safeguards to be described below will make this risk minimal. In general, the risks of this study are small. The major downside of this study is the time requirements imposed for completing all of the study visits. Colposcopy is a safe procedure, however, as with all procedures, there are risks. The risks include discomfort, pain, bleeding and infection. The procedure is well-tolerated by most women with pain associated with biopsy often described as mild to moderate and of short duration. Bleeding from biopsy sites is easily controlled by safe chemical cautery agents. Infection from this procedure is very rare.

**Disclosure of HPV results.** Results of HPV testing will not be disclosed to participants since the tests that will be used are not approved for clinical use by the Clinical Laboratory Improvement Act regulations. HPV infections are very common in this and other populations. There are no known treatments for HPV infection.

**Minimizing risks.** All materials with patient identifiers will be stored in a secure area. Study samples will be labeled only with a study code. Linkage between study codes and patient identifiers will be kept confidential. Therefore no patient identifiers will be attached to samples provided to this project. All colposcopies will be performed by trained professionals.

**Benefits.** Subjects returning to the clinic specifically for procedures related to this sub-study will receive a monetary reimbursement from participation. Otherwise, they will receive the standard reimbursement for their participation in the main study for each scheduled visit. Some women with cervical dysplasia and treatment, however, will benefit by having cancer averted. The improved understanding of the effect of diaphragm provision on HPV and cervical neoplasia outcomes may potentially be of benefit to large populations of similar women and may improve patient management.

**Consent Process and Documentation.** Participants will be required to give additional informed consent for procedures related to the sub-study. The option to participate is explained during the enrollment main study informed consent process. The decision to participate in the sub-study will in no way affect their ability to participate in the main study. All information in the informed consent forms will be translated into Shona and back-translated into English to ensure accuracy and clarity of the information being presented to eligible participants.

**Treatment and Compensation for Injury.** We are aware of the University of California policy on subjects’ treatment and compensation for injury as a result of participation in the proposed study. We have incorporated the UCSF required wording into the consent document. We anticipate no injuries will occur by subject participation.

**Alternatives.** Available treatment options will be unaffected by the subjects’ decision concerning participation in the proposed study.

**Confidentiality of Records.** We will take extensive precautions to maintain patient confidentiality throughout the study. No names or other identifying information will appear on any documents. While the study is on-going, we will keep patients’ names confidential through the use of patient ID numbers. We need to keep these identifiers to link measures at enrollment to measures at the study conclusion. We will maintain this information in locked files; after the study is complete we will destroy records linking patients’ names to study ID numbers. Patient identities will not be revealed in any publication that may result from the proposed study.

**Study Investigators**

**George F. Sawaya, MD:** With dual clinical training in obstetrics/gynecology and fellowship training in epidemiology/biostatistics, Dr. Sawaya brings a strong perspective for evidence-based effectiveness of clinical interventions used for cervical cancer screening. He is a board-certified obstetrician-gynecologist and provides care primarily at San Francisco General Hospital, where he directs the Colposcopy and Cervical Dysplasia Clinic. As Principal Investigator for a current contract with the Centers for Disease Control and Prevention, he is evaluating the National Breast and Cervical Cancer Early Detection Program screening guidelines in terms of the cost-effectiveness and cost-utility of various screening strategies. He is a co-investigator at the Medical Effectiveness Research Center for Diverse Populations at UCSF, a center dedicated to the addressing health disparities in minority populations. He has recently served as the Research Coordinator of the UCSF-Stanford Evidence-based Practice Center, a center focused on conducting high-quality systematic literature reviews (meta-analyses) and technology assessments. He has designed and currently teaches the curriculum in evidence-based medicine and diagnostic testing that is required for all medical students at UCSF.

**Karen Smith-McCune, MD, PhD:** With dual training in obstetrics/gynecology and molecular biology, Dr. Smith-McCune brings a strong basic science perspective and an interest in understanding the possible role of HPV as a cofactor in HIV transmission. Her basic science interests are directed to the basic pathophysiology of cervical dysplasia, specifically to understanding the role of angiogenesis in the persistence and progression of dysplasia. Her current research focus is development of a molecular diagnostic for cervical cancer screening that can be used at the point of care or by the patient herself as a means to increase access to screening in under-served populations. Her research efforts are funded by the NIH and the American Cancer Society. As Director of the UCSF Dysplasia Clinic, she has direct experience with the clinical, personal and societal impact of the HPV epidemic. This perspective motivates her desire to discover and implement simple, affordable and effective interventions to reduce the impact of HPV infection on women’s lives.

**UCSF Co-Investigators**

**Teresa Darragh, MD, Co-Investigator,** is an Associate Clinical Professor of Pathology and Obstetrics & Gynecology at UCSF. She is a recognized national expert on the pathology and colposcopy of HPV-related lesions of the anogenital canal. Over the last 12 years, she has collaborated on numerous studies, both at UCSF and inter-institutionally, providing her expertise in the interpretation of cytology and biopsy specimens from the anogenital canal. In this study, she will be responsible for the interpretation of the cytology and histology specimens.

**Anna-Barbara Moscicki**, **MD, Co-Investigator,** is a Professor of Pediatrics at UCSF. Dr. Moscicki is principal investigator of a large 13-year epidemiological study of human papillomavirus and has extensive experience in HPV testing and molecular epidemiology studies. Dr. Moscicki is involved in a NIH cooperative study of HIV and AIDS in adolescents and is the Chair of the Basic Science Group as well as representing one of the repository sites. She was involved in detailed protocol development of this project that involves 16 different sites in 13 U.S. cities. Dr. Moscicki’s background in epidemiology, virology and immunology will make her an invaluable senior member of the study team.

**Joel Palefsky, MD, Co-Investigator,** is a Professor of Medicine and Laboratory Medicine at UCSF. He is a recognized national expert on HPV. Dr. Palefsky and his group have over 10 years’ experience testing samples from the anus and cervix for HPV, and his work has established the standard of care for anal cancer screening and treatment. His laboratory is one of two nationwide responsible for testing samples in the Women’s Interagency HIV Study. Between his own ongoing studies and those of the WIHS, his laboratory performs PCR on over 3,000 samples per year. He will be responsible for reporting laboratory results to the group and will lend his expertise in the analysis and interpretation of HPV type-specific clinical outcomes and HPV viral loads.

**University of Zimbabwe/UCSF**

**Tsungai Chipato, MBChB, Co-Investigator,** is a Senior Lecturer for the Department of Obstetrics and Gynaecology at the University of Zimbabwe (UZ). He currently serves as the primary investigator of the UZ-UCSF HIV Prevention Trials Unit and oversees multiple studies, including those that fall under the UZ-UCSF Collaborative Research Program to which the MIRA study belongs. For this study, Dr. Chipato will oversee the colposcopy and related follow-up procedures with Dr. Chirenje

**Zvavahera Michael Chirenje, MD, Co-Investigator,** is an Associate Professor/Consultant Gynaecologist for the Department of Obstetrics and Gynaecology at the University of Zimbabwe. His current research includes a diaphragm safety trial, cervical cancer screening and treatment, vaginal microbicides for HIV/STI prevention, immunological studies of HIV-1, and risk of HIV acquisition with hormonal contraception. For this study, Dr. Chirenje will oversee the colposcopy and related follow-up procedures with Dr. Chipato.

**References**

1. Munoz N, Bosch FX, de Sanjose S, Herrero R, Castellsague X, Shah KV, Snijders PJ, Meijer CJ. Epidemiologic classification of human papillomavirus types associated with cervical cancer. N Engl J Med 2003;348(6):518-27.

2. Kjaer SK, van den Brule AJ, Paull G, Svare EI, Sherman ME, Thomsen BL, Suntum M, Bock JE, Poll PA, Meijer CJ. Type specific persistence of high risk human papillomavirus (HPV) as indicator of high grade cervical squamous intraepithelial lesions in young women: population based prospective follow up study. Bmj 2002;325(7364):572.

3. Ho GY, Burk RD, Klein S, Kadish AS, Chang CJ, Palan P, Basu J, Tachezy R, Lewis R, Romney S. Persistent genital human papillomavirus infection as a risk factor for persistent cervical dysplasia. J Natl Cancer Inst 1995;87(18):1365-71.

4. Wallin KL, Wiklund F, Angstrom T, Bergman F, Stendahl U, Wadell G, Hallmans G, Dillner J. Type-specific persistence of human papillomavirus DNA before the development of invasive cervical cancer. N Engl J Med 1999;341(22):1633-8.

5. Wright TC, Jr., Schiffman M. Adding a test for human papillomavirus DNA to cervical-cancer screening. N Engl J Med 2003;348(6):489-90.

6. Sun CA, Lai HC, Chang CC, Neih S, Yu CP, Chu TY. The significance of human papillomavirus viral load in prediction of histologic severity and size of squamous intraepithelial lesions of uterine cervix. Gynecol Oncol 2001;83(1):95-9.

7. Sun CA, Liu JF, Wu DM, Nieh S, Yu CP, Chu TY. Viral load of high-risk human papillomavirus in cervical squamous intraepithelial lesions. Int J Gynaecol Obstet 2002;76(1):41-7.

8. Schlecht NF, Trevisan A, Duarte-Franco E, Rohan TE, Ferenczy A, Villa LL, Franco EL. Viral load as a predictor of the risk of cervical intraepithelial neoplasia. Int J Cancer 2003;103(4):519-24.

9. Sawaya GF, Brown AD, Washington AE, Garber AM. Clinical practice. Current approaches to cervical-cancer screening. N Engl J Med 2001;344(21):1603-7.

10. Ries LAG, Kosary CL, Hankey BF, Miller BA, Clegg L, Edwards BK. SEER Cancer Statistics Review, 1973-1994, National Cancer Institute. NIH Pub. No. 97-2789, Bethesda, MD, 1997.

11. Cancer incidence in five continents. Volume VII. IARC Sci Publ 1997(143):i-xxxiv, 1-1240.

12. Chirenje ZM, Rusakaniko S, Kirumbi L, Ngwalle EW, Makuta-Tlebere P, Kaggwa S, Mpanju-Shumbusho W, Makoae L. Situation analysis for cervical cancer diagnosis and treatment in east, central and southern African countries. Bull World Health Organ 2001;79(2):127-32.

13. Chirenje ZM, Rusakaniko S, Chipato T, Mpanju-Shumbusho W, Ngwalle E, Kirumbi LW, Makuta-Tlebere P, Kaggwa S. Situation analysis for cervical cancer diagnosis and treatment in Zimbabwe. Cent Afr J Med 1998;44(12):307-10.

14. Wright TC, Jr., Denny L, Kuhn L, Pollack A, Lorincz A. HPV DNA testing of self-collected vaginal samples compared with cytologic screening to detect cervical cancer. Jama 2000;283(1):81-6.

15. Belinson J, Qiao YL, Pretorius R, Zhang WH, Elson P, Li L, Pan QJ, Fischer C, Lorincz A, Zahniser D. Shanxi Province Cervical Cancer Screening Study: a cross-sectional comparative trial of multiple techniques to detect cervical neoplasia. Gynecol Oncol 2001;83(2):439-44.

16. Chirenje ZM, Chipato T, Kasule J, Rusakaniko S. Visual inspection of the cervix as a primary means of cervical cancer screening: results of a pilot study. Cent Afr J Med 1999;45(2):30-3.

17. U.S. Preventive Services Task Force. Guide to clinical preventive services: an assessment of the effectiveness of 169 interventions. Report of the U.S. Preventive Services Task Force. Baltimore, Md.: Williams & Wilkins; 1996.

18. Peters RK, Thomas D, Hagan DG, Mack TM, Henderson BE. Risk factors for invasive cervical cancer among Latinas and non-Latinas in Los Angeles County. J Natl Cancer Inst 1986;77(5):1063-77.

19. Wright NH, Vessey MP, Kenward B, McPherson K, Doll R. Neoplasia and dysplasia of the cervix uteri and contraception: a possible protective effect of the diaphragm. Br J Cancer 1978;38(2):273-9.

20. Parazzini F, Negri E, La Vecchia C, Fedele L. Barrier methods of contraception and the risk of cervical neoplasia. Contraception 1989;40(5):519-30.

21. Coker AL, Sanders LC, Bond SM, Gerasimova T, Pirisi L. Hormonal and barrier methods of contraception, oncogenic human papillomaviruses, and cervical squamous intraepithelial lesion development. J Womens Health Gend Based Med 2001;10(5):441-9.

22. Koutsky LA, Ault KA, Wheeler CM, Brown DR, Barr E, Alvarez FB, Chiacchierini LM, Jansen KU. A controlled trial of a human papillomavirus type 16 vaccine. N Engl J Med 2002;347(21):1645-51.

23. Maiman M. Management of cervical neoplasia in human immunodeficiency virus-infected women. J Natl Cancer Inst Monogr 1998;23:43-9.

24. Penn I. Cancers of the anogenital region in renal transplant recipients. Analysis of 65 cases. Cancer 1986;58(3):611-6.

25. Vernon SD, Holmes KK, Reeves WC. Human papillomavirus infection and associated disease in persons infected with human immunodeficiency virus. Clin Infect Dis 1995;21 Suppl 1:S121-4.

26. Fowler MG, Melnick SL, Mathieson BJ. Women and HIV. Epidemiology and global overview. Obstet Gynecol Clin North Am 1997;24(4):705-29.

27. Stanley MA. Immunobiology of papillomavirus infections. J Reprod Immunol 2001;52(1-2):45-59.

28. Kobayashi A, Darragh T, Herndier B, Anastos K, Minkoff H, Cohen M, Young M, Levine A, Ahdieh L, Weinberg V, Hyun W, Greenblatt R, Smith-McCune. K. Lymphoid Follicles are Generated in High Grade Cervical Dysplasia and have Differing Characteristics Depending on HIV Status. Am J Pathol. 2002;160(1541-64).

29. Obwegeser JH, Brack S. Does liquid-based technology really improve detection of cervical neoplasia? A prospective, randomized trial comparing the ThinPrep Pap Test with the conventional Pap Test, including follow-up of HSIL cases. Acta Cytol 2001;45(5):709-14.

30. Bosch FX, Manos MM, Munoz N, Sherman M, Jansen AM, Peto J, Schiffman MH, Moreno V, Kurman R, Shah KV. Prevalence of human papillomavirus in cervical cancer: a worldwide perspective. International biological study on cervical cancer (IBSCC) Study Group. J Natl Cancer Inst 1995;87(11):796-802.

31. Solomon D, Davey D, Kurman R, Moriarty A, O'Connor D, Prey M, Raab S, Sherman M, Wilbur D, Wright T, Jr., Young N. The 2001 Bethesda System: terminology for reporting results of cervical cytology. Jama 2002;287(16):2114-9.

32. Womack SD, Chirenje ZM, Gaffikin L, Blumenthal PD, McGrath JA, Chipato T, Ngwalle S, Munjoma M, Shah KV. HPV-based cervical cancer screening in a population at high risk for HIV infection. Int J Cancer 2000;85(2):206-10.

33. Franco EL, Villa LL, Sobrinho JP, Prado JM, Rousseau MC, Desy M, Rohan TE. Epidemiology of acquisition and clearance of cervical human papillomavirus infection in women from a high-risk area for cervical cancer. J Infect Dis 1999;180(5):1415-23.

**Chart A:** HPV Study Flow Sheet

| **Test** | **Visit** | | | | | | | | |
| --- | --- | --- | --- | --- | --- | --- | --- | --- | --- |
|  | **1*** | **2** | **3** | **4** | **5** | **6** | **7** | **8** | **9†** |
| Cytology | X |  |  |  |  |  |  |  | X |
| HPV, direct test by clinician | X |  |  |  |  | X |  |  | X |
| HPV, self-collected test |  | X | X | X | X | X | X | X |  |

*enrollment

†exit

NOTE: Exit will vary between visit #5 and #9 depending on time of enrollment.

Abnormal cytology will be triaged according to the algorithm in Chart B.

**Chart B:** Initial triage algorithm for Diaphragm/HPV study: first 12 months

This protocol is similar to that in place at the clinical site in Harare, Zimbabwe as per communication with M. Chirenje and T. Chipato

| **Cytologic abnormality** | **Initial Follow-up Strategy** |
| --- | --- |
| **Atypical Squamous Cells of Undetermined Significance (ASCUS)** | Repeat cytology at 6 months*  a) If normal, repeat cytology 6 months later.  b) If ASCUS or worse, colposcopy (with endocervical curettage if unsatisfactory). If negative, repeat cytology at 12 months. If biopsy-proven condyloma/CIN 1, repeat cytology at 6 months. If CIN 2/3, perform DEP. |
| **Low-Grade Squamous Intraepithelial Lesion (LSIL)** | Repeat cytology at 6 months*  a) If normal, repeat cytology 6 months later.  b) If ASCUS or worse, colposcopy with endocervical curettage†. If negative or biopsy-proven CIN 1, repeat cytology at 6 months. If CIN 2/3, perform DEP.  If subject HIV sero-converts during the course of the study, she will be referred for colposcopy. |
| **High-Grade Squamous Intraepithelial Lesion (HSIL)** | Colposcopy with endocervical curettage  a) If satisfactory colposcopy: Rule out vaginal dysplasia as a source (with acetic acid and/or Lugol’s iodine). Review enrollment Pap, colposcopic findings and biopsies. If still suspicion for HSIL, perform DEP.  b) If unsatisfactory colposcopy, perform DEP.  c) If CIN 2 or 3, perform DEP. |
| **Atypical Glandular Cells of Undetermined Significance (AGUS)** | Colposcopy with endocervical curettage. Perform endometrial biopsy if age ≥40 years or abnormal bleeding at any age.  a) If negative or biopsy-proven condyloma/CIN 1, repeat cytology at 4, 8, and 12 months.  b) After 3 negative tests, repeat cytology at exit.  c) If AGUS recurs, perform cone biopsy. |
| **Adenocarcinoma in situ** | Colposcopy with endocervical curettage. If not invasive cancer, perform cone biopsy. |
| **Cancer** | Colposcopy with endocervical curettage. If not invasive cancer, perform cone biopsy. |

DEP indicates diagnostic excisional procedure (e.g., cone biopsy, loop electrosurgical excision procedure).

†Endocervical curretage should be performed if the colposcopy is unsatisfactory or no lesion is seen on colposcopy for Pap ASCUS or LSIL.

*At the second screening test, the threshold for colposcopic referral will be ASCUS.

## Protocol for Social Science Component of the MIRA Trial

**Acceptability and Feasibility of Diaphragm for Use in HIV Prevention and Trial Experience: A Qualitative Research Component of the MIRA Trial**

Prepared by:

Chiweni Chimbwete, Social Science Coordinator

This protocol summarizes the Qualitative Research Component (QRC) to be carried out alongside main MIRA trial activities in order to better understand the social context and processes of the trial. Specifically, this protocol contains:

- Aims and objectives of the QRC,
- QRC research and design methods,
- Plans for obtaining QRC informed consent and ensuring participant confidentiality,
- A summary of staff training required to conduct the QRC, and
- The plan for QRC data analysis.

**I. Aims and Objectives**

The primary objective of the QRC is to complement and supplement main trial activities by addressing two secondary objectives of the main trial (specified in the main MIRA Trial Protocol) that the QRC specifically aims to address are:

1. To assess the long-term acceptability of the diaphragm and lubricant gel for HIV prevention.
2. To examine the feasibility of the diaphragm and lubricant gel as a sustainable HIV prevention strategy.

These two objectives include factors such as:

- Individual attitudes toward and practices of diaphragm and gel use, including issues of stigma related to their use;
- Partner attitudes towards the diaphragm and gel, their involvement and awareness in decision making about the actual use of the products;
- Pregnancy and its relation to use of the diaphragm and gel; and
- Provider (trial clinicians and research staff) attitudes towards the diaphragm and logistical factors which emerge based on their experience in the trial.

The following is an additional objective of the QRC:

1. To study the experiences of trial participants by examining:

- Participant understanding and acceptability of the concepts and processes explained in the main trial informed consents (ICs) in order to improve the process and retention of IC information by participants over time;
- Stigma that may arise from women’s participation (or not) in the trial; and
- Social and community factors that may affect the conduct of the trial.

Finally, a fourth objective of the QRC is to use qualitative research techniques to explore the internal validity and sensitivity of outcomes of the main trial, through a process of triangulation. Triangulation is the use of multiple methods of data collection to answer a research question. Besides being a tool for testing validity of findings, triangulation also helps to ensure comprehensiveness of study measures by offering alternative interpretations of the data (Mays and Pope, 2000; Ulin et al, 2002). The use of various sources of data within the QRC will help to corroborate both main trial and QRC results. Any apparent contradictions in the results willl not be seen to undermine the results, but rather offer further scope for approaching the research problem from different perspectives (Barbour, 2002).

**II. Research Design and Methods**

Both quantitative and qualitative methods will be used to achieve the QRC aims and objectives; however, the focus will be mainly qualitative. The QRC will require data collection from multiple sources and involvement of both main trial and social science staff. This approach will provide a critical mass of expertise from different fields to conduct and interpret outcomes of the trial in a comprehensive manner (Vuckovic, 2002; Mays and Pope, 1995).

Specific plans for achieving the first 3three objectives listed above are provided below. The fourth objective will be addressed after data collection and analysis for the first 3 objectives are complete.

**Objective 1:** **Acceptability**

In order to explore further the social and behavioural factors surrounding acceptability of women-controlled use of the diaphragm and lubricant gel for STI/HIV prevention, focus group discussions (FGDs) will be conducted with different samples of participants at Months 3 and 12 of participation and after the final, or Month 24, visit. These women will also be asked to invite their partners to take part in FGDs with other men whose partners are in the trial The FGDs for both the study participants and their partners will focus on acceptability and feasibility of the diaphragm. Any interesting cases identified through FGDs, may be followed-up with in-depth interviews (IDIs).

Although participation in these FGDs will require discussion of personal experience with the use of study products, we think that FGDs will be a more effective first line measure than IDIs. First FGDs are a more cost effective method of data collection than IDIs, and second, prior to the MIRA trial, a diaphragm acceptability study in Zimbabwe found that women were more shy and reserved in IDIs than they were in FGDs.

In a trial of vaginal products among women and some of their male partners in western

Uganda found the proportion of women using the products secretly declined from 40 percent in the first week to 22 percent after 10 weeks (Green et al, 2001; Pool et al, 2000). A reason cited for this pattern of disclosure in the Ugandan study was that it was well known in the community who was participating in the study and women felt it right to inform their partners before they were informed by other community members (Green et al, 2001). For the MIRA QRC, we intend to interview participants and their partners at the end of the trial and ask about their experiences/knowledge of secret use and overt use of the diaphragm and gel.

**Objective 2: Feasibility**

The main trial Acceptability Form (AC) collects detailed information on both acceptability and feasibility of the diaphragm and gel as an HIV prevention method. Participants are asked about accessibility and affordability of the diaphragm and diaphragm hygiene preferences and practices. We intend to include questions on these issues in the FGDs and IDIs conducted for Objective 1.

In addition, we will also seek the perspective of trial staff regarding the feasibility of the diaphragm and gel for HIV prevention. We intend to use two methods of data collection to obtain this information from staff: semi-structured interviews (SSIs) with staff and observation/review of documentary evidence of staff experience.

Note that we intend to conduct FGDs for participants at Months 3, 12 and 24 of participation and that almost all IDIs will be conducted only at the end of trial participation (See Table 1). This is to assess changes in attitudes, behaviour, belief and practice throughout the trial duration. Green *et al.* (2001) noted that although proportions of women who reported secret use of vaginal products declined during the trial, in the post-trial period secrecy and control were some of the most important characteristics reported about the products. In our case, we are especially interested in knowing how acceptability of the diaphragm and gel differs during and post-trial.

**Objective 3: Experience with the trial**

Our ability to determine the effectiveness of the diaphragm and lubricant gel for HIV prevention will depend on several factors including whether study staff adhere to Standard Operation Procedures (SOPs) and whether participants comply with study procedures. Several of these factors can be addressed through regular monitoring visits and internal data validity checks. The focus of the third objective of the QRC is not to address adherence and compliance, but rather to evaluate the trial as a “yet-to-be known phenomenon, an experience in and of itself” (Sandelowski, 1996). This objective will be achieved using multiple methods of data collection with various groups of participants and staff. Methods and aims are outlined here (See Table 1 for timing details.):

- *Understanding of concepts and processes explained in the main trial ICs and acceptability of the informed consent process.*

A number of qualitative studies in randomized clinical trials in the UK have shown that although participants may have high levels of recall of study design terms and informed consent, they had difficulty in coming to terms with the concepts underlying the study design (Donovan et al., 2002; Featherstone and Donovan 1998; May and Pope, 1995; 2000; Mills et al, 2003).

To collect data regarding the Informed Consent process, women in the MIRA trial will be given an Informed Consent Quiz at the end of their Enrollment and Month 12 visits to assess their recall of concepts explained in the Enrollment Informed Consent. In addition to the IC Quiz, two FGDs per clinic may be conducted with study participants and include questions regarding how participants feel about the informed consent process and aspects of study design, such as randomization into the two study arms. The results of the IC Quiz and findings from any Informed Consent FGDs completed will be used to assess understanding of informed consent and place it in a cultural context. The in-depth interviews conducted with study participants at the end of the trial will also include questions regarding their experience of undergoing study procedures and further enhance our findings.

- *Experiences of screened out women and/or stigma that may arise from women’s participation (or not) in the trial.*

There are several ways in which stigma may be introduced or become a factor for individuals participating or seeking to participate in HIV prevention trials. First due to extremely high HIV prevalence in the regions where HIV prevention are generally conducted, a large number of women will be “screened out,” or deemed ineligible for participation, due to HIV positivity (and/or other factors). It has been noted that one of the greatest challenges for researchers in these clinical trials is to understand the impact of HIV testing on a woman, her family and community (Ramjee et al, 2000).

Second, the stigma associated with condom use in stable relationships in many parts of the world is well documented (e.g. Pool et al, 2000). Since they may not interfere with sexual intercourse, it is thought that women-initiated methods for protection against HIV and STI infection such as the diaphragm and gel offer hope to reduce some of this stigma. We hypothesise that there may be less stigma associated with the diaphragm in the context of marital relationships, compared to that associated with condoms.

To address both of these issues, questions about HIV testing-associated stigma both diaphragm and gel associated stigma *and* condom associated stigma will be included in the focus group discussions with participants and their partners conducted at the end of the trial.

We will also conduct semi-structured interviews with women who are screened out in order to better understand the reasons why women come for screening and how they feel about being screened out. We will ask them about how they heard about the study, why they decided to participate, and their experience with the screening process. It has been observed that there are a number of women who come for screening despite knowing that they are HIV-positive. Some participants have given various reasons for this and staff have observed different scenarios (for example to get some money and to confirm HIV-positive result).

Table 1: Proposed QRC Activities per MIRA Clinic

| Method | Target population | Objectives addressed | Number of FGDs, IDIs, *etc.* per clinic  and timing of each | | | | | |
| --- | --- | --- | --- | --- | --- | --- | --- | --- |
|  |  |  | SCR | ENR | M3 | M6 | M12 | M24 |
| FGD | Participants (B) | 3 |  |  | 2 |  | 2 |  |
| FGD | Participants (C) | 1,2 & 3 |  |  |  |  |  | 2 |
| FGD | Participants (D) | 1, 2 & 3 |  |  |  |  |  | 2 |
| FGD | Partners (D) | 1, 2 & 3 |  |  |  |  |  | 2 |
| SSI | Screened-out | 3 | 100 |  |  |  |  |  |
| SSI | Site staff | 2 & 3 |  |  |  | * |  | * |
| IC Quiz | Participants (B) | 3 (IC) |  | ** |  |  | ** |  |
| IDI  (optional) | Participants (C) | 1, 2 & 3 |  |  |  |  |  | 0 to 5 |
| IDI  (optional) | Participants (D) | 1, 2 & 3 |  |  |  |  |  | 0 to 5 |
| IDI  (optional) | Partners (D) | 1, 2 & 3 |  |  |  |  |  | 0 to 5 |

(D) = Diaphragm arm; (C) = Condom arm; (B) = Both arms

*1 SSI will be conducted per discipline at each clinic, *e.g.* 1 for the clinicians, 1 for the interviewers, *etc.*

****The IC Quiz is completed by all trial participants who have an Enrollment of M12 visit.

- *Social and community factors of the trial*.

To date, all MIRA trial sites have screened-out a sizable number of women due to HIV positivity. Nearly 300 women are expected to seroconvert during the trial. All sites currently document the HIV-focused clinical and social services available in the catchment area of the clinic. Women found to be HIV positive either at screening or who seroconvert during participation are referred to these services for support and assistance. The QRC will undertake to map these services in the trial communities (including where and how women get obstetric /gynaecological care and where women access condoms outside the trial) potentially to inform diaphragm and gel distribution efforts at trial end (if found effective). This information will be obtained from records maintained by staff.

**Recruitment and methods by participant type**

We provide here a summary of how various types of participants will be recruited for the qualitative studies.

### **Screened-out participants**

Interviews will be conducted with screened-out participants using a semi-structured interview guide that has been prepared to collect data on knowledge about the trial, recruitment criteria, interest in participation and feelings about being unable to join the trial. This method is preferred because it is quick and will not take too much of the participants’ time. It will be administered in a designated private space either just before the women leave the clinic or staff will record their contact details to invite them for an interview later. If a potential participant is screened out according to the specified criteria, the staff member doing the screening will ask the participant if she can consent to have a brief interview about her experience with the recruitment process at the clinic.

Screened-out participants will be identified as follows:

a) Screening visit:

-Participants who decline to give oral consent to complete the eligibility checklist.

-Participants who are eligible per EC but deemed ineligible per lab results i.e. HIV-positive, pregnant, or both. These will also include those who decline to give written screening consent.

-We can also interview potential participants who show interest in the study, come to the clinic and listen to an education talk. After that they screen themselves out and leave the clinic.

b) Enrollment visit (approximately 14 days after the screening visit):

-Participants will include those who may be screened out due to refusal of CT or GN treatment for STI and are confirmed sero-positive with an HIV Elisa test, and those who decline or are unable to give written consent for participation in the main study.

In total we hope to interview 100 participants per clinic. The coordinator will need to monitor the number of screened-out participants so as to assess the feasibility of interviewing all of them, or only a sample of them within that month. Options include interviewing women only on selected days of the month or interview every ith (e.g. second, third, fourth etc) woman who presents for screening. The site social science coordinator should keep careful documentation of the number of women screened out, approached for SSI participation, number who declined participation, and the number who accepted participation.

### **Participants in the trial**

Focus group discussions will be appropriate to capture the collective experiences of women participating in the trial. Topics such as how participants understand the informed consent process, stigma, inclusion/exclusion criteria, study design (e.g. randomization) and visit procedures will be discussed. The discussions will be moderated using pre-prepared guides.

Some participants maybe familiar with each other and the trial staff to varying degrees; the facilitator will assess if this impacts on the conduct of the focus group discussion. During the consent process for FGDs we will make sure that women understand that by participating in the FGD they will make it known to other people that they are in the trial and that other participants will know their name and answers to any questions discussed.

We will use the site’s MIRA Trial Study Register (or a CIDEA report) to identify a cohort that has had their Month (M)3/Month12 visit within the last three calendar months, *e.g.* in April we can target all women who just had their M3/M12 visit between January and March. We will then compile a list of all the women identified and their contact details. There will be one list for M3 women and another for M12. Between 24 and 48 women will participate in a group discussion at each study clinic; half at M3 and half at M12. Specifically, two FGDs (conducted three months apart) each consisting of between 6-12 participants will be held at each clinic visit for M3 participants. The same will be done for M12 participants. It is recommended that only one FGD takes place per day so as to allow for immediate debriefing and transcription.

- Participants who complete trial participation

As stated above, the QRC will include FGDs and possibly IDIs with participants who have completed 24 months of participation. Specifically, we expect to conduct two FGDS with participants in the condom arm and two FGDs with participants in the D&G arm per clinic. . The target number of participants for each FGD is 6-12, and these will be conducted three months apart.

a) **FGDs:** Trial participants in both study arms who successfully complete the trial cycle as per the minimum and maximum number of visits (18-24 months) will be identified. Interviewers will conduct exit interviews with women during their final clinic visit to ask if they wish to be contacted for follow up qualitative interviews after the trial is over. One-two weeks before the proposed FGD, we will use the exit interview records and the sites MIRA Trial Study Register (or a list from CIDEA) to identify women who had their final study visit within three months before the proposed FGD date. Condom and D&G arm women will be listed separately.

Questions such as: motivating factors for remaining in the study until the end; sexual experience while using study products; overt and covert use of study products; the ACASI experience; and the likelihood of future use of study products will be discussed in these FGDs.

b) **IDIs:** We will possibly identify up to 5 women per clinic, with different experiences (consistent or inconsistent users of trial products) for follow-up in individual in-depth interviews. These will be identified in the FGDs or using ACASI data.

### **Partners of women in D&G arm who complete the trial**

As stated above, the QRC also will include FGDs and possibly IDIs with partners of women in the D&G arm. We expect to conduct two FGDs with partners of participants randomised in the D&G arm at each clinic after the participants’ final visits. In the Exit Interview Form participants in the D&G arm will be asked if we can contact their partner or if they can ask him if we can invite him to take part in qualitative interviews. Again, the target number of partners for each FGD is 6-12, and these will be conducted three months apart. In total we expect to interview a total of 12-24 partners of D&G users at each clinic site.

### **Qualitative data from staff**

When interacting with trial participants and other stakeholders in the community, study staff document their experiences and observations. This documentation will be collected and analyzed to get a preliminary idea of what potential providers might think and provide information on the social context of the trial. As stated above, a variety of methods will be used to collect information from staff:

**i) Interviews with site staff** will be done using a prepared semi-structured interview guide. One set of SSIs was conducted in the early 2004; the other set will be conducted at the end of the trial. Staff performing different functions in the trial will be identified for interviews. They include clinicians, counselors, recruiters, educators and coordinators. The selection will be based on a convenience sample according to the work schedule of staff. An external interviewer will approach staff ahead of time to give them an opportunity to determine when and how much time they can give for interview.

They will be 5-6 SSIs with trial staff per clinic to represent function groups.

**ii)** **Documentary evidence of staff experience** from routine chart notes, staff reports, minutes of regular staff meetings, educational material and media stories at the sites, monitoring reports and database of services will be analyzed. This source also includes minutes of meetings involving the wider MIRA group. This information will help to match what people say they do (in the interviews and discussions) and what they actually do based on trial outcomes.

**iii) Documentation of interactions with stakeholders** will also be reviewed. Trial staff keep updated records of community representatives and stakeholders associated with the MIRA trial and their interactions with these groups/individuals. In their interactions with the stakeholders, trial staff may learn about their views on the feasibility of the diaphragm to protect women against HIV and STIs, and these observations may be recorded in site documentation. All this information can potentially provide important contextual information for trial outcomes and also bring useful insight into what participants say they do and the social reality of what actually takes place.

**III. Informed Consent and Participant Confidentiality**

Separate written informed consent will be obtained from participants each time we hold an interview with them (*i.e.* FGD, IDI, *etc.*). For screened-out participants the interviewer will ask for verbal informed consent before commencing the interview. The moderator of each interview will ensure confidentiality.

We will stress to all participants that they are free to decline participation in the qualitative study and that this will not in any way affect their participation in the main trial.

**IV. Staff training**

The Social Science Coordinator will train all interviewers who will be collecting data for the qualitative research component. The QRC training focuses on qualitative data collection methods such as IDIs and FGDs. During the training methods for selecting the participants, obtaining consent and general methods for conducting the interviews are discussed. Prepared interview guides are also discussed. These guides have been developed based on MIRA trial CRF questions, items used in previous studies, and newly created questions, appropriate for this work.

The staff training offers an opportunity for validation of these guides, and after training the guides will be pilot tested before used with study participants.

**V. Qualitative data analysis**

Qualitative data analysis is an iterative process that continues from the design stage to the reporting of research results. The Social Science Coordinator for MIRA will give site staff as much input as possible in the administration of the QRC protocol. We also recognise the fact that the researcher collecting the data plays a crucial role in the analysis of qualitative data. Ibis Reproductive Health will only play a coordinating and supporting role to the site staff. However, there is need for constant liaising between site staff, the individuals interested in a research topic from the MIRA group and Ibis in the analysis of data.

Research staff in MIRA will interview in the site language (*i.e.* isiZulu, Sesotho or Shona), tape record, transcribe and translate the interviews into English. They will then present these in a form ready for thematic analysis to interested scientists in the larger group. The data will be analysed using Atlas/ti, qualitative analysis software which will be made available to the site. The selection of Atlas was based on considerations such as availability of local expertise, backup for training and common research goals in the larger MIRA trial group. It will also enable us to use the constant comparison method which enables the comparison of themes by various categories of the data (e.g. Donovan et al, 2000; Pope et al, 2002). The thematic analysis will be conducted according to guidelines developed for the interviews and observations that will be uniform across sites to ensure that the same factors are studied in different settings.

It is also recommended that more than one person code and interpret the qualitative data. In each case there will be two social scientists that will code and interpret selected transcripts and compare their interpretations. However, if only one person interprets the data without the benefit of multiple coders, the person needs to be skilled and be an experienced researcher (Dingwall et al, 1998). The researcher should keep detailed records of the analysis process to show how one interprets the data and reaches conclusions. This will create an ‘audit trail’ and ensure that the data analysis is rigorous, and will enable multiple reviewers to conduct independent analyses and compare the findings (Dingwall et al, 1998; Ulin et al, 2002). Summaries of results will be presented by themes and main findings noted to be compared across sites.

**References**

1. Barbour R. (2001). Checklists for improving rigour in qualitative research: a case of the tail wagging the dog? *British Medical Journal.*  322: 1115-7.
2. Donovan J, Mills, N, Smith M, Brindle, L et al. (2002). Improving design and conduct of randomized trials by embedding them in qualitative research: ProtecT (prostate testing for cancer and treatment) study. *British Medical Journal.*  325: 766-70.
3. Dingwall R, Murphy E, Watson P, Greatbatch D, Parker S. (1998). Catching goldfish: quality in qualitative research. *Journal of Health Services Research and Policy.*  3:167-72.
4. Featherstone K, J. Donovan. (1998). Random allocation or allocation at random? Patients’ perspectives of participation in a randomized controlled trial. *British Medical journal.* 317: 1177-80.
5. Green G, R. Pool, S. Harrison, G. Hart, J. Wilkinson, S. Nyanzi and J. Whitworth (2001). Female control of sexuality: illusion or reality? Use of vaginal products in south west Uganda. *Social Science and Medicine*: 52:585-598.
6. Mays N, C. Pope. (1995). Qualitative research: Rigour and Qualitative research. *British Medical Journal.* 311: 109-112.
7. Mays N, C. Pope. (1995). Qualitative research: observational methods in health care settings. *British Medical Journal.* 311: 182-184.
8. Mays N, C Pope. (2000).Assessing quality in qualitative research. *British* *Medical Journal.* 320: 50-2.
9. Meyer J. (2000). Using qualitative methods in health related action research. *British* *Medical Journal*. 320: 178-81.
10. Mills N, J Donovan, M Smith, A Jacoby, D Neal, F Hamdy. (2003). Perceptions of equipoise are crucial to trial participation: a qualitative study of men in the ProtecT study. *Controlled Clinical Trials*. 24: 272-282.
11. Pool R, J. Whitworth, G. Green, A. Mbonye, S. Harrison, J. Wilkson and G. Hart (2000). An acceptability study of controlled methods of protection against HIV and STDs in southwestern Uganda. *International Journal of STD & AIDS:* 11:162-167.
12. Pope C, N Mays. (1995). Qualitative research: Reaching the parts other methods cannot reach: an introduction to qualitative methods in health and health services research. *British Medical Journal*. 311: 42-45.
13. Pope C, S Ziebland, N Mays. (2000). Qualitative research in health care: Analysing qualitative data. *British Medical Journal*. 320:114-6.
14. Ramjee G, N. Morar, M. Alary, L. Mukenge-Tshibaka, B. Vuylsteke, V. Ettiègne-Traoré, V. Chandeying, S. Karim and L. Van Damme on behalf of COL 1492 Study Group. (2000). Challenges in the conduct of vaginal microbicide effectiveness trials in the developing world. *AIDS:* 14:2553-2557.
15. Sandelowski M. (1996). Using qualitative methods in intervention studies. *Research in Nursing and Health.* 19: 359-364.
16. Vuckovic N. (2002). Integrating qualitative methods in randomized controlled trials: The experience of the Oregon centre for complementary and alternative medicine. *Alternative and Complementary Medicine.* 8 (3):225-227.
17. Ulin P.R., E. T. Robinson, E.E. Tolley, E.T. McNeill. (2002). Qualitative Methods: Applied Research in Sexual and Reproductive Health. Family Health International, North Carolina

## Protocol for HSV-2 Sub-Study

**Detection and Characterization of Recent HSV Infection by HSV Avidity Index in Soweto**

**Principal Investigator:** Guy de Bruyn, MBBCh, MPH, Perinatal HIV Research Unit

**Co-investigators:** Glenda Gray and James McIntyre, Perinatal HIV Research Unit

**Background**

Herpes simplex virus is the commonest cause of genital ulcer globally ^66-69^. Both genital ulcers and HSV specifically have been associated with HIV transmission. From multiple studies, the presence of HSV is associated with a relative risk of HIV acquisition of 2.1 compared with HSV negative persons ^70^. Furthermore, it has been observed that persons with recent HSV infection appear to have an even greater elevation in the risk of HIV acquisition than persons with more remote HSV infection ^71^. It is therefore of interest to characterize the recency distribution of HSV infection in order to further understand the impact of recent and remote HSV infection on HIV acquisition.

In the context of the MIRA study, HSV infection will be determined by serologic reactivity in the Focus Elisa test ^72^. A recently published modification of the test procedure allows for reactive specimens to be further characterized in terms of the avidity of HSV antibodies ^73^. The immune response to infection with HSV and other pathogens is associated with increased avidity of antibodies over time ^74-78^. By correlating avidity index scores among persons with known timing of infection, the time course of avidity index has been modelled, and proposed cut-off scores have been tested ^73^.

**Objectives**

The specific objectives of the study are:

1. Determine the prevalence of recent HSV infection in a cohort of HIV uninfected women in Soweto as measured by avidity index score ≤40
2. Describe the distribution of avidity index scores for HSV seropositive women in Soweto
3. Describe the relationship between baseline serologic reactivity with the Focus HSV Elisa and avidity index

**Research design**

This ancillary study is a pilot/feasibility laboratory study. We propose to use stored serum specimens from the baseline visit of participants enrolling in the MIRA trial at PHRU. Laboratory personnel will identify women who had a positive test for HSV-2 in the enrolment sample. Residual serum samples from these participants will be retrieved from storage. The available samples will be thawed and testing repeated in duplicate with the modified assay according to the published method ^73^. An avidity index value for the enrolment sample will be determined from the paired results, by dividing the treated OD value by the untreated OD value and multiplying by 100.

**Study population**

Participants in this ancillary study will be women who have consented and enrolled in the MIRA study, who tested positive for HSV-2 in the enrolment sample, and who have stored aliquots of serum available at Contract Laboratory Services from the enrolment sample.

**Analysis**

For the first study objective, assuming that 200, 300, or 550 samples were available for testing from the participants enrolled to date and found to be HSV seropositive, the confidence limits for the given prevalence were calculated by exact methods for binomial proportions (Table).

The second and third objectives would be presented with simple descriptive statistics.

|  | N = 550 | | N = 300 | | N = 200 | |
| --- | --- | --- | --- | --- | --- | --- |
| Prevalence | **LCL** | **UCL** | **LCL** | **UCL** | **LCL** | **UCL** |
| 0.50 | 0.4574 | 0.5426 | 0.4420 | 0.5580 | 0.4287 | 0.5713 |
| 0.10 | 0.0762 | 0.1282 | 0.0685 | 0.1397 | 0.0622 | 0.1502 |
| 0.05 | 0.0341 | 0.0727 | 0.0283 | 0.0811 | 0.0242 | 0.0900 |
| 0.01 | 0.0400 | 0.0236 | 0.0021 | 0.0289 | 0.0012 | 0.0357 |
| 0.005 | 0.0011 | 0.0159 | 0.0008 | 0.0239 | 0.0001 | 0.0275 |
| 0.001 | <0.0001 | 0.0101 | - | 0.0122٭ | - | 0.0183٭ |

LCL, lower confidence limit; UCL, upper confidence limit; ٭1-sided, 97.5% confidence bound

Development of the assay had validated a threshold avidity index value of 40 to define recent infection. The sensitivity and specificity of this rule for samples drawn within six weeks of initial onset of HSV-2 was 81% and 86%, respectively. As the likelihood of observing one such event in a cohort is unknown, but likely to be small, we propose to test 550 baseline specimens to optimize the chances of observing a recent seroconversion.

Data would be collected and entered in a separate database, with samples identified by study number. Data analysis would be undertaken by a PHRU statistician.

**Ethical considerations**

All volunteers are requested to provide consent for storage of specimens at enrolment. The research proposed in this ancillary study would be covered by the language contained in the storage consent document. No additional approval would be required from the local Institutional Review Board for this research. However, the IRB would need to be informed of the proposed research.

**References**

## Protocol for Managing Seroconverters in the MIRA Trial

*English Version 4.0, Revised January 23, 2007*

***Table of Contents***

[**1** **Introduction to the Standard of Care (SOC)** 83](#__RefHeading___Toc127263374)

[**2** **Goals** 84](#__RefHeading___Toc127263375)

[**3** **Eligibility Requirements** 84](#__RefHeading___Toc127263376)

[**4** **Duration of Services** 84](#__RefHeading___Toc127263377)

[**5** **Services Offered** 84](#__RefHeading___Toc127263378)

[**6** **Study Sites and Referral Organizations** 86](#__RefHeading___Toc127263385)

[**7** **Study Procedures** 88](#__RefHeading___Toc127263391)

[**8** **Data Collection, Management, and Analysis** 90](#__RefHeading___Toc127263398)

[**9** **Human Subjects Consideration** 91](#__RefHeading___Toc127263402)

***Introduction to the Standard of Care (SOC)***

The investigators of the Methods for Improving Reproductive Health in Africa (MIRA) trial have established a Standard of Care (SOC) package for HIV-positive women who seroconvert during their participation in a randomized, clinical trial to determine whether the diaphragm with Replens gel prevents HIV acquisition in women. The provision of such a care package for MIRA research participants comes in response to a policy proposal by the Gates Foundation and a need to address the ethical challenges presented by HIV prevention trials for which clear guidelines do not exist.

Currently, the governments of Zimbabwe and South Africa are implementing programs for the distribution of antiretroviral (ARV) therapy as well as other care services for people living with HIV/AIDS (PLWHA). Recently, antiretroviral drugs have become more affordable and therefore more accessible to African countries that previously had very limited access. Because antiretroviral treatment can greatly affect the quality of life and longevity of people living with HIV/ AIDS (PLWHA), it is critical to deliver it to those in need. Despite efforts by the governments of Zimbabwe and South Africa to deliver treatment, each country is faced by challenges that translate into original treatment targets not being fully met. As these government programs improve their systems to deliver ARVs and other related services, clinical trials such as MIRA have an opportunity to fill in the gaps of HIV-related services and care when government resources fall short.

The investigators of the MIRA trial propose a four-component policy of care and treatment to participants who are identified as HIV-positive during the trial. The four main components include 1) Psychosocial support and counseling, 2) Monitoring of disease progression using CD4 counts, 3) Screening for opportunistic infections, and 4) Access to ARV therapy.

Women who are HIV negative at screening, enroll into the study and are then found to be positive at subsequent follow-up visits (seroconverters) will be offered these services on an on-going basis throughout the life of the trial, currently scheduled to end in May 2007. Care and services at each of the three MIRA sites (Harare, Zimbabwe; Durban, South Africa; Johannesburg, South Africa) may vary, depending on the availability of outside resources in each area. To ensure the quality of care and distribution of services and treatment, systems of monitoring and evaluation will be implemented at each site.

***Goals***

The two goals of the Standard of Care (SOC) component of the MIRA trial are:

- To inform and educate women who seroconvert during their participation in the MIRA trial about available local services that may monitor their health, HIV infection, and emotional wellbeing.
- To encourage and assist these women to access these services during their participation with MIRA, which will in turn help to ensure a sustained relationship with these providers when the MIRA trial ends.

***Eligibility Requirements***

All MIRA seroconverters are eligible for SOC services. HIV testing is done at every quarterly scheduled visit in the MIRA trial. HIV status is confirmed by ELISA, or other tests as needed, after a participant receives two positive rapid HIV test results.

***Duration of Services***

SOC services are available to a MIRA participant as soon as she seroconverts. Women may participate in the SOC services throughout the life of the MIRA trial, currently scheduled to end in May 2007. SOC services may be accessed while the woman is still active in the MIRA trial and may continue after she completes her participation in the main trial activities and exits the study. Before the trial ends (out of the field in May 2007), study staff will ensure to the greatest extent possible that seroconverters are enrolled or networked into a system of care independent of MIRA. Once the MIRA trial ends, it will not be able to continue to provide payment for HIV-related care or services.

***Services Offered***

In addition to the counseling and health services provided during their participation in the MIRA trial, all MIRA seroconverters are eligible for Standard of Care (SOC) services until the end of the MIRA Trial (approximately May 2007).

The SOC package includes the following four components, which are explained further in the sections below:

1. Psychosocial support and counseling
2. Monitoring of disease progression using CD4 counts
3. Screening for opportunistic infections
4. Access to ARV therapy.
5. **Psychosocial Support Services and Counseling**

Women who seroconvert during the trial have special psychosocial needs. Two to three follow-up post-test counseling sessions are scheduled with HIV-positive participants when their seroconversion is confirmed, depending on participant need. Regular counseling sessions then occur every three months as part of participants’ scheduled MIRA visits. After their participation in MIRA ends, ongoing counseling opportunities with MIRA staff will be available every three months, or sooner if needed. SOC participants also may wish to receive additional psychosocial support beyond the counseling provided at the MIRA clinics, which will be encouraged and facilitated by MIRA staff. Such support may include referrals to one-on-one therapy, support groups, legal aid, health/nutrition information, advocacy, and more. MIRA staff will give SOC participants referral letters to assist them in accessing these local resources.

1. **Monitoring of disease progression using CD4 counts**

Because the study sites have access to different local resources, the two South African sites will rely primarily on referrals for care while the Zimbabwe site will do some procedures at the study clinic in addition to using referrals for other care.

For monitoring disease progression, CD4 counts will be the standard among all three sites. However, some sites will have the ability to run other tests as part of the care given through government and organizational programs. UZ-UCSF is able to perform CD4 counts, full blood counts (FBC), urea & electrolyte (U&E) and a liver function test (LFT) on specimens collected at the MIRA clinic. If a participant consents to the additional procedures in the SOC package, the first testing should occur as soon after seroconversion as possible, CD4 and full blood counts should be repeated every six months, and the U&E and/or LFT may be repeated, as necessary. MRC will perform CD4 tests, and other HIV-monitoring tests, as necessary, at the MIRA clinics upon consenting to the SOC package, and every six-months thereafter, until the participant closes out of the MIRA trial. After the trial, MRC will offer CD4 testing through MIRA labs or at referral sites. PHRU will refer participants for CD4 testing and other HIV-monitoring tests, as necessary, which will be repeated every six months until the end of the MIRA trial.

1. **Opportunistic Infection Screening and Treatment**

While women are still actively participating in the MIRA trial, they will continue to receive physical exams as needed and during their closing MIRA visit. Per MIRA protocol, free treatment for any reproductive or urinary tract infections will be provided. After exiting the MIRA trial, UZ-UCSF SOC participants will continue to be tested and treated (as needed) for Chlamydia, gonorrhea, trichomoniasis and syphilis every six months at the study clinic. After their participation ends in the main trial activities, participants at the MRC and PHRU sites will be referred for all clinical care and testing.

SOC participants at all sites will be referred to outside facilities for opportunistic infection (OI) screening. At the OI clinics, participants will receive physical exams, including screening for anemia, TB (chest X-rays and sputum examinations, if medically indicated), pneumonia, and any other clinical conditions requiring attention. Treatment will be initiated if appropriate. MIRA referral letters will be given to the participant to facilitate the transition to these outside facilities.

1. **Anti-Retroviral (ARV) Therapy**

SOC participants will be referred to governmental or other organizations and/or research studies that provide anti-retroviral (ARV) therapy when they seroconvert. If pregnant, women will also be referred to organizations or clinics providing ARV therapy for prevention of mother-to-child transmission. If a woman becomes eligible for ARV therapy but does not have access to treatment due to waiting lists at local programs, she may be evaluated and seek assistance and treatment through the resources of the MIRA trial until she is accepted into a local program and while the MIRA trial is still ongoing.

Participants will be evaluated by qualified clinicians working in the OI and other referral clinics to determine if they are eligible to begin ARV treatment. This clinical evaluation, combined with the results of laboratory tests, will be used to determine whether the participant should initiate ARVs, per national guidelines.

1. **Guidelines for Initiating ARV Therapy in Zimbabwe**

WHO guidelines for ARV therapy[[3]](#endnote-2)[[4]](#endnote-3) recommend that adults begin ARV therapy when they meet one of the following conditions:

1. Clinically advanced HIV disease defined by:
   1. WHO stage IV HIV disease, irrespective of CD4 cell count, or
   2. WHO stage III disease with a CD4 count <350/mm^3^
2. WHO stage I or II HIV disease with CD4 counts <200/mm^3^

Definitions of WHO stages of HIV disease may be found in the Appendix.

1. **Guidelines for Initiating ARV Therapy in South Africa**

In 2004, South Africa’s National Department of Health established national antiretroviral treatment guidelines.[[5]](#endnote-4) Under these guidelines, persons identified as HIV-positive will receive prevention counseling; screening and treatment of TB and other OIs; screening for pregnancy; introduction to HIV services; and CD4 counts to determine disease stage based on WHO criteria. Those in WHO stage I and II will also receive nutrition and psychosocial support.

Initiation of ARV therapy for adults are recommended under the following conditions:

1. CD4 counts <200/mm^3^, irrespective of HIV disease stage; or
2. WHO stage IV HIV disease, irrespective of CD4 counts *and* when the patient expressed the willingness and readiness to adhere to ARV treatment schedules.

Those receiving ARV treatment will undergo intensive adherence counseling. Palliative care will be sought for those who experience repeated treatment failure.

***Study Sites and Referral Organizations***

1. **Study Sites**

The SOC sites are the same as the MIRA trial sites: The Medical Research Council (MRC), with sites in Umkomaas and Botha’s Hill regions outside Durban, South Africa; Perinatal HIV Research Unit (PHRU), which is based at the Chris Hani Baragwanath Hospital in Johannesburg, South Africa; and the UZ-UCSF Collaborative Research Programme, with MIRA clinics in Chitungwiza and Epworth areas outside Harare, Zimbabwe.

1. **Referral Organizations**
2. **Medical Research Council (MRC)**

MRC has a large network of partner facilities around Durban, South Africa, that will act as referral organizations for SOC participants. In central Durban, McCord Hospital includes two clinics for voluntary counseling and testing (VCT) and screening. Provincial facilities include:

- Addington Hospital, which has an HIV and AIDS resource center (including a VCT clinic), a prevention of mother-to-child transmission (PMTCT) program, and an ARV clinic.
- CJ Crookes Hospital, which has an HIV Clinic, VCT clinic, and an ARV clinic.
- Prince Mshiyeni Hospital (Umlazi), which has an HIV clinic, VCT clinic, and PMTCT clinic.
- R K Khan Hospital, which has an HIV clinic and VCT clinic.
- The Valley Trust, one of South Africa’s oldest and best-known NGOs.
- St. Mary's Hospital, a Catholic Mission Hospital.

For psychosocial support referrals, MRC has partnered with local non-government organizations (NGOs), community-based organizations (CBOs), and other HIV prevention organizations, including McCord’s Sinikithemba Support Group, to build an ongoing psychosocial care support for SOC participants.

1. **Perinatal HIV Research Unit (PHRU)**

The MIRA site of the Perinatal HIV Research Unit (PHRU) is located at the Witswatersrand Academic Hospital, the Chris Hani Baragwanath (CHB) Hospital, in Soweto, Johannesburg. CHB was part of the first wave of government ARV rollout sites in South Africa, and is a PEPFAR recipient. Different sections of this large facility will serve as referral sites for opportunistic infections and ARV therapy.

HIVSA, PHRU’s sister organization, is the NGO providing the psychosocial peer support for the site’s SOC participants. A registered non-profit organization set up to provide support to the women, men and children who are currently or were participating in the PHRU research activities, HIVSA includes services in VCT, wellness, TB treatment, ARV for adults and children, and palliative care run as operational research.

1. **UZ-UCSF Collaborative Research Programme**

Three local opportunistic infection (OI) clinics will be utilized by the UZ-UCSF MIRA site: Parirenyatwa Hospital, operated by the Department of Medicine at the University of Zimbabwe Medical School; Harare Central Hospital in Harare; and Chitungwiza General Hospital, located in a peri-urban suburb 30 km south of Harare.

The UZ-UCSF Programme networks with a variety of support organizations in Harare and in the research communities. The MIRA Senior Counselor has evaluated these organizations and maintains current contact information for each.

***Study Procedures***

1. **Scheduled Visit Summary**

SOC visits initially work in tandem with main MIRA trial visits, and then add additional visits to accommodate the special needs of seroconverters and to continue the participant’s access to services after closing out of MIRA. Seroconverters who have already exited the study will be contacted and asked to return to the clinic to be given the opportunity to participate in SOC services.

There are five types of scheduled visits that SOC participants will experience, and each is described in more detail in the sections below:

- Seroconversion visit
- 2-week confirmatory test results/supportive counseling visit
- MIRA Trial follow-up visit(s)
- Post-trial bi-annual follow-up visit(s)
- Post-trial results notification visit(s)

For each visit described below, only the procedures specifically for seroconverters, or those performed in addition to the regular MIRA trial visits for the SOC, are noted. For the procedures of the regular MIRA quarterly follow-up visits, please see the MIRA Protocol, version 8.0.

1. **Seroconversion Visit**

If a MIRA participant is found to be HIV positive based on her rapid test results at a quarterly

follow-up visit, she will receive immediate post-test counseling. Confirmatory tests will be requested from the laboratory, and the participant will be scheduled for a visit to receive her results approximately 1-2 weeks from the date of the seroconversion visit. At the seroconversion visit, the counselors will give an introduction to the SOC services but will provide further details at her next visit, after her confirmatory results return.

1. **2-week Confirmatory Test Results/ Supportive Counseling Visit**

At this visit, the participant will learn the results of her ELISA confirmatory HIV tests and receive supportive counseling. This visit also provides the opportunity to explain the services, including the SOC, available to the participant at a time where she may be more capable of comprehending her options. If the participant misses this visit, staff will follow MIRA outreach guidelines, which begin by sending her a reminder letter. This letter will not disclose her status or in any way identify her as a seroconverter. A further supportive counseling visit may be scheduled if the counselor feels it is appropriate for the participant.

If a participant is confirmed as HIV-positive, the participant will receive ample post-test counseling and information about positive living, HIV transmission, diet/nutrition, and other relevant topics. The counselor will ask the participant if she would like to hear about the SOC services at this time and if so, will review with her with the SOC Information Sheet. The counselor will ensure that the participant understands the information and that the MIRA trial can only provide these health services, including ARV treatment (if needed), at no cost to her through the end of the MIRA trial (approximately May 2007). Study staff will help every seroconverter to establish a network of care independent of MIRA before the trial ends.

Written Informed Consent: If the participant is interested in taking part of the SOC services, the SOC written informed consent form will be administered. The counselor will explain that we seek her consent for the following procedures:

- Blood and urine specimen collection for performing CD4 and full blood counts, urea and electrolyte (U&E) and liver function tests (LFT).
- STI and pregnancy testing at post-trial bi-annual follow-up visits (or more frequently, if needed), Pap smears on an annual basis, and physical exams, only if clinically indicated. The results of these will be tracked in the participant’s file and used to monitor her health.
- Recording lab results from the above tests, and results from other clinics, in the participant’s MIRA file with her MIRA ID, and no other identifying information.
- Review of medical records from outside organizations.

Staff will remind her that she can still access SOC services, including referral services, without signing the informed consent. The participant will have an option to consent or not to having specimens taken for HIV-monitoring tests mentioned above. She will also have the option to consent or not to having test results recorded for study purposes. Finally, she will have the option to allow the MIRA staff to contact her family to request medical records and hospital notes, in the event of her death.

Once the participant is aware of the referral services available to her, she should be told about the additional counseling and support services outside of the MIRA clinic. Referral letters will be provided to assist her in accessing these services if she is interested.

1. **MIRA Trial Follow-up Visit(s)**

The participant should continue her scheduled MIRA Trial follow-up visits unless she or a clinician decides to discontinue her from the study. Counseling the participant about positive living is the centerpiece of every seroconverter’s follow-up visit. Positive pregnancy results will be given with full explanation of the options available to her in her community, including prevention of mother-to-child transmission services. Positive STI results will be disclosed with a thorough explanation of the heightened risk of transmission associated with STIs.

If a seroconversion is identified, and a participant consents to additional lab testing, specimen collection should be arranged as soon as possible. This may be at the supportive counseling visit, at an unscheduled visit, or at the next regularly scheduled visit. Specimen collection for CD4 counts will be performed at study clinics or by referral organization, depending on site specifications.

1. **Post-trial Bi-annual Follow-up Visit(s)**

The purpose of the bi-annual follow-up visit (or more frequent as needed) is to 1) offer supportive counseling to the participant and her partner (if requested); 2) collect specimen for CD4 counts and other blood and urine tests to monitor the participant’s disease progression; 3) test for pregnancy and sexually transmitted infections; and 4) offer referral for OI screening; 5) offer referrals to other services as needed. Women will only receive a pelvic exam at a follow-up visit when it is clinically indicated.

Bi-annual follow-up visits will occur at the MIRA clinic at UZ-UCSF, and at referral organizations at MRC and PHRU. The MIRA team proposes to implement an invoicing system to track general utilization of referral services.

If the participant misses a scheduled bi-annual follow-up visit, staff may send a reminder letter that will not disclose her status or in any way identify her as a seroconverter. However, there is no requirement to do home visits or track her as during the study.

1. **Results Notification Visit(s)**

Participants who provide blood and urine samples for testing will be scheduled for a results notification visit 1-2 weeks later. At this visit, test results, including HIV monitoring indicators, should be explained to the participant. Post-test counseling, if needed, will be provided, as will treatment if RTI/STI positive. If the participant misses a scheduled results notification visit, staff should send a reminder letter. This letter should not disclose her status or in any way identify her as a seroconverter.

***Data Collection, Management, and Analysis***

1. **Data Collection and Analysis**

Since the SOC is fundamentally a service package and not a research study, data collection is not its primary purpose. However, sites are seeking IRB and participant approval to collect minimal data (e.g., surveillance data) that is obtained in the process of providing care. CD4 and full blood count, as well as U&E and LFT, results will be recorded when they are completed if the participant has granted consent for data collection. In addition, STI, pregnancy and Pap smear data will be recorded. Frequencies of referrals given to participants and utilized will be recorded by each clinic so that the uptake of services may be reported to the funder. We propose to do a descriptive analysis on baseline CD4 counts at seroconversion and rate of disease progression (timing to qualifying for ARV therapy).

1. **Case Report Forms (CRFs) and Source Documents**

The MIRA Standard of Care SOC Form is the only CRF used in the SOC. This form will record the results of blood and urine tests, as well as Pap smear results, and will be transmitted via DataFax to UCSF’s Center for International Data Evaluation and Analysis (CIDEA). With the participant’s consent, test results from outside facilities will also be recorded on the CRF. If a participant does not provide consent for data collection, test results will be indicated on chart notes only, and no results will be collected on the CRF. Referrals will be tracked on a log in each SOC participant’s binder. Draft examples of each of these documents can be found in the Appendix.

1. **Referral Letters**

MIRA clinics will develop referral letters that will facilitate participants’ transitions to outside services. Depending on the requirements of different agencies, the referral letters may contain the participant’s name, National ID number and the date when it was issued by the MIRA trial, but will not include her MIRA ID number. The letter will feature a perforated bottom section that the referral organization will tear off and return to the MIRA clinic. This slip will indicate that a service was used at the organization and it will not include participant names, MIRA ID numbers or health information.

Please note that if an organization requires proof of HIV status and/or other lab results, the participant will be given these results separately from her referral letter to deliver to the organization.

***Human Subjects Consideration***

1. **Institutional Review Board (IRB) Review**

We will seek approval of this protocol from the Committee on Human Research at UCSF prior to commencing any procedure described herein. We will ensure that all procedures conform to U.S., South African and Zimbabwean ethical standards regarding research involving human subjects.

1. **Informed Consent**

Eligible MIRA Trial participants who accept the SOC services will be asked to review and sign a site-specific informed consent in the presence of a MIRA staff member and a witness. Since each site is approaching in-house procedures, referral services, and data collection in a unique way, the informed consent will differ according to the sites activities.

1. **Compensation**

Participant compensation for completing regularly scheduled MIRA visits remains as defined in the MIRA Protocol, version 8.0. Seroconverters who accept SOC services will receive transport reimbursement for attending any scheduled SOC visit at the MIRA clinic after discontinuation from the Main Study, which are as follows:

- Bi-annual follow-up visit (s)
- Results notification visit (s)

A transport reimbursement amount in local currency will be adjusted regularly to reflect the current equivalent value of not more than US$5. Unscheduled or interim visits, or visits to off-site referral agencies, are not eligible for transport reimbursement. Site managers may approve exceptions to this reimbursement policy on a case-by-case basis, as needed.

1. **Payment for Services**

The MIRA trial will pay for all SOC services, including ARV treatment (if needed), until the MIRA trial ends (approximately May 2007).

1. **Benefits and Risks**

This care package offers numerous benefits. Through these services, MIRA staff will help to monitor participants’ health, HIV infection, and emotional wellbeing. Participants’ disease progression will be tracked through CD4 counts and other blood and urine tests. Participants will be referred to services and treatment studies available in their community, including opportunistic infection screening and treatment and ARV therapy, if they become eligible. If the participant is or becomes pregnant, she will be referred to organizations that provide ARV therapy to help prevent maternal-to-child HIV transmission. Numerous counseling and other psychosocial support services will be offered to participants to help them adjust to life as an HIV-positive individual.

While the study staff will ensure participants’ confidentiality at the clinic, it is possible that other people may discover SOC participants’ HIV status if they find out that they are participating in this care package. Also, the care package as described here will no longer be available after the MIRA Trial ends—currently scheduled for May 2007. However, our goal is to connect participants with the best care and services available in their area so that they will already have an established relationship with local resources when the study ends.

***Specimen Storage***

Study specimens will not be stored. All specimens will be destroyed after receiving confirmed test results.

## Protocol for Prostate-Specific Antigen (PSA) Ancillary Study

*Version 3.0, May 10, 2007*

***Study name: Audio computer-assisted self-interviewing (ACASI) vs. face-to-face (FTF) interviewing – a randomized comparison using prostate specific antigen (PSA)***

**Sub-study objectives:** To compare the validity of reports on sexual behaviors obtained using two interview modes (ACASI and FTF) using prostate-specific antigen (PSA) as a biomarker for unprotected intercourse.

Specifically, we will compare the proportion of women in the two groups (ACASI and FTF) who are PSA-positive but report they have not engaged in unprotected intercourse (i.e. intercourse without a condom) in the past 48 hours.

**Research justification:** This study will document which of two approaches of asking participants sensitive questions about their recent sexual activity provides more valid responses. Valid information about sexual activity, more generally, is necessary for the interpretation of clinical trial results.

These findings will provide an assessment of the accuracy of self-reported recent sexual activity and condom use among women who exited the MIRA trial. It may inform the design of future studies that require accurate information on recent sexual behavior and provide guidance about the contribution of ACASI to increased accuracy in self-reports.

**Research methods (including recruitment plan):** Women who complete the MIRA study at the Zimbabwe site will be eligible to return to the clinic and complete the additional procedures required for this ancillary study, which will be conducted beginning in December 2006 after all of the scheduled MIRA closing visits have taken place

With a two-arm, randomized design, we will compare discordance between FTF and PSA with the discordance between ACASI and PSA. Half the participants will complete their interview FTF and the other half will complete an ACASI questionnaire. The questionnaire will assess sexual activity and method use during the previous few days, as well as product use, generally, since completion of the MIRA trial.

The desired sample size of 1,000 women could be achieved in several ways : 1) MIRA study participants are notified about the PSA study (verbally and/or with a flyer) at their closing visit for MIRA; 2) women are scheduled for the PSA study visit at their final MIRA trial visit; 3) MIRA study participants are actively recruited for the PSA study through community outreach activities that would target MIRA participants who have exited the trial; and 4) MIRA participants who return to the clinic for an additional supply of products are recruited to participate in the PSA study. With a sample size of approximately 1,000 women we would have about 80% power to detect a 30% reduction in discordance, assuming the discordance in the FTF group is 20% (one-sided test; alpha 0.05).

**Visit Procedures:**

1. We will consent women to participate in this ancillary study using an additional procedures consent form.
2. Women will complete one interview, either in a face-to-face or ACASI format. Women will be randomized to one of these interview modes.
3. Women will provide a self-collected vaginal swab for PSA testing. The swab will be self-collected using procedures developed for the MIRA trial.
4. The vaginal swabs will be air-dried and shipped in batches to the University of North Carolina, Chapel Hill for PSA testing in Marcia Hobbs’s laboratory.

**Perceived or potential repercussions of sub-study procedures/objectives on the main trial:**

Although we acknowledge that this ancillary study will require site staff to perform additional procedures (outlined above), the overall burden is relatively small compared to the importance of the additional study question addressed. Staff (e.g., site coordinator, interviewers and outreach workers) will be designated as responsible for recruitment, overseeing PSA study implementation, and ensuring timely storage and shipment of all specimens. MIRA Study coordinators and investigators will discuss the appropriate staffing requirements for this study.

**Data management & analysis plan:** To make this ancillary study less of a burden for the site, the short FTF data collection forms (DCFs) will be shipped to Family Health International (FHI) for data entry. Copies of the DCFs will remain at the site should data queries need to be resolved. The ACASI data will be accumulated by UCSF’s data management group (CIDEA). Data from the three data sources (PSA lab values, ACASI data and FTF data) will be merged into one data set at FHI for analysis. All key investigators listed below will be involved in the analysis and will have written sign-off on manuscripts/presentations.

**Estimated costs:** The primary costs are PSA testing, staffing for study implementation, and the materials involved in collecting and shipping specimens and in printing the short FTF paper interview. Other site costs may include local transport and lab technician time to package specimens for storage and shipment. We have developed a budget jointly that reflects anticipated costs.

**Funding source(s):** FHI/CDC

**Monitoring plan:** Not applicable; a local staff will be assigned primary responsibility for the oversight of this ancillary study. All data queries will be managed by this assigned staff person.

**List of key investigators (Biosketches of key investigators may be attached separately)**

Markus Steiner (FHI), Ali Minnis (UCSF), Ariane van der Straten (UCSF), Lee Warner (CDC), Maurizio Macaluso (CDC)

Site Principal Investigators: Tsungai Chipato (UZ-UCSF)

Scientific Advisors: Nancy Padian (UCSF), Kelly Blanchard (Ibis)

## Protocol for Male Partner Involvement Study

**Study Title:** The effect of male partner involvement on women’s diaphragm, gel and condom acceptability and adherence in Zimbabwe

**Prepared by:** Liz Montgomery

**BACKGROUND**

Research into cervical and chemical barriers to HIV/STIs such as the diaphragm and vaginal microbicides is underway to expand options for disease prevention. If proven effective, these female-controlled methods will be particularly useful for women in settings where male condom use is difficult because of structural-level or relationship-level factors such as culture, gender dynamics, socio-economic circumstances or relationship power. It is important to understand the role of the male partner in influencing women’s acceptability and use of these disease control methods.

**RESEARCH PROTOCOL**

**Study Aims**

The overall goal of this study is to measure and describe the effect of male involvement on women’s adherence to (i.e. uptake of) male condoms[[6]](#footnote-4) and female-controlled methods of HIV/ STI prevention. “Male involvement” encompasses the actions, preferences and attitudes of the primary male partner. The objectives of the study are below. In the data analysis section of this document there is a more in-depth description of how each aim will be addressed analytically.

**Primary aims**

1. To measure quantitatively and describe qualitatively the effect of male involvement on women’s adherence to the diaphragm, gel and male condoms.
2. To measure quantitatively and describe qualitatively the effect of male involvement on acceptability of the diaphragm, gel and male condoms.

**Secondary aims**

1. To examine whether a small intervention promoting male involvement through invitation letters and male ID cards makes an impact on a) level of male involvement; b) women’s adherence and acceptability.
2. To examine correlates of “high” vs. “low” involvement of male partners, using female and male demographic, behavioural, clinical and attitudinal data.
3. To measure whether there is an association between male involvement and STI/ HIV incidence.
4. To measure whether there is a change in different components of male involvement over time.

**METHODS**

The methods section of this document will outline the study design, data collection methods and analysis plan, including sample size considerations.

**Study Design**

All women in the MIRA study are eligible for the Male Involvement Study. Men are invited to come to the study clinics for information and/or HIV/STI counselling and testing. Early experience in MIRA showed that despite this tacit invitation, the promotion of male involvement was ineffective. As a result, a minor intervention component was added to MIRA, which includes offering every enrolled female participant an invitation letter and/or a doctor’s letter and/or a study ID card for the male partner.

The Male Involvement Study began in December 2004 with the introduction of the Male Partner Involvement Form, administered to all women enrolled in the MIRA study. Data collection will continue through September 2006. Focus group and in-depth interview data will be collected from July 2006 through March 2007.

**Data collection methods and activities**

***Data source 1: The Male Partner Involvement Form (MPIF)***

The major source of “male involvement” variables that will be used for exploring the domain of “male involvement”, and correlating male involvement with adherence, acceptability and other demographic, behavioural and clinical data are from the Male Partner Involvement Form.

This questionnaire is administered to female study participants of the Zimbabwe MIRA site (n=2500) at Months 3 and 12. The form has 58 questions. Approximately half of the responses have dichotomous yes/no responses, and the other half have continuous scales. Five items have specify lines for greater open-ended detail, e.g. “Have you invited him to come for free HIV and STI testing? If no why not (specify)________________”.

The aim of the questionnaire is to measure women’s perception of the quantity and quality of her partner’s involvement in study participation and study product use. The form attempts to explore the domains of partner communication, participation and control, and male partner’s attitudes and support for condom, diaphragm and gel use. Questionnaire items were constructed from a variety of sources, including the parent study instruments, previous qualitative studies done in Zimbabwe and other parts of southern Africa, and anecdotal discussions during pilot testing with male Zimbabwean research staff.^79-84^

The Male Partner Involvement Form was translated and back-translated into Shona from the English, and is interviewer-administered by female (to match respondents) study staff in either language, although the vast majority are in Shona (~99%). Interviewers hold tertiary-level degrees, and have a minimum of 3 years of interviewing experience. Interviewers receive extensive protocol training, form-specific training, and sign-off on SOPs which outline the intention item-by-item, of each question on each form in the study. Data will be double-entered into an Access database, and exported into SAS for analysis.

Table 1 below summarizes the anticipated sample size and the data collection points for this portion of the study, accounting for women who had already passed the first and second data collection points for the Male Involvement Study at the time of implementation.

***Table 1. Expected sample size for Male Partner Involvement Form, by study and data collection time point, (December 2004 – September 2006)***

| *Study* | **Data collection time points** | | | |
| --- | --- | --- | --- | --- |
|  | **Month 3** | **Month 6** | **Month 12** | **Total sample** |
| *MIRA** | 1160 | NA | 2237 | **3397** |
| ***TOTAL SAMPLE*** | ***1160*** | ***116*** | ***2237*** | ***3602*** |

*Form administered after start of MIRA study, therefore approx. half of the participants had already passed the Month 3 visit, and only 15 had completed the Month 12 visits. Form administered at next subsequent visit if scheduled visit missed. Estimates include 10% projected loss-to-follow-up.

**Male Questionnaire**

Originally a self-administered questionnaire to male partners of female study participants was considered. This methodology was not adopted.

***Data source 2: Existing data about male partners and female product adherence and acceptability***

In addition to the new data specifically being collected for the Male Involvement Study, data are being collected on male partners through Audio Computer-Assisted Self-Interview (ACASI) interviews and on Case Record Forms (CRFs) as part of the main clinical trial. For example, partner’s age, employment, migration, marital status, multiple partnerships, use of alcohol/drugs and violent behaviour. Questions also cover some aspects of product use disclosure and clandestine use, relationship control and decision-making. Questions about product acceptability contain partner factors in the list of potential responses women can choose.

The primary outcomes of the Male Involvement Study - women’s adherence and acceptability - will be measured from data collected in the main study, as will the HIV/STI incidence data for the secondary objective analysis. Study product adherence is notoriously difficult to measure, as it relies on self-report and is subject to several potential sources of bias, most notably social desirability and recall bias. In an attempt to minimize the former, the MIRA study uses ACASI interviews, mentioned above, which seek to encourage greater honesty (and minimize data processing error) by direct entry of potentially sensitive responses into a computer. To address the latter challenge of recall bias, participants are asked about use of each study product for three different time periods: last act of sex, sex in the past week, and sex in the past three months. Although these time periods all have strengths and limitations, triaging recall periods should contribute to an understanding of the reliability and validity of the measures.

***Data source 3: Focus group discussions (FGDs) with women and men***

The focus groups are intended to complement the quantitative analysis by describing the effect of male involvement on women’s adherence and acceptability of products. The focus groups will also capture male perspectives not in the quantitative data.

A total of 12 focus group discussions, comprising 6–12 members each, will be conducted: four with male partners of MIRA diaphragm-arm participants, and eight with female study participants (4 per arm). The main aim of these FGDs will be on diaphragm, gel and condom acceptability, feasibility and use, exploring the related issues of relationship dynamics, partner communication, contraceptive use, covert use of products, affordability and access of products.

These focus groups are part of the social science component of the MIRA trial and were not specifically designed for the Male Involvement Study. However, Liz Montgomery had substantial substantive input into the composition of the focus group guides, and is the “Principal Investigator” and will lead the analysis for the “male partners” theme group.

Sample selection - FGDs

At the final MIRA study visit, women are asked to give permission 1) to be contacted for future research related to MIRA and 2) for staff to contact their partner to participate in discussions with other men. Two waves of focus group discussions will be held with participants who have recently exited the trial. The total number who have agreed to be contacted and closed out in the past 3 months, will then be divided by the number of slots available in group discussions, such that every –nth participant will be contacted. This process will be fully documented, and the number of acceptances/refusals will be logged. FGDs will be attended by trained facilitators and note-takers, will be conducted in the local language (Shona), and will follow guides that are available in Appendix D&E. FGDs will be tape-recorded, transcribed and translated into English. Transcripts will be uploaded into Atlas.ti for coding and analysis. Thematic coding will be based on a structured coding dictionary, and will be performed by more than one person to ensure inter-coder reliability. Discrepancies will be settled by panel discussion.

***Data source 4: In-depth interviews (IDIs)***

Approximately 12-20 IDIs will be conducted with men and women to further explore the meaning of partner involvement and its impact on acceptability and adherence to study products. While focus groups are useful for gathering data on community norms, in-depth interviews should more specifically elucidate individual circumstances around the influence of male involvement and the outcomes of interest. To encourage open communication, male and female partners will be interviewed separately. IDIs will follow a semi-structured interview guide, but will allow for iteration and flexibility to probe on issues raised by the respondents. The IDI guides are not yet developed.

Sample selection - IDIs

As above, only participants who have agreed to be contacted will be considered for selection. Couples will be purposively sampled based on the degree of male involvement: Ideally a minimum of 2 with high indicators of involvement, 2 with low indicators, and 2 middle-level. Within each purposive strata, couples will be selected at random using a random number generator program. For example, if 500 women are categorized as having “highly involved” partners, the program will select 2 numbers, and the corresponding ID numbers will be contacted. In qualitative research there are no set formulas to determine an appropriate sample size. 12-20 should offer enough breadth of representation to responses, while not saturating the data. This is a commonly used approach in qualitative research, and requires that the data is reviewed in real-time as it is being collected, so that the Investigator can identify the point at which new data is no longer adding information about the research question to the existing body of data.^85^

The process for documenting sample selection, as well as transcribing, translating, coding and analyzing data will be equivalent to that done with the focus groups.

***Data source 5: Service Statistics***

As previously mentioned, men are told they are welcome to come for free HIV/ STI counselling and testing to the MIRA study, but this is only happening to a small degree (~1-3% of partners coming). From April 2006, invitation letters and doctor’s notes to excuse men from work were offered to women to give to their male partners. Documentation of services accessed during the study can be tabulated. Several analyses can then follow, for example “came to the clinic = y/n” can be created as a male involvement variable that is fed into an outcome-based model of women’s adherence. Characteristics of women whose partners came vs. did not will be analyzed.

***Data source 6: Proxy Measurements of “Male Involvement”***

In order for outreach staff to contact study participants for missed visits or STI treatment, all MIRA participants are asked to provide permission and details to be contacted at their home, through their spouse’s work, through a rural address or a through another contact. Exiting MIRA study participants complete a “Permission to Contact Form” described above, which captures permission for women or their partners to be contacted post-study participation. The number of women consenting to partner contact will be tabulated and analysed as a proxy measure of “male involvement”.

**Quantitative Data Analysis Plan**

The following is a summary of the quantitative data analysis plan, including a discussion of statistical considerations, broken down by study aim.

**Frequency and Degree of Male Involvement**

There is no standardized way of measuring male involvement, therefore a necessary first step of the analysis is to further define what male involvement is. This will be explored by tabulating the frequency and degree of male involvement reported by female participants on the Male Partner Involvement Form. These will be descriptive statistics of the proportion of female participants, together with 95% confidence intervals, who report that her partner is supportive, aware of her study participation, like the products, participate in the use of products, etc. Social science data will be coded for descriptions of what male involvement is in women’s own words, including evidence of high vs. low and negative/positive involvement. This should help to expand upon, corroborate and clarify the quantitative data. Service statistics and proxy measures of male involvement (described above) will also be tabulated to contribute to this analysis. These statistics will capture which male partners have come to the clinics, and which services, i.e. counselling, treatment, testing, they have accessed.

Salient features of “male involvement” should emerge from this exercise, such that key variables representing higher vs. lower or positive vs. negative “male involvement” may become evident. Composite variables may be constructed that incorporate more than one binary outcome. These variables will be considered as independent predictors in further analysis of acceptability, adherence and biological outcomes. These measures are not pre-constructed, and have not yet been formally validated. In the analysis process, tests of construct validity will be done, whereby individual questions are correlated with hypothesized outcomes in order to gauge their validity.

**Primary Study Aim 1: The effect of male involvement on women’s adherence to condoms, diaphragm and gel.**

The association between the degree of male involvement and adherence will be examined by the development of a categorical scale for *reported* adherence level to each study product (diaphragm, gel, condoms) during sex. Analysis of adherence will be stratified by study arm as this might confound degree of male involvement (i.e. women in condoms arm are more likely to have discussed study products with partner since he is more involved in using them). It is possible that this association may be confounded, i.e. male involvement may be lower in couples with ‘limited’ education and those with ‘limited’ education may have the lowest adherence, resulting in a spurious association between male involvement and adherence. In order to account for this, and other potentially confounding variables, a multivariate analysis will be conducted using a multiple logistic regression model in the SAS statistical package.

Focus groups and in-depth interviews with men and women may give additional insight into the association between male involvement and adherence. As described above, qualitative data will be used to define appropriate measures of “male involvement” that will be used as independent predictors of reported study product adherence. Qualitative data will inform the understanding of the outcome variable of adherence in several ways. Study participants and their partners will be asked and probed on what factors contributed to their use and non-use of each product in an open-ended fashion. Responses will complement the quantitative findings, add new information and possibly clarify discordances in the quantitative data. Qualitative findings may inform the addition of potentially confounding variables in the multivariate model, i.e. if focus group respondents mentioned that being a vegetarian was the most important determinant of diaphragm use, the model would control for dietary preference.

**Power Calculations/ Sample Size considerations for Adherence Analysis**

The MIRA sample size for the Zimbabwe site is 2500. Assuming 10% loss-to-follow-up and accounting for missing data for an additional 13 women who had already completed Month 12 before the initiation of the Male Involvement Study, the expected sample size for analysis of the association between male involvement and adherence is 2237 – 1118 in each arm of the study. The table below lists the power of the study to detect a difference in the high male involvement women vs. low male involvement, considering three ratios of high vs. low male involvement in the study population: 80/20; 60/40 and 50/50. In these calculations, three adherence level scenarios are also considered, where 90%, 70% and 50% of women with high male involvement also have “high adherence”, defined as >80% of sex acts covered by study products.

The table presents the minimum detectable relative percent difference in adherence level that the study will be able to detect with 95% confidence and 90% power between the high male involvement and low male involvement groups. In summary, the sample of 1118 women will have a high degree of power to measure relatively small differences in diaphragm and gel adherence reduction between those with high vs. low male involvement, depending on the scenarios. Condom adherence will be examined in both arms of the study, thereby doubling the available sample size and increasing the power further. In high prevalence settings, a relative “improvement” in adherence of as little as 20% between the high male involvement and low male involvement groups could have substantial public health importance and translate into thousands of infections averted, insofar as increased adherence implies a reduced probability of disease acquisition. Certainly there are other factors to consider, but willingness and ability to use disease prevention methods is a critical first step, and enhanced male involvement is a low-tech and potentially inexpensive intervention approach.

**Table 2. Power calculations for outcome of effect of male involvement on adherence and acceptability**

| **Minimum absolute percent reduction in women’s diaphragm, gel and condom adherence/ acceptability levels necessary to detect a significant difference resulting from low male involvement with 95% confidence and 90% power under three exposure and outcome scenarios, n = 2237 (1118 per arm)** | | | |
| --- | --- | --- | --- |
| **Amongst those with high male involvement, proportion of women with high adherence**  **(≥ 80% of sex acts covered)or high acceptability** | **Ratio of women with high male involvement: low male involvement** | | |
|  | **20:80** | **40:60** | **50:50** |
| **90%** | 9% | 7% | 7% |
| **70%** | 12% | 10% | 10% |
| **50%** | 13% | 10% | 10% |

(Computed using Intercooled STATA, 8.2)

**Primary Study Aim 2: The effect of male involvement on women’s acceptability of condoms, diaphragm and gel**

Acceptability of study products will be measured quantitatively in the MIRA trial by a combination of variables on the interviewer-administered Acceptability Form, the Clinical Follow-Up Form, and the ACASI questionnaires. The relationship between degree of male involvement and study product acceptability and will be studied in the same fashion as the adherence analysis above, and power calculations were computed using the same assumptions in Table 2. For acceptability, it was estimated that among women with high male involvement, 90% will have reported “high acceptability” of the diaphragm and gel. This estimate is based on the results of the Diaphragm Acceptability Study conducted in Zimbabwe prior to the MIRA trial.^86^ Thus this study will have 95% confidence and 90% power to detect an absolute percent reduction of 7-9% in the group with lower male involvement (see row 1 of Table 2 above). It is anticipated that the difference in diaphragm acceptability will be high regardless of male involvement but that condom and gel acceptability will probably be more affected by male involvement. Looking at the second and third rows in the table, the acceptability of gel and condoms amongst those with high male involvement may be closer to 50% or 70%, thus the study will have 90% power to detect an absolute reduction in acceptability of 10-12%. It is difficult to make a causal connection between an “improvement” in product acceptability and public health outcome – just because an individual reports liking a product does not mean it is used, nor that it is effective. Conversely, an individual might dislike a product (i.e. condoms) but still use them consistently for other reasons, like disease prevention. Nonetheless, acceptability is an important component of use, and it could again be argued that a relatively small improvement in acceptability indicators, such as 20% higher acceptability in the high male involvement group compared to the low male involvement group, could have important public health benefits. Similar to the adherence analysis, qualitative data will inform the examination of the association between male involvement and acceptability of each product. Qualitative data may complement or contradict the quantitative findings, and will add insight into observed relationships.

***Secondary Study Aim 1: Impact of more active male promotion on a) level of male involvement and b) women’s adherence and acceptability of products***

This component of the analysis will evaluate the success of intervention efforts to promote male involvement in the research studies through invitation letters and study ID cards for men. The intervention uptake rate will be tabulated, including a breakdown of whether men came to the clinic, accessed counselling and/or testing for STIs and/or HIV. In the qualitative portion, the study will explore reasons for coming/not coming, and what would have encouraged him, etc. A comparison of characteristics of women whose partners came/did not come will be conducted through bivariate and multivariate analyses. Secondly, this analysis will aim to address whether men coming to the research clinic, as encouraged, had a significant effect on women’s product usage or acceptability, in other words, to evaluate the effect of the intervention.

***Secondary Study Aim 2: Correlates of high vs. low male involvement among women***

In this analysis, a comparison of the characteristics of women who have more involved partners vs. less involved partners will be conducted. A multi-variate logistic regression analysis will examine predictors, such as age, educational status, religion, parity, SES, partner age differential, etc. correlated with degrees of male involvement, controlling got potentially confounding variables. This will help identify the independent characteristics of women who are more “at risk” of non-involved partners.

***Secondary Study Aim 3: Examine an association between male involvement and biological outcomes***

It would be interesting to examine the relationship between male involvement and the incidence of HIV and STIs. However, because these incidence rates are small, there is not enough power in this Male Involvement Study to measure the effect of male involvement on these biological outcomes. However, a composite variable of *any* incident infection (HIV or STI) could be created and correlated with degree of male involvement. It is estimated that HIV incidence in this population will be 4%, and that incidence of chlamydia, gonorrhea and trichomoniasis will be between 2-7% each. Allowing for overlap, whereby someone who has an incident case of gonorrhea may also have Chlamydia and/or seroconvert for HIV, the combined incidence for *any STI* will be 8%, and therefore any STI and HIV = 12%. Assuming this combined level of incidence is observed, this study will have 90% power and 95% confidence to detect a 50% relative reduction from 12% to 6% amongst those with high male involvement compared to low male involvement, under the three ratio scenarios of high vs. low male involvement in the population (80/20; 60/40; 50/50). The study will not have enough power to detect a smaller reduction in biological outcomes between those with high involvement vs. low involvement, unless the ratio of women in the study with high vs. low male involvement is close to 50/50. It is therefore not guaranteed that this study will be able to draw any significant conclusions about this association, but may be able to suggest some relationships that would warrant further investigation.

***Secondary Study Aim 4: Measure whether there is a change in different components of male involvement over time.***

The Male Partner Involvement Form will be administered at 2 time points to the majority of women. It will thus be possible to examine changes in reported male involvement over time, and correlate this with trends in product adherence and acceptability. For women whose partners come to the clinic between the time points of the questionnaire administration, it will also be possible to measure an “intervention effect”. The qualitative research will also offer insight into potential changes in both male involvement and the outcomes of interest (adherence and acceptability) over time.

**Qualitative Data Analysis Plan**

As indicated above, FGD and IDI data will be transcribed and translated into English. They will then be uploaded into Atlas.ti for thematic coding and analysis. FGD data will be analyzed as part of the larger MIRA social science component. The analysis is broken down into Theme Groups, made up of a team of data collectors, coders and analysts, and led by the Theme Group Principal Investigator. The Theme Groups relevant to the Male Involvement Study are the women’s Product Acceptability and Feasibility groups and the (Male) Partner’s Acceptability and Feasibility groups. Liz Montgomery is designated as the Theme Group Principal Investigator for the focus groups dealing with partners of trial participants, and will work closely as a Lead Analyst with the Principal Investigator for the female focus groups on the same topic. The Theme Groups will create the coding dictionary, participate in the conceptualization of research inquiry, and actively code and analyse the data. Data will be coded independently by at least 2 coders, using the pre-designated coding dictionary, and checked for at least 80% inter-coder reliability. The data will then be summarized using the designated codes, in response to the research questions.

The in-depth interviews will be analysed in a similar fashion, however they are not formally part of the MIRA social science component. Thus the coding and analysis may not be conducted as a theme-group activity, and one or two people may be responsible for guiding the research. This phase of the research will ideally come after a good proportion of the quantitative data has been entered and preliminarily analyzed, as well as some of the focus group data, so that potentially contradictory or particularly intriguing findings can be explored in the IDIs.

**Discussion of Potential Limitations**

There are a number of potential limitations in this study. The first is related to the inherent difficulty in defining and quantifying “male involvement”, as no set standard exists. This may results in misclassification bias, whereby created constructs to categorize women as having positive/high or negative/low male involvement will misclassify women. The second challenge is appending a sub-study to ongoing clinical trials, particularly MIRA. Because MIRA is an ongoing multi-site clinical trial with many Investigators, and a strict design, there is less flexibility to explore all the topics the study would like to explore, and the design of the Male Involvement Study had to be adjusted to fit within the confines of the existing studies. A third limitation relates to selection bias – because the sample is comprised of women who have opted to join a clinical trial, they may be different from women in the general community in significant ways. For example, women who join studies may automatically have more supportive spouses than most women, or they would not dare or be allowed to join a study. An additional source of selection bias may arise from the relatively small sample sizes in the qualitative research portion. Efforts are being made to select participants in a systematic way for the focus groups that should yield a quasi-random sample. The IDIs will be purposively sampled, in that women will first be stratified into a category of male involvement, and then systematically chosen. Fourth, an important component of the analysis will be related to whether male involvement effects product adherence. The adherence data collected in this study is predominantly self-reported and subject to both social desirability and recall bias, discussed previously. The data are collected through ACASI, in an attempt to encourage greater honesty by not forcing women to answer about product use to a staff member. To minimise recall bias, study product adherence is queried using three time references and response scales: last sex act, sex in the last week, and sex over the last three months. Finally, in seeking to establish a correlation between male involvement and adherence and acceptability of the diaphragm, Replens gel and condoms, this study assumes that if there are indicators of high or positive male involvement, there will be a direct and positive association with product use. However, there may be other confounding or interactive factors not considered and controlled for. A thorough investigation of relevant factors from the quantitative and qualitative data, as well as information from the literature will help to address this potential limitation.

**REFERENCES**

1. The closing visit may take place at 12 to 24 months (depending when the woman entered into the study) or at the woman’s last visit in the study, if she exits before her scheduled closing visit. [↑](#footnote-ref-2)
2. The OraQuick® rapid test (Epitope, Inc.) tests oral fluid, whole blood or serum/plasma samples for HIV. The device includes a porous pad that, after collection, is inserted into a vial containing a pre-mixed amount of developer solution. The specimen and solution flow through the testing device, and results are observable in about 20 minutes.

   ^c^ The Determine® rapid test (Abbott Laboratories) tests whole blood or serum/plasma samples for the qualitative detection of antibodies to HIV.

   ^d^ The AMPLICOR® PCR test (F. Hoffmann-La Roche Ltd.) test whole blood or serum/plasma samples by utilizing polymerase chain reaction technology and nucleic acid hybridization to detect HIV DNA. Detection of DNA has been shown to indicate presence of infection before an individual’s body has produced an antibody response (seroconversion).

   e HIV ELISA tests whole blood, serum, or plasma for antibodies to HIV by in vitro enzyme immunoassay. [↑](#footnote-ref-3)
3. [↑](#endnote-ref-2)
4. ^1^ World Health Organization (2004). Scaling Up Antiretroviral Therapy in Resource-Limited Settings: Treatment Guidelines for a Public Health Approach, 2003 revision. Available at <http://www.who.int/3by5/publications/documents/arv_guidelines/en/>. [↑](#endnote-ref-3)
5. ^2^ National Department of Health, South Africa (2004). National Antiretroviral Treatment Guideline. Available at <http://www.kznhealth.gov.za/arv/arv5.pdf>.

   [↑](#endnote-ref-4)
6. Although it is the man who must ultimately wear the condom, women’s “adherence” to condoms is a measure of how often she reports that her partner(s) used the condoms during sex with her. [↑](#footnote-ref-4)
